# Clade 2.3.4.4b highly pathogenic H5N1 influenza viruses from birds in China replicate effectively in bovine cells and pose potential public health risk

**DOI:** 10.1080/22221751.2025.2505649

**Published:** 2025-05-12

**Authors:** Junlong Xiong, Shiping Ding, Jiangtao Zhou, Yunqi Cui, Xiaona Chen, Lihong Huang, Jiahao Zhang, Wenbao Qi, Ming Liao

**Affiliations:** a Key Laboratory of Zoonoses, Ministry of Agriculture and Rural Affairs, South China Agricultural University, Guangzhou, People’s Republic of China; b State Key Laboratory for Animal Disease Control and Prevention, South China Agricultural University, Guangzhou, People’s Republic of China; c National Avian Influenza Para-Reference Laboratory, Guangzhou, People’s Republic of China; d National and Regional Joint Engineering Laboratory for Medicament of Zoonoses Prevention and Control, Guangzhou, People’s Republic of China; e Key Laboratory of Zoonoses Prevention and Control of Guangdong Province, Guangzhou, People’s Republic of China; f College of Animal Science and Technology, Zhongkai University of Agriculture and Engineering, Guangzhou, People’s Republic of China

**Keywords:** H5N1, influenza virus, cross-species transmission, cattle, zoonotic potential, evolution

## Abstract

In February 2024, H5N1 highly pathogenic avian influenza viruses (HPAIVs) of clade 2.3.4.4b were first reported in dairy cows in the USA. Subsequent multiple outbreaks on dairy farms and sporadic human infections have raised substantial public health concerns. In the same year, four H5N1 HPAIVs of clade 2.3.4.4b were isolated from ducks and geese in live poultry markets (LPMs) spanning seven provinces in China. Evolutionary analysis demonstrated that these viruses had undergone two genetic reassortments with H5 influenza viruses from wild birds in different countries. Except for 565/H5N1, the other three viruses exhibited over 99% genetic homology with avian-origin H5N1 HPAIVs from South Korea and Japan. Notably, 571/H5N1 demonstrated high replication efficiency in bovine-derived cells, particularly in bovine mammary epithelial (MAC-T) cells, and caused 16.7% (1/6) mortality in mice at a dose of 10^5^ EID_50_/50 μL, indicating its zoonotic potential. Given the potential cross-species transmission risk of H5N1 HPAIVs to cattle herds, we collected 228 serum samples from 12 cattle farms across five provinces and conducted serological testing to investigate seroprevalence of H5N1 HPAIVs in Chinese cattle herds. All tested samples were negative, indicating no widespread infection in the sampled cattle populations. However, infections in cattle from other regions cannot be ruled out. Nevertheless, due to the high mutability of H5N1 HPAIVs, enhanced surveillance of avian influenza viruses is critical to ensure timely responses to potential outbreaks.

## Introduction

Since 2021, a new variant of the H5N1 highly pathogenic avian influenza viruses (HPAIVs), belonging to clade 2.3.4.4b, has caused frequent outbreaks in wild bird and poultry populations across the globe [1–3]. Strikingly, these novel H5N1 HPAIVs have been responsible for illnesses and fatalities in more than 60 mammalian species, including seals, minks, cats, ferrets, and pigs [3–9]. The 2.3.4.4b clade of H5N1 HPAIVs exhibits three synergistic adaptive traits that markedly elevate its pandemic risk compared to other clades: (1) The dual G226L and N224 K substitutions in the HA protein [10] broaden receptor-binding specificity by altering surface antigen conformation, thereby enhancing potential for human-to-human transmission; (2) Frequent recombination with North American lineage NA genes retains a long-stalk N1 neuraminidase structure [11,12], which improves viral mobility in mammalian respiratory mucus compared to the truncated NA stalk of classical H5N1 strains [13], facilitating cross-species transmission; (3) The PB2-E627K mutation [14] stabilizes the viral RNA polymerase complex, substantially boosting genomic replication efficiency in mammalian cells and accelerating intra-host adaptation.

More alarmingly, Centers for Disease Control and Prevention (CDC)-confirmed H5N1 HPAIVs outbreaks have impacted 989 cattle herds across 17 U.S. states as of March 25, 2025. Affected dairy cows demonstrated clinical manifestations including abnormal fecal consistency, respiratory distress, and decreased milk production [15–18]. Notably, the bovine-origin H5N1 HPAIV has demonstrated transmission from infected cattle to poultry, domestic cats, raccoons, rodents, and opossums on farms [19–21] and even humans [20]. These findings collectively highlight the virus’s expanding host range, adaptation to mammalian species, and potential for sustained transmission in agricultural and domestic environments [9].

Recent studies have revealed that the cross-species transmission risk of bovine-origin H5N1 HPAIV is closely linked to its unique receptor-binding properties and tissue tropism. Although the virus can bind to sialic acid receptors (SA-α2,6) highly expressed in the human upper respiratory tract, ferret models indicate its low efficiency of human-to-human transmission [16], suggesting that the virus may currently lack efficient respiratory transmission adaptability. However, studies have detected high levels of avian-like receptors SA-α2,3-Gal-β1,4 and SA-α2,3-Gal-β1,3 in bovine mammary glands, respiratory tracts, and brain tissues, which may explain the virus's specific tropism for mammary tissue [22]. Notably, evidence suggests that bovine H5N1 may bypass traditional respiratory infection routes. In one study, kittens fed raw milk containing the virus became infected [8]. Additionally, the CDC reported multiple cases of H5N1 HPAIV infections among dairy workers, highlighting raw milk and its production chain as potential novel transmission routes for H5N1 to humans [23].

China is a major global agricultural producer with a large-scale dairy and beef cattle industry. Notably, with the significant increase in waterbird populations over the past two decades, live poultry markets (LPMs) have become frequent sites of close contact among waterfowl, poultry, mammals, and humans [24,25]. This unique zoonotic transmission interface has facilitated the spread of wild bird-origin H5 HPAIVs to poultry and mammals, leading to the persistent sporadic circulation of these viruses in China. Despite several research studies that assessed the public health risks of bovine-origin H5N1 HPAIVs in vivo and in vitro [15,17,18,24], the H5N1 HPAIVs surveillance in LPMs and dairy farms in China and their public health risk are largely unknown. Here, we collected 1,203 samples from chickens, ducks, geese, and environmental sources in LPMs across seven Chinese provinces, from which four clade 2.3.4.4b H5N1 HPAIVs were isolated. Phylogenetic analysis revealed their close genetic relationship with H5N1 HPAIVs isolated from wild birds in South Korea and Japan. We further evaluated the public health risks of these H5N1 HPAIVs through thermal stability assays, viral plaque formation, growth kinetics in different bovine cell lines, syncytium formation, and pathogenicity in mice. Finally, we collected 228 serum samples from 12 cattle farms in five Chinese provinces and conducted serological testing to assess the risk of H5N1 HPAIV prevalence in Chinese cattle populations.

## Materials and methods

### Viruses and cells

Four H5N1 influenza viruses, including A/duck/Jiangsu/565/2024 (H5N1) (565/H5N1), A/duck/Henan/567/2024 (H5N1) (567/H5N1), A/duck/Shandong/571/2024 (H5N1) (571/H5N1), and A/goose/Hebei/584/2024 (H5N1) (584/H5N1), were isolated from birds in LPMs of China during 2024. Two H5N6 viruses, including A/duck/Sichuan/21957-1/2021 (H5N6) (21957-1/H5N6) and A/chicken/Guangdong/2111062-3/2021 (H5N6) (2111062-3/H5N6), were isolated from birds in LPMs of China during 2021 as controls. H5N1 and H5N6 influenza viruses were harvested from 10-day-old specific-pathogen-free (SPF) embryonated eggs. The prepared virus stocks were aliquoted and stored at −80℃.

Madin-Darby Bovine Kidney (MDBK) cells were kindly provided by Professor Qiang Fu from Xinjiang Agricultural University; Human lung Adenocarcinoma Cells (Calu-3) were kindly provided by Shaobo Wang, PhD from Guangzhou National Laboratory Bovine mammary epithelial (MAC-T) cells were provided by Suzhou Haixing Biosciences Co., Ltd; Bovine Turbinate (BT) cells were obtained from the China Center for Type Culture Collection (CCTCC). The Vero-GFP, HEK293T, and A549 cells were stored in the laboratory. The Vero-GFP cell line was independently constructed and preserved in our laboratory. The detailed protocol is as follows: We purchased the pCDH-EGFP shuttle plasmid from the MiaoLing Plasmid Platform. Using psPAX2 and pMD2.G as the packaging plasmid and envelope plasmid, respectively, we transfected HEK293T cells at a ratio of psPAX2.G = 2:1:2 for lentivirus packaging. The supernatant was collected 48 h post-transfection, aliquoted, and stored at −80°C. For Vero cell transduction, the lentivirus was mixed with a fresh complete medium at a 1:1 ratio and applied to Vero cells, supplemented with polybrene to enhance transduction efficiency. After 24 hours of infection, the supernatant was replaced with fresh medium. Approximately 60 hours later, the cells were subjected to selection with 1 μg/mL puromycin-containing complete medium for 48 h. Monoclonal cell lines were then isolated using the limited dilution method. Finally, the cell line exhibiting the strongest fluorescence intensity was selected through fluorescence microscopy screening and cryopreserved for long-term storage***.*** The MDBK, MAC-T, and Vero-GFP cells were maintained in Dulbecco’s modified Eagle’s medium (DMEM) (Gibco), supplemented with 10% fetal bovine serum (FBS) (Excell), 100 U/mL penicillin, and 100 μg/mL streptomycin (Gibco). These cultures were maintained in a 37°C incubator with 5% CO_2_. BT cells were maintained with 10% horse serum (Gibco).

### Influenza surveillance in live poultry markets and bovine farms

Our avian influenza virus surveillance was conducted from January to June 2024 in LPMs across seven provinces in China: Hebei, Shandong, Henan, Jiangsu, Hunan, Guangxi, and Guangdong. During each sampling event, 5–10 poultry birds were randomly selected from different stalls in the markets, with a total of 20 chickens, 20 ducks, and 10 geese sampled per event. Oropharyngeal and cloacal swabs were collected from chickens and pooled into a single collection tube, while only cloacal swabs were obtained from ducks and geese. Each sample was placed into 2 mL of phosphate-buffered saline (PBS) supplemented with penicillin (5,000 U/mL) and streptomycin (5,000 U/mL), and then all samples were shipped in refrigerated express packages with ice packs to the College of Veterinary Medicine at South China Agricultural University for subsequent testing. Each sample was inoculated into the allantoic cavities of ten-day-old specific-pathogen-free (SPF) embryonated chicken eggs incubated at 37°C. All SPF chicken embryos used in the experiments were sourced from Dahuanong Biotechnology Co., Ltd., a company that has obtained GMP certification to ensure production quality. The SPF chicken embryos were incubated in a 37°C incubator, a temperature setting that supports the maintenance of the physiological environment required for normal development of SPF chicken embryos. All procedures involving avian influenza virus isolation and culture were performed under ABSL-3 containment conditions. The allantoic fluid was collected and tested via HA assay with 1% chicken red blood cells [26]. H5 influenza viruses were isolated from positive samples for whole-genome sequencing.

Bovine serum samples were collected through stratified random sampling of 10–40 cows from 12 farms across five provinces in China: Xinjiang Uygur Autonomous Region, Shandong, Tianjin, Liaoning, and Guangdong. Farm workers first categorized cattle into two age strata (calves ≤6 months; adults >6 months), then conducted sampling at a 1:4 ratio (calves to adults). Random selection within each stratum was performed for serum collection.

### Sequencing of H5 influenza viruses and phylogenetic analyses

Viral RNA was extracted from the allantoic fluid of chicken embryos using the FastGene kit (Shanghai Feijie Bio-Technology, Shanghai, China) according to the instructions. RNA was reverse-transcribed using the Reverse Transcriptase M-MLV (Takara, Beijing, China) and the primer Uni12 (5′- AGCAAAAGCAGG-3′) [27]. The whole genome segments were amplified by PCR using primers described by Hoffmann et al. [28]. The target bands were cut and sent to the sequencing company after electrophoresis on a 1% agarose gel. The sequences were aligned using Snapgene and spliced using DNASTAR software.

The corresponding reference sequences of the HA gene, collected from January 1, 2021, to October 5, 2024, were downloaded from GISAID (https://www.gisaid.org). In addition, reference sequences for other gene fragments were collected from January 1, 2023, to October 5, 2024. Multiple sequence alignments were performed using MAFFT v7, which was implemented in PhyloSuite with default parameters. Maximum likelihood (ML) phylogenetic trees were constructed using IQ-TREE v2.1.3 via PhyloSuite [29]. The best-fit nucleotide substitution model (GTR + I + G) was selected using ModelFinder. Branch support values were assessed with 1,000 ultrafast bootstrap replicates [30]. Figtree v1.4.4 and iTOL were used to complete the annotation of the phylogenetic trees. The complete coding sequence of each gene segment was analysed for nucleotide homology using MegAlign. The GISAID accession numbers of the avian influenza H5N1 virus strains used in the homology analysis are listed in Appendix Table 3.

### HI assay for detection of influenza virus antibodies in bovine sera

Whole blood collected from the farms was centrifuged (4000 g, 10 min) to obtain serum. The serum was mixed evenly with RDE (Denka Seiken) at a ratio of 1:3. After incubating at 37°C overnight (18–20 h), it was heated at 56°C for 30–60 min to stop the action of RDE. The hemagglutination inhibition (HI) test method was performed as described [31]. Chicken red blood cells (1%) were used for the HI test. Serum from chickens infected with the analysed virus strain served as the positive control, while the negative control was represented by serum from non-immune chickens and serum from chickens without avian influenza virus antibodies.

### Growth kinetics of H5 influenza viruses in different bovine-derived cell lines

The methods of growth kinetics in different cell lines were conducted as previously described [32]. Briefly, six H5 influenza viruses were measured with the 50% tissue culture infective dose (TCID_50_) on MDCK cells according to the Reed-Muench method. The TCID_50_ values of the viral strains were determined as follows: 565/H5N1, 2.57 × 10^8^ TCID_50_/mL; 567/H5N1, 6.31 × 10^8^ TCID_50_/mL; 571/H5N1, 5.62 × 10^8^ TCID_50_/mL; 584/H5N1, 1.00 × 10^9^ TCID_50_/mL; 21957-1/H5N6, 1.58 × 10^9^ TCID_50_/mL; 2111062-3/H5N6, 5.18 × 10^8^ TCID_50_/mL. Then, viral growth curves were determined in MDBK, MAC-T, and BT cells, respectively. All cell lines were infected with H5 influenza viruses at the multiplicity of infection (MOI) of 0.01 and incubated at 37°C for 1 h. The cells were then washed once with phosphate-buffered saline (PBS) and replenished with DMEM containing 100 U/mL penicillin, 100 μg/mL streptomycin, and 0.2% bovine serum albumin (BSA). The supernatants were collected at 12, 24, 36, 48, and 60 hours post-infection (hpi) and stored at −80°C before titration in MDCK cells as expressed by TCID_50_.

### Virus plaque-formation ability in MDBK cells

The plaque assay was carried out as previously described [32,33]. The MDBK cells were infected with H5 influenza viruses at the MOI of 0.0005 for 2 h. The cells were then washed once with PBS before being covered with a mixture of equal volumes of 2× concentrated DMEM (containing 0.2% BSA and 0.5 μg/mL TPCK-treated trypsin) and 2% agar and cultured for 60 h. Next, 4% paraformaldehyde fixation and crystal violet staining were used to observe the plaques. ImageJ was used to measure the number and area of virus plaques. The number of plaques is the sum of three replicate wells.

### HA thermal stability assay

The thermostability assay was performed as previously described [33,34]. The four H5N1 influenza viruses were diluted with PBS based on their HA titers and normalized to 128 HA units/25 μL. The viruses were then heated at 50°C in a water bath for different durations (0, 20, 40, 60, 120, 180, 240, 300, 360, 420, and 480 min). Subsequently, the hemagglutination activity of the heat-treated viruses was determined using a standard hemagglutination assay with 1% chicken red blood cells.

### Syncytium formation assays

The pH of fusion for H5 influenza viruses was measured by syncytium formation assays as previously described [35]. Briefly, Vero-GFP cells in 24-well plates were infected with indicated viruses at an MOI of 1. At 16–20 hpi, infected cells were maintained with pH-adjusted PBS ranging from 5.1–6.0 for 5 min. After removal of PBS, the infected cells were incubated in DMEM supplemented with 0.2% BSA for 3 h at 37°C. Hoechst (Beyotime) was used to stain the nuclei of infected cells. Micrographs of cells containing syncytia were recorded by fluorescence microscopy (Nikon).

### Mice experiment

The 50% egg infective dose (EID_50_) for the four H5N1 influenza viruses was determined using 10-day-old SPF chicken eggs, following the Reed-Muench method. Groups of twelve six-week-old female BALB/c mice (GemPharmatech, Jiangsu, China) were anesthetized with sodium pentobarbital and then intranasally infected with the four viruses at a dose of 10^5^ EID_50_ in a volume of 50 μL. Control mice were inoculated intranasally with 50 μL of DMEM. Organs from three mice in each group, including the lungs, turbinates, and brain, were collected at 3 and 5 days post-infection (dpi) for virus titration in chicken eggs, with results expressed as EID_50_, as described previously [36]. Additionally, body weight loss and mortality of six infected mice from each group were monitored over a 14-day period.

### Ethics statement

All experiments involving H5 HPAIV were conducted in the animal biosafety level 3 (ABSL-3) laboratory in accordance with South China Agricultural University (SCAU) (CNAS BL0011) protocols. BALB/c mice involved in experiments were reviewed and approved by the Institution Animal Care and Use Committee (IACUC) at SCAU and treated according to the guidelines (2017A002). Mice were housed under controlled conditions (22 ± 1°C, 12-h light/dark cycle) with ad libitum access to food and water. The remaining mice were euthanized by intraperitoneal injection of a 3% (w/v) pentobarbital sodium solution (prepared in sterile saline) supplemented with 0.5% lidocaine (local anesthetic) and 0.1 mg/kg diazepam (anticonvulsant). The dosage was adjusted to 150 mg/kg based on individual body weight measurements. Cessation of vital signs was verified through loss of corneal reflex and sustained apnea for 5 min post-injection. Death was confirmed by absence of reflexes and cardiorespiratory arrest for ≥5 min.

### Quantification and statistical analysis

All data are presented as the Mean ± standard errors of means (SEM). Student’s t-test was utilized to compare the differences between different groups. Statistical significance was indicated as * (*p* < 0.05), ** (*p* < 0.01), *** (*p* < 0.001), **** (*p* < 0.0001). All statistical analyses and calculations were performed using GraphPad Prism 9.

## Results

### Characterization of the global prevalence of H5 influenza viruses

**Figure 1.**
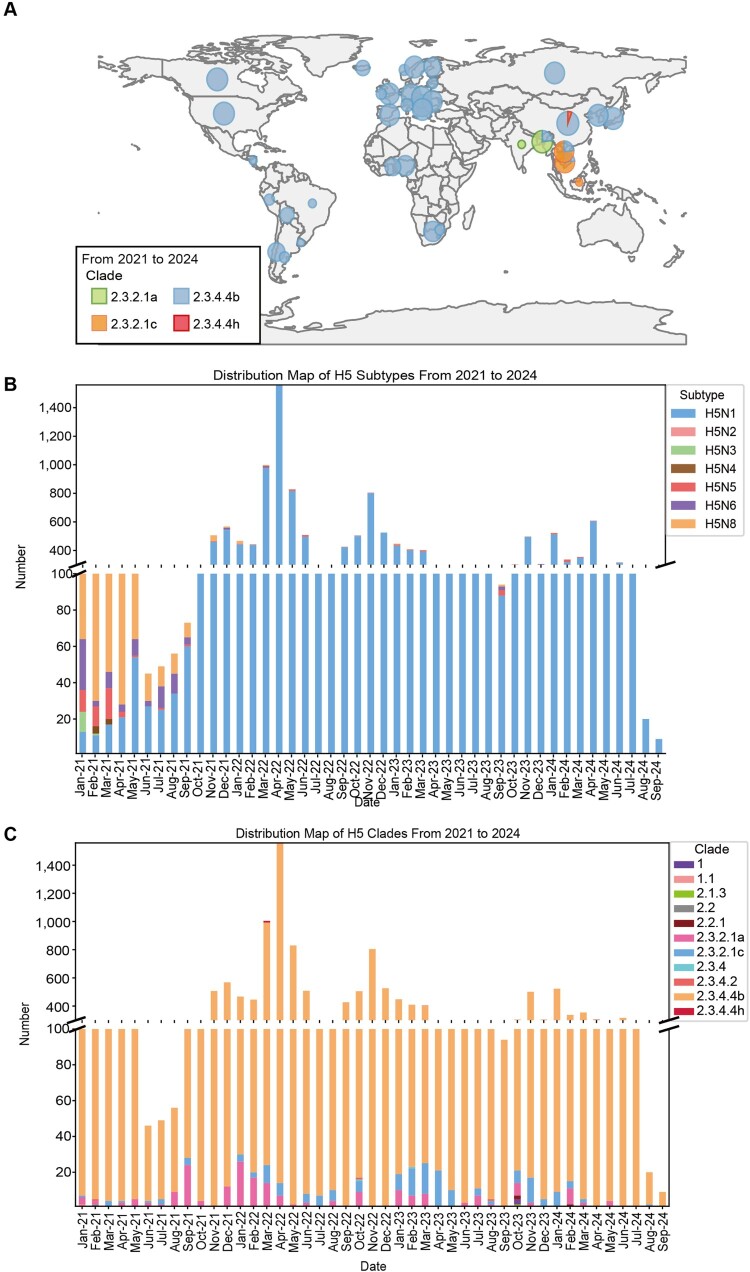
Global prevalence of H5 influenza viruses during 2021–2024. (A) Global distribution of H5 influenza viruses of different subclades in different continents. The map was drawn by R software. Green represents branch 2.3.2.1a, blue represents branch 2.3.4.4b, yellow represents branch 2.3.2.1c, and red represents branch 2.3.4.4h. (B) The number of NA subtypes of global H5 influenza viruses during 2021–2024. Blue represents H5N1, pink represents H5N2, green represents H5N3, light yellow represents H5N4, red represents H5N5, purple represents H5N6, and dark yellow represents H5N8. (C) The number of subclades of global H5 influenza viruses during 2021–2024. The colour-coded classification system assigns specific hues to each subclade: Purple represents Subclade 1, while Pink indicates Subclade 1.1. Subclade 2.1.3 is marked in Green, and another Grey shade designates Subclade 2.2. Brown corresponds to Subclade 2.2.1, followed by Light Pink for Subclade 2.3.2.1a. Subclade 2.3.2.1c is highlighted in Dark Blue, while Blue denotes Subclade 2.3.4. Additionally, Red signifies Subclade 2.3.4.2, Yellow represents Subclade 2.3.4.4b, and Dark Red is used for Subclade 2.3.4.4h.

Here, we downloaded all of the available gene segments of H5 influenza viruses during 2021–2024 from GISAID datasets and compared the characterization of the global prevalence of different subclade H5 influenza viruses. We found that subclade 2.3.4.4b H5 avian influenza viruses are widespread globally, with nearly all H5 influenza viruses in Europe, North and South America, and Africa belonging to subclade 2.3.4.4b. In some South Asian countries, such as Vietnam and Laos, subclade 2.3.2 is the predominant subclade of H5 influenza viruses, whereas the subclades 2.3.4.4b and 2.3.4.4h are predominantly prevalent in China (Figure 1(A)). It is important to note that since 2021, subclade 2.3.4.4b has been the most prevalent subclade of H5 influenza viruses in China.

**Figure 2.**
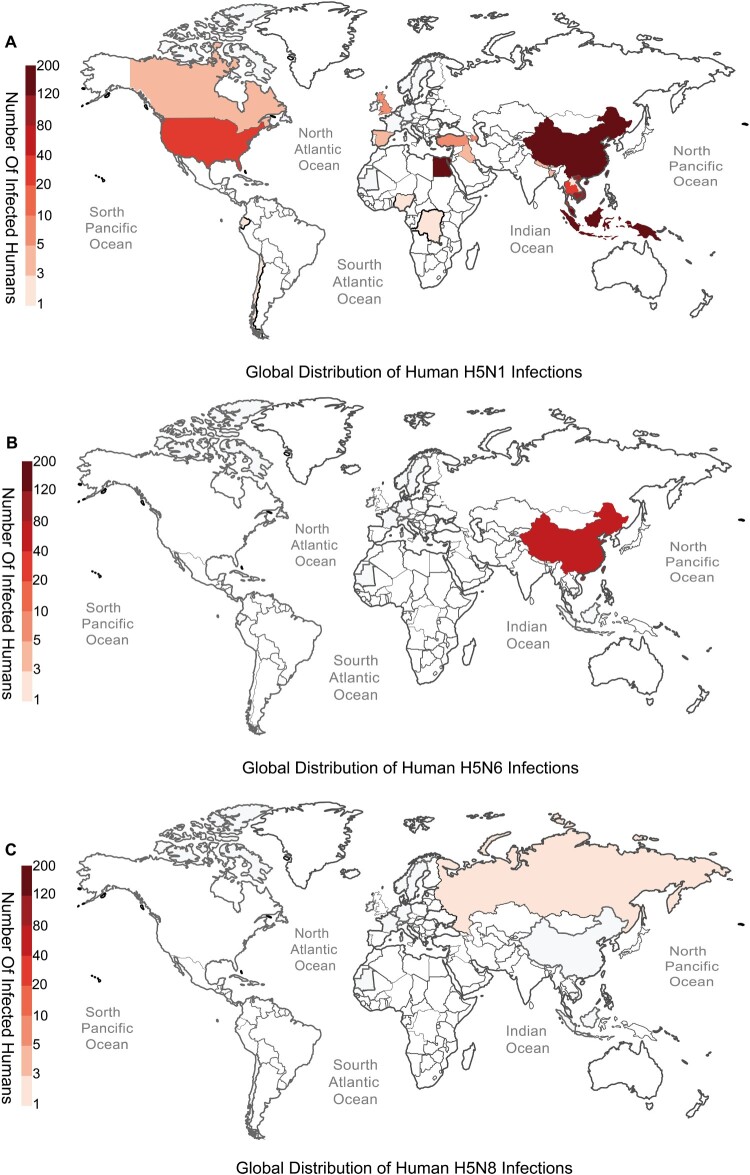
Global distribution of human infections caused by H5 avian influenza virus subtypes. Maps show the cumulative number of laboratory-confirmed human infections with H5N1, H5N6, and H5N8 subtypes, based on GISAID records. (A) The top panel illustrates the global distribution of H5N1 infections, with high numbers reported in China and several Southeast Asian and African countries. (B) The middle panel displays H5N6 infections, predominantly reported in China. (C) The bottom panel shows H5N8 infections, mainly reported in Russia. Colour intensity reflects the total number of infected individuals in each country, with darker shades indicating more cases. Data were retrieved from the GISAID EpiFlu™ database by filtering for human-origin H5 subtype entries.

Next, we characterized the temporal shifts in H5 influenza virus subclades and subtypes in recent years. Notably, a dominant subtype shift from H5N8 to H5N1 emerged in mid-2021 (Figure 1(B)). Furthermore, subclade 2.3.4.4b H5 viruses substantially outcompeted other lineages to achieve global predominance from 2021 to 2024 (Figure 1(C)). Our systematic analysis of human H5 infection patterns during this period revealed distinct epidemiological profiles: H5N1 caused widespread zoonotic transmission globally (Figure 2(A)), H5N6 demonstrated China-centric clustering (Figure 2(B)), while H5N8 maintained only sporadic human spillover in Russia (Figure 2(C)). This geographic differentiation likely stems from two synergistic drivers: (1) The enhanced fitness of 2.3.4.4b viruses facilitated their global dispersal through migratory bird networks, enabling continuous adaptation via local viral reassortment [9,19]; (2) Extensive vaccination campaigns against H5N8 may have reduced its circulation, inadvertently creating an ecological niche favouring the dominance of 2.3.4.4b-derived H5N1 variants [37].

### Isolation and evolutionary analysis of avian-origin H5N1 influenza viruses

**Figure 3.**
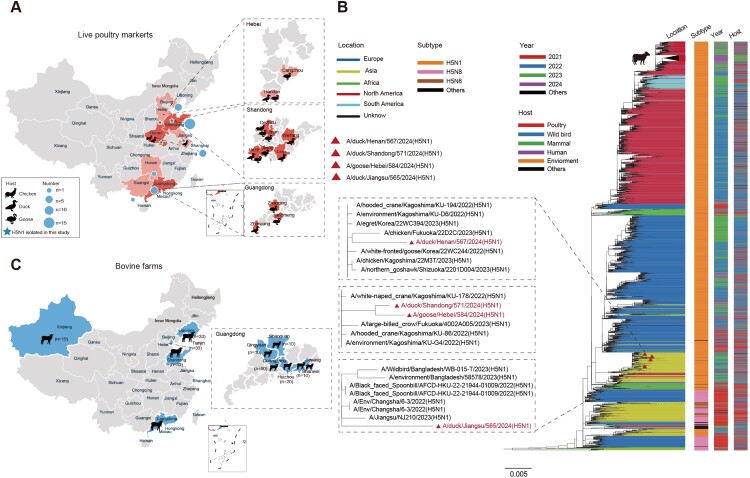
Sampling of H5 influenza viruses in birds and cattle of China during 2024 and phylogenetic analysis of H5 influenza viruses. (A) Sampling sites of H5 influenza viruses isolated from chickens, ducks, and geese in LPMs of China in 2024. (B) Phylogenetic tree of HA genes of H5 influenza viruses from 2021 to 2024. (C) Sampling sites of serum samples collected from 12 bovine farms of China in 2024. All branch lengths were scaled according to the numbers of substitutions per site. Maximum likelihood (ML) phylogenies for the codon alignment of HA gene segments were estimated using the GTR + G nucleotide substitution model in the IQ-TREE. Node support was determined by nonparametric bootstrapping with 1,000 replicates. The phylogenetic tree was graphically illustrated in the FigTree (version 1.4.3) programme (http://tree.bio.ed.ac.uk/software/figtree/). In the phylogenetic tree, four H5N1 isolated in the study were marked with red delta. It highlights the location of the virus in which the dairy cows were infected. Various line colours indicate the location of virus isolation; coloured rectangular bars on the right side of the tree indicate the subtype, the year and the host corresponding to the virus at each position in the tree, respectively.

In 2024, we conducted avian influenza virus surveillance in live poultry markets (LPMs) across seven provinces in China (Figure 3(A)), collecting a total of 1,203 samples from chickens, ducks, geese, and environmental sources (Appendix Table 2). We isolated and sequenced the full-genome sequences of four H5N1 influenza virus strains and submitted the sequences to GISAID (Appendix Table 1). Phylogenetic analysis indicated that all four H5N1 viruses belong to subclade 2.3.4.4b (Figure 3(B)). The HA and NA genes of four H5N1 viruses did not cluster together with the recently emerged bovine-origin H5N1 viruses from the USA (Figure 3(B) and Appendix Figures 1–2), but instead exhibited HA and NA homology exceeding 99% to avian-origin H5N1 viruses from Korea and Japan. Except for the NS gene, the internal genes of these H5N1 viruses were derived from wild bird origin.

H5N1 viruses are mainly from Korea, Japan, Bangladesh, and China (Appendix Figures 3–7). However, these viruses did not cluster together, indicating that these H5N1 viruses from diverse regions of China showed different evolutionary pathways. For the NS gene, three of the H5N1 viruses were genetically closely related to bovine-origin H5N1 viruses (Appendix Figure 8). These results indicate that the evolutionary pathways of H5N1 influenza viruses in China during 2024 are complex and have been reassorted with wild bird-origin H5 influenza viruses of different countries at least twice.

### Avian-origin H5N1 influenza viruses can efficiently replicate in MDBK and MAC-T cells but replicate poorly in BT cells

We selected three commonly used bovine-derived cell lines to investigate whether the novel clade 2.3.4.4b H5N1 HPAIVs can effectively replicate in bovine cell lines, including bovine turbinate (BT), Madin-Darby bovine kidney (MDBK), and bovine mammary alveolar (MAC-T) cells. The BT cell line, derived from bovine nasal turbinate, serves as a cellular model to partially reflect viral replication capacity in the upper respiratory tract during studies of specific viruses. Notably, literature indicates that MDBK cells have been widely utilized in bovine influenza virus research [38–41]. According to recent reports of U.S. H5N1 HPAIVs infections in dairy cattle–which demonstrate marked tropism for bovine mammary acinar epithelial cells–we aim to investigate whether our isolated H5N1 HPAIV strains exhibit similarly high replication efficiency in bovine mammary cells, as observed in bovine-origin H5N1 isolates [8,16,42,43]. Therefore, we focus on the replicative ability of these H5N1 HPAIVs in MAC-T cells. As controls, we selected the A549 [44] and Calu-3 cell [45] lines as reference cell lines. MDBK, BT, MAC-T, A549, and Calu-3 cells were infected with four strains of H5N1 clade 2.3.4.4b viruses, one H5N6 clade 2.3.4.4b virus, and one H5N6 clade 2.3.4.4h virus.

**Figure 4.**
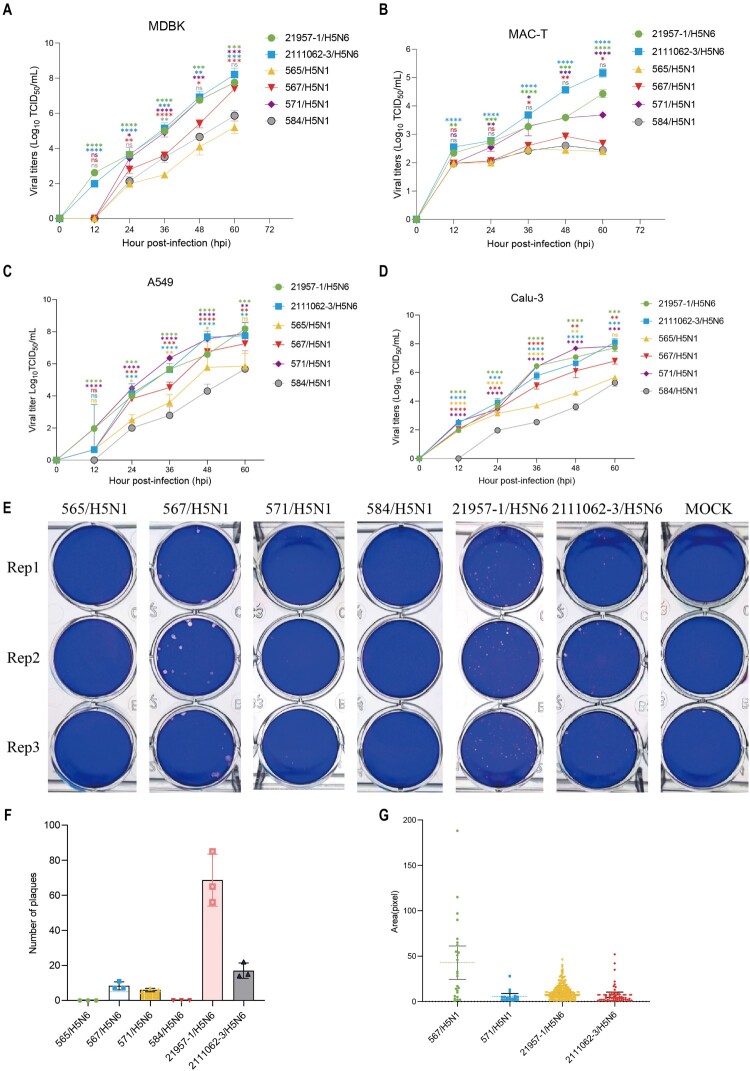
Viral replication and virulence of different H5 avian influenza viruses. Growth curves after inoculation of each H5 avian influenza viruses (21957-1/H5N6, 2111062-3/H5N6, 565/H5N1, 567/H5N1, 571/H5N1, 584/H5N1) into MDBK cells (A), MAC-T cell (B), A549 cells (C) and Calu-3 cells (D). The supernatants of infected cells were collected at the indicated time points. Data are represented as means ± SEM. The experimental data were analysed using an independent samples t-test. Prior to data analysis, normality was confirmed by the Shapiro-Wilk test (*p* > 0.05), and homogeneity of variances between groups was verified by Levene’s test (*p* = 0.12), meeting the assumptions for the t-test. Independent samples t-tests were performed to determine whether significant differences existed between other virus strains and the 565/H5N1 virus (A-B) or the 584/H5N1 virus (C-D). Significance levels are denoted as *p < 0.05, **p < 0.01, and ***p < 0.001. (E) Plaque imaging of the six H5 avian influenza viruses in MDBK cells was conducted. Plaque counting (F) and size measurements (G) were performed using Image J software, and graphs were created using GraphPad Prism 9.

As shown in Figure 4(A), all six H5 influenza viruses demonstrated significant replicative capacity in MDBK cells. Specifically, the viral titers of 2111062-3/H5N6, 21957-1/H5N6, 571/H5N1, and 567/H5N1 viruses exceeded 10^7^ TCID_50_/mL 60 hpi, showing significant differences compared to the 565/H5N1 strain. Conversely, the replicative capabilities of both 584/H5N1 and 565/H5N1 viruses were notably suboptimal (Figure 4(A)). Furthermore, although the 2111062-3/H5N6, 21957-1/H5N6, and 571/H5N1 strains exhibited detectable replication in MAC-T cells with viral titers above 10^3^ TCID_50_/mL at 60 hpi, their titers showed a 100-fold reduction compared to those in MDBK, A549, and Calu-3 cells at the same time point (Appendix Figure 11). Meanwhile, the titers of 565/H5N1, 567/H5N1, and 584/H5N1 viruses in MAC-T cells remained around 10² TCID_50_/mL, indicating limited replicative capacity (Figure 4(B)). Intriguingly, all six viruses demonstrated high-titer replication in A549 and Calu-3 cells, achieving viral titers exceeding 10^5^ TCID_50_/mL at 60 hpi (Figure 4(C,D)). Notably, 571/H5N1 virus reached peak titers as early as 48 hpi in both A549 and Calu-3 cells, with maximum titers of 10^7.5^ TCID_50_/mL, highlighting its enhanced replicative fitness and requiring heightened attention.

These results indicate that all six H5 influenza viruses replicated efficiently in MDBK, A549, and Calu-3 cells. However, only 571/H5N1, 21957-1/H5N6, and 2111062-3/H5N6 showed detectable replication in MAC-T cells, albeit with substantially lower titers compared to other cell lines. Importantly, no influenza virus was detected in any BT cell samples, confirming the non-permissiveness of BT cells to H5 influenza virus infection.

We then checked the viral plaque phenotype of H5N1 and H5N6 influenza viruses using plaque assay. Our results indicated that 21957-1/H5N6, 2111062-3/H5N6, 567/H5N1, and 571/H5N1 viruses formed a greater number of plaques in MDBK cells, with the 567/H5N1 virus generating the largest plaques (Figure 4(E–G)). Among four H5N1 viruses, 571/H5N1 and 567/H5N1 exhibited relatively strong replicative ability in MDBK and MAC-T cells, whereas 565/H5N1 and 584/H5N1 displayed less favourable performance. These findings suggest that H5N6 influenza viruses possess high viral aggressiveness, while the different H5N1 influenza viruses exhibit a range of phenotypes.

### HA acid stability and thermal stability of clade 2.3.4.4b H5N1 influenza viruses

The thermal stability and acid stability of hemagglutinin (HA) are crucial for the influenza virus's ability to survive in varying environmental conditions [46]. High thermal stability ensures that HA retains its functionality during the viral life cycle, facilitating viral entry into host cells. Acid stability allows HA to withstand the acidic environment of the endosome, which is essential for effective fusion and infection processes [46].

**Figure 5.**
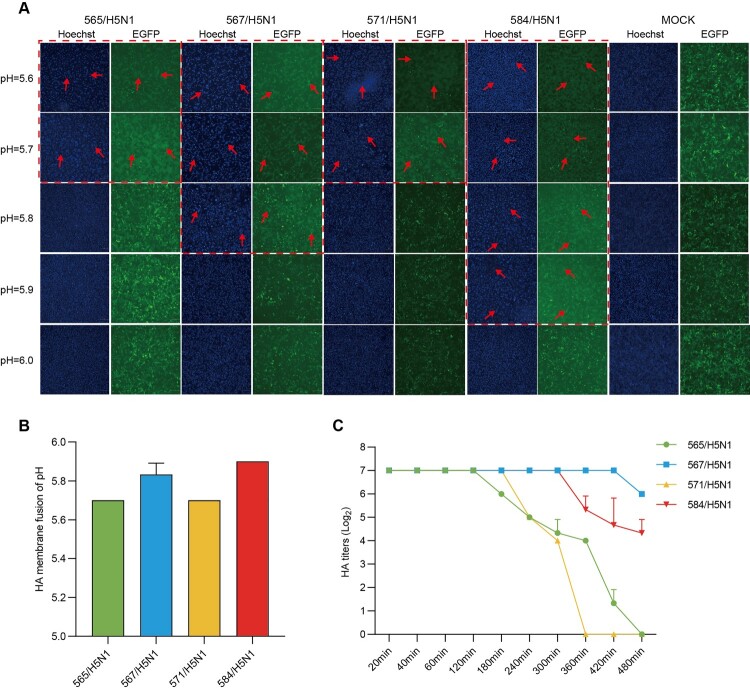
HA acid stability and thermal stability. The acid stability of hyaluronic acid (HA) was assessed using the syncytia assay. Syncytia formation was observed in Vero-GFP cells infected with influenza viruses at different pH values. (A) shows the syncytia formation for viruses 565/H5N1, 567/H5N1, 571/H5N1, and 584/H5N1 at pH values ranging from 5.6–6.0, with red arrows indicating syncytia formation. The images were captured using a fluorescence microscope (Nikon) with a scale bar of 200 μm. A bar graph (B) depicts the pH at which syncytia formation occurred for each virus based on three replicate samples. The changes in hemagglutination titer for viruses 565/H5N1, 567/H5N1, 571/H5N1, and 584/H5N1 after incubation at 50°C are shown in (C), with each sample performed in triplicate.

Here, we conducted an investigation into syncytia formation induced by four H5N1 viruses across pH levels ranging from 5.1–6.0, utilizing a GFP-expressing Vero cell line (Vero-GFP) for our experiments. Our results showed that the pH at which membrane fusion occurred for these H5N1 viruses ranged from 5.6–5.9 (Figure 5(A,B) and Appendix Figure 9). Notably, the maximum pH for membrane fusion of 571/H5N1 and 567/H5N1 viruses was lower than that of 584/H5N1 and 565/H5N1, suggesting that 571/H5N1 and 567/H5N1 were more likely to facilitate interspecies transmission.

Then, we assessed the thermostability of these H5N1 influenza viruses at 50°C using a heat block. Our results indicated that the HA titer of 571/H5N1 and 565/H5N1 viruses decreased after approximately 3 h, while 584/H5N1 and 567/H5N1 only showed a decrease after about 6 h, indicating greater stability under high-temperature conditions for 584/H5N1 and 567/H5N1 viruses (Figure 5(C)). In summary, the 567/H5N1 virus exhibited enhanced acid stability and thermostability, highlighting the resilience in fluctuating environmental conditions and potential implications for viral persistence and spread. These results suggest that strain-dependent differences in acid and thermal inactivation occurred in these H5N1 influenza viruses.

### The Clade 2.3.4.4b H5N1 influenza viruses exhibited diverse pathogenicity in mice

**Figure 6.**
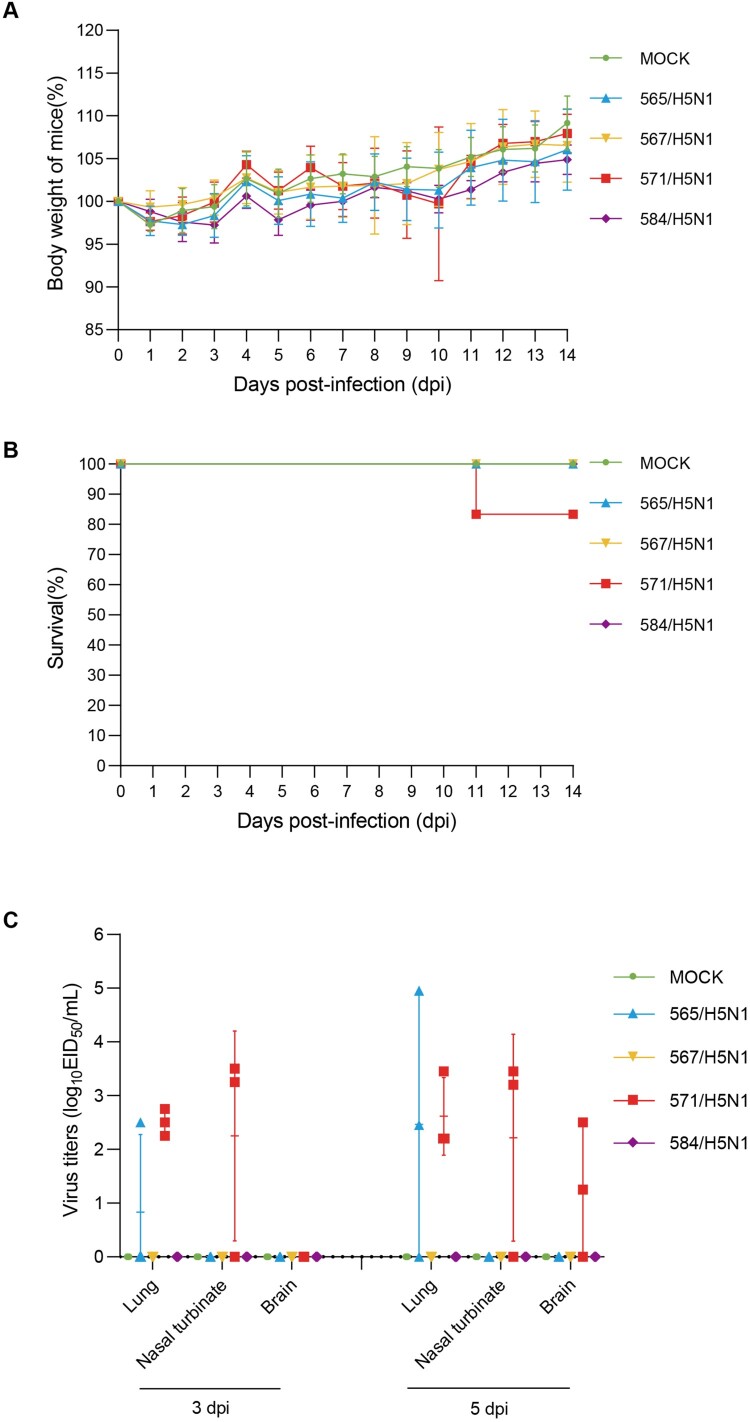
Pathogenicity of four H5N1 influenza viruses in mice. Groups of twelve mice were intranasally inoculated with four H5N1 viruses (dose: 10^5^ EID_50_/50 µL). At 3 and 5 dpi, three mice per group were randomly euthanized for organ collection (lungs, brains, and nasal turbinates). (a) Body weight changes of infected mice. (b) Survival rates of infected mice. (c) Viral titers in the lungs, brains, and nasal turbinates at 3 and 5 dpi.

We utilized a mouse model to assess the virulence of four H5N1 strains in mammals. Mice were intranasally injected with 565/H5N1, 567/H5N1, 571/H5N1, and 584/H5N1 at a dose of 10^5^ EID_50_/50 μL to observe their pathogenicity. No significant differences were observed in weight changes between all infected groups, with a gradually increasing trend, and almost all infected mice survived, except one in the 571/H5N1 group, which died (Figure 6(A,B)). Additionally, three mice from each group were sacrificed to measure the viral titers in the lungs, nasal turbinates, and brains at 3 and 5 dpi. Infectious virions were detected in the nasal turbinates and lungs of mice in the 571/H5N1 group at both 3 and 5 dpi (Figure 6(C))

### No specific antibodies against the H5 strain of influenza were found in the bovine herds.

We identified antibodies specific to H5 and H7 influenza viruses in bovine herds in China based on samples collected in 2024. Our serological survey involved the analysis of 228 serum samples collected from 12 bovine farms across the provinces of Xinjiang Uygur Autonomous Region, Guangdong, Shandong, Tianjin, and Liaoning (Figure 3(C)). We employed the hemagglutination inhibition (HI) assay to test for H5 clade 2.3.4.4b, H5 clade 2.3.4.4h, and H7N9 influenza viruses, which are currently circulating strains in China. The findings indicated that all serum samples obtained from these 12 farms tested negative for H5 and H7 influenza viruses (Table 1). This suggests that H5 and H7 avian influenza viruses have not widely transmitted to bovine herds in the sampled regions.

**Table 1.** Results of hemagglutination inhibition antibody detection for H5 and H7 subtypes of avian influenza virus in bovine serum samples from 12 cattle farms across five provinces in China.

Group		23548/H7N9 (n/N)	2111062-3/H5N6 (n/N)	21957-1/H5N6 (n/N)
Xinjiang		0/19	0/19	0/19
Shandong		0/33	0/33	0/33
Tianjin		0/33	0/33	0/33
Liaoning		0/33	0/33	0/33
Guangdong	Farm 1	0/10	0/10	0/10
	Farm 2	0/10	0/10	0/10
	Farm 3	0/10	0/10	0/10
	Farm 4	0/10	0/10	0/10
	Farm 5	0/10	0/10	0/10
	Farm 6	0/20	0/20	0/20
	Farm 7	0/20	0/20	0/20
	Farm 8	0/20	0/20	0/20

Group		565/H5N1 (n/N)	567/H5N1 (n/N)	571/H5N1 (n/N)	584/H5N1 (n/N)
Xinjiang		0/19	0/19	0/19	0/19
Shandong		0/33	0/33	0/33	0/33
Tianjin		0/33	0/33	0/33	0/33
Liaoning		0/33	0/33	0/33	0/33
Guangdong	Farm 1	0/10	0/10	0/10	0/10
	Farm 2	0/10	0/10	0/10	0/10
	Farm 3	0/10	0/10	0/10	0/10
	Farm 4	0/10	0/10	0/10	0/10
	Farm 5	0/10	0/10	0/10	0/10
	Farm 6	0/20	0/20	0/20	0/20
	Farm 7	0/20	0/20	0/20	0/20
	Farm 8	0/20	0/20	0/20	0/20

Note: The data in the table are presented as positive samples/total samples (n/N).

## Discussion

In recent years, novel H5N1 HPAIVs have been endemic in wild birds and poultry globally [47–49], causing a large number of mammal infections, including humans [3,15,50,51], which poses a significant threat to agricultural production and public health. This concern is exacerbated by their continuous adaptation and reassortment among different host species [52]. These novel H5N1 HPAIVs of clade 2.3.4.4b first emerged in 2020, spreading rapidly in wild bird populations from Europe to Africa [53], Asia [54], North and South America [15]. These viruses have led to recurrent outbreaks in fur farms in Europe [2], marine mammals in South America [49], and dairy cattle in the USA [15]. Of particular concern is that starting in February 2024, the outbreaks of B3.13 genotype H5N1 HPAIVs were frequently found in multiple dairy cattle farms in the United States [15–17]. Phylogenetic analysis demonstrated that these bovine-origin H5N1 HPAIVs originated from wildlife-origin H5 HPAIVs [15], indicating that the virus has jumped from wildlife into cattle that adapted to new hosts.

During our routine influenza surveillance in LPMs in China in 2024, four strains of H5N1 HPAIVs were isolated from the collected samples. Evolutionary analysis showed that all of them belonged to the 2.3.4.4b. Apart from 565/H5N1, they exhibited close genetic relationships with avian-origin H5N1 HPAIVs from South Korea and Japan, showing a high homology exceeding 99%. However, they demonstrated significant genetic divergence from cattle-origin H5N1 viruses, with homology of approximately 96%, indicating their evolutionary pathways are independent of the bovine-origin H5N1 HPAIVs in the United States. It is noteworthy that China serves as a critical node in two major migratory bird networks-the East Asian-Australasian-Flyway [55] and the Eastern China Flyway [56]. The migratory routes of these birds span vast areas, covering Northeast China, northern China, eastern coastal regions, and the coastal zones along the Yellow Sea and Bohai Sea. Analysis shows that the locations where H5N1 HPAIVs were isolated–China's Jiangsu, Shandong, Henan, and Hebei provinces–are geographically proximate to these flyways. This suggests that the H5N1 HPAIVs identified in multiple LPMs across China may have been introduced via these migratory pathways. Furthermore, there is potential for the viruses to spread along these routes to Southeast Asia and even Oceania, though further experimental evidence is required to confirm this hypothesis.

Given recent studies revealing the extensive prevalence of H5N1 HPAIVs in dairy cattle across the United States, this study evaluated the replication capacity of H5 subtype influenza viruses in bovine-derived cells (MDBK, MAC-T, and BT cells). The results demonstrated that all H5 viruses failed to achieve efficient replication in BT cells, while 571/H5N1, 2111062-3/H5N6, and 21957-1/H5N6 exhibited robust replication capabilities in both MAC-T and MDBK cells (Figure 4). These findings suggest a lower risk of H5 influenza virus transmission through the respiratory route in cattle populations. However, vigilance is warranted regarding the potential cross-species transmission risk posed by H5N1/H5N6 viruses from clades 2.3.4.4b and 2.3.4.4h via mammary gland infection. Notably, although these viruses could infect MAC-T cells, their viral titers were significantly lower than those observed in MDBK, Calu-3, and A549 cells (Appendix Figure 11). This contrasts with North American cattle-derived H5N1 HPAIVs, potentially attributable to lower expression levels of avian-type SA-α2,3-Gal-β1,4 and SA-α2,3-Gal-β1,3 receptors on MAC-T cells, suboptimal conformational configurations for viral attachment, or the absence of key mammalian adaptation mutations such as PB2-E627K [57] and HA-Q222L/N224K [10], which may restrict their replication efficiency in MAC-T cells (Appendix Table 4).

Previous studies have shown that bovine-origin H5N1 HPAIVs possess dual human- and avian-type receptor-binding specificity with limited respiratory droplet transmission in ferrets [16], indicating the potential risks of H5N1 HPAIVs to infect humans. Therefore, we assessed the pathogenicity of clade 2.3.4.4b H5N1 HPAIVs recently isolated in China. In this study, we observed that poultry-origin H5N1 HPAIVs showed moderate virulence in mice, and 571/H5N1 was lethal to mice (Figure 6). Prior research showed that bovine-origin H5N1 HPAIVs were lethal to mice [13]. These results indicate that although clade 2.3.4.4b H5N1 HPAIVs have the ability to infect dairy cows, the risks of the cross-species transmission of these poultry-origin H5N1 HPAIVs in China are still low. However, due to the rapid evolution of H5 influenza viruses that can be well adapted to mammals, comprehensive H5 influenza surveillance in birds and mammals should also conducted in future studies.

The thermal stability of avian influenza viruses has always been a topic of great interest among scientists. Numerous researchers have conducted studies in this field. Zhang et al. found that H5N8 viruses can persist long-term in contaminated wild bird feces and low-temperature muddy water, which may serve as potential sources for transmission to humans and free-range poultry [34]. Palme et al. demonstrated that bovine-origin H5N1 HPAIVs are remained infectious on milking machines for up to 3h [58]. Based on existing research findings, we have noted with concern that the US cattle-origin H5N1 virus may be transmitted to felines through raw milk consumption [8]. This phenomenon indicates that H5N1 HPAIVs have acquired the capability for dairy product-mediated transmission.

Considering that high-temperature sterilization may significantly compromise the nutritional value of fresh milk, milk is generally pasteurized. Cui et al. and colleagues discovered that the standard pasteurization methods employed by dairy companies effectively inactivate all tested subtypes of influenza viruses present in raw milk [59]. Our thermal stability experiments revealed that both 567/H5N1 and 584/H5N1 maintained infectivity even after 8 h of treatment at 50°C. Although this temperature remains substantially lower than the Low Temperature Long Time (LTLT) standard (62–65°C), improper operational procedures or insufficient processing duration during pasteurization could potentially allow residual H5N1 HPAIVs to retain partial infectious capacity. During the milking process, dairy workers may come into direct contact with the virus during fore teat ripping, attaching milking machines, and cleaning cow teats, potentially allowing viral entry through mucous membranes such as the conjunctiva [60]. Therefore, enhanced biosecurity measures should be strictly implemented during these procedures, including the proper use of personal protective equipment (PPE) such as goggles, masks, and disinfected gloves, along with rigorous adherence to hand hygiene protocols and equipment disinfection routines. This finding also implies that H5N1 HPAIVs with elevated thermal stability might persist stably in environmental samples (e.g. water bodies, soil, dust, and equipment) within LPMs for extended periods, contributing to sustained viral circulation. These insights underscore the necessity to enhance surveillance of environmental and biological samples beyond host-specific monitoring. Furthermore, it highlights the urgent need to improve disinfection protocols in LPMs to prevent persistent avian influenza outbreaks.

In order to investigate the prevalence of H5N1 HPAIVs in bovine herds across China, we initiated a serological monitoring programme targeting cattle populations in five provinces. Our results suggested that all of the serum samples collected from bovine farms tested negative for H5 influenza viruses (Table 1). These findings indicate that, although frequent outbreaks of H5N1 HPAIVs have occurred in dairy cattle farms in the USA, the potential risk of H5 influenza virus infections in cattle is currently low in China. Due to limited influenza surveillance, we cannot rule out the possibility that H5 influenza viruses may have caused cow infections outside the sampling sites in China.

H5-subtype avian influenza vaccines have demonstrated remarkable efficacy in curbing viral transmission, as exemplified by China's domestically developed H5-Re14 vaccine [37], which successfully contained the H5N8 HPAI outbreak, fully substantiating its public health value. However, with the continuous evolution of the 2.3.4.4b clade H5N1 HPAI virus, the protective efficacy of current vaccine strains against this variant has shown a significant decline. We recommend expediting the screening of novel vaccine candidate strains targeting this epidemic clade to establish preemptive safeguards for outbreak containment. Notably, emerging epidemiological evidence indicates that the 2.3.4.4b H5N1 virus has breached interspecies barriers, establishing transmission chains among mammals in close contact with humans, including felids, ruminants, and swine. To address this, we propose establishing a multidimensional surveillance system: building upon traditional monitoring sites such as LPMs and migratory bird habitats, priority should be given to implementing active surveillance in rural communities with mixed poultry-livestock farming practices, thereby constructing an early-warning network covering zoonotic transmission hotspots.

Given the virus's cross-border transmission potential rooted in its biological characteristics, we specifically advocate for global collaboration under the WHO's One Health framework. This should include establishing an international vaccine strain-sharing mechanism, harmonizing surveillance technical standards, and conducting joint research on transboundary avian migration patterns. Such multidimensional cooperation will significantly enhance global responsiveness to emerging variants, ultimately forging robust defenses to safeguard agricultural production security and human health.

## Limitations

Although we demonstrated that H5N1 HPAIVs isolated from LPMs efficiently replicate in bovine-derived cells, it should be noted that cell models cannot recapitulate the holistic physiological status of animals or simulate authentic host responses during viral infection. Therefore, conducting challenge experiments in cattle remains necessary. Additionally, our serological survey was limited in geographical coverage and sample size (228 samples collected from 12 dairy farms across five provinces), which may not fully represent the actual H5N1 HPAIV infection status in other regions. Expanded surveillance with broader geographical sampling is urgently required to monitor potential H5N1 HPAIV spread within Chinese cattle populations. Furthermore, given existing evidence suggesting that H5N1 HPAIV transmission in US dairy cattle primarily occurs through milk-related routes, future investigations should incorporate raw milk sampling into surveillance protocols.

## Conclusion

In summary, we isolated four H5N1 HPAIVs belonging to clade 2.3.4.4b from LPMs in China. The study revealed that all H5N1 isolates exhibited efficient replication in bovine-derived MDBK cells, but only 571/H5N1 strain demonstrated effective replication in MAC-T cells and displayed lethality (16.7%) in mice, which warrants high vigilance. Furthermore, testing of serum samples collected from multiple cattle farms across China showed all samples were negative. These results indicate that, within our detection scope, the currently circulating H5N1 HPAIVs in Chinese LPMs have not yet achieved widespread transmission in cattle populations.

## Supplementary Material

Appendix Figure 7.jpg

Appendix Figure 6.docx

Appendix Figure 2.docx

Appendix Table 3.docx

Appendix Table 1.docx

Appendix Figure 9.jpg

Appendix Figure 3.docx

Appendix Figure 5.docx

Appendix Figure 4.docx

Appendix Table 4.docx

Appendix Figure 10.docx

Appendix Figure 11.jpg

Appendix Figure 1.docx

Appendix Figure 8.docx

Appendix Table 2.docx
