# Supplementary figures and images for: Clade 2.3.4.4b highly pathogenic H5N1 influenza viruses from birds in China replicate effectively in bovine cells and pose potential public health risk
Source: Emerg Microbes Infect. 2025 May 12;14(1):2505649. doi: 10.1080/22221751.2025.2505649 (PMC12128135; doi:10.1080/22221751.2025.2505649)

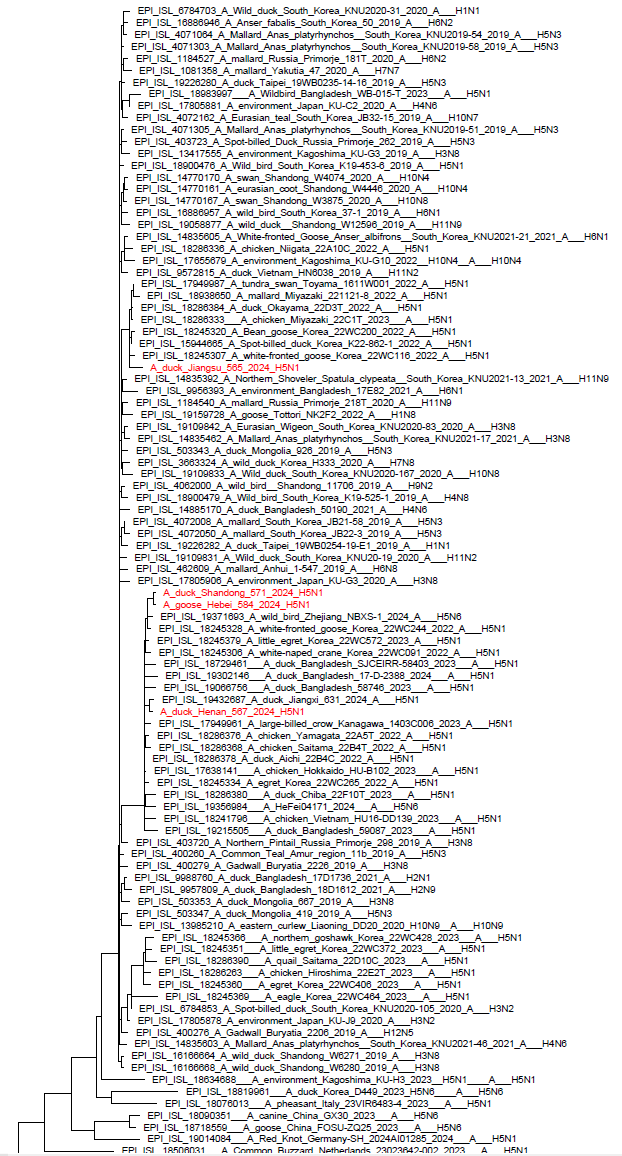


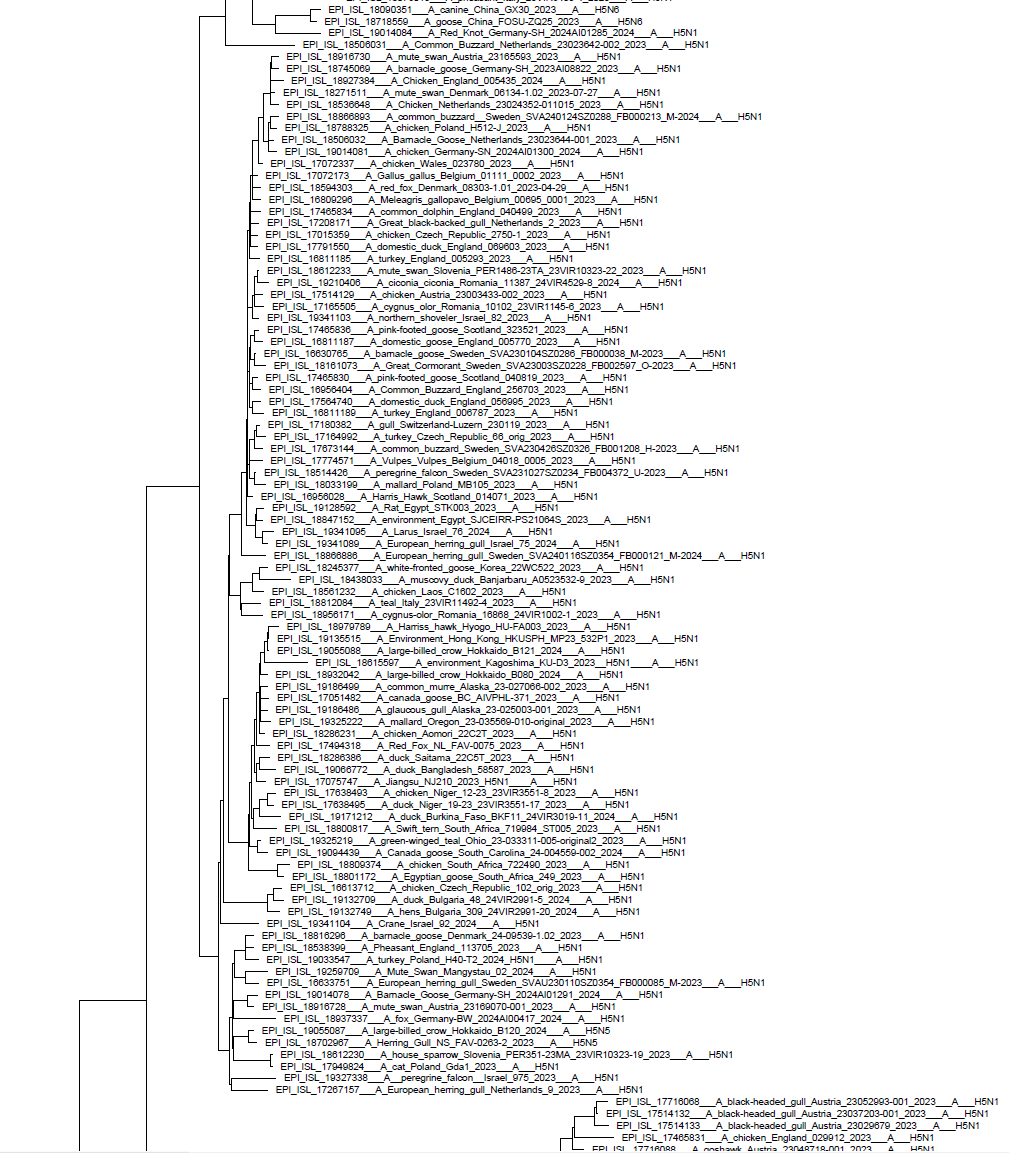


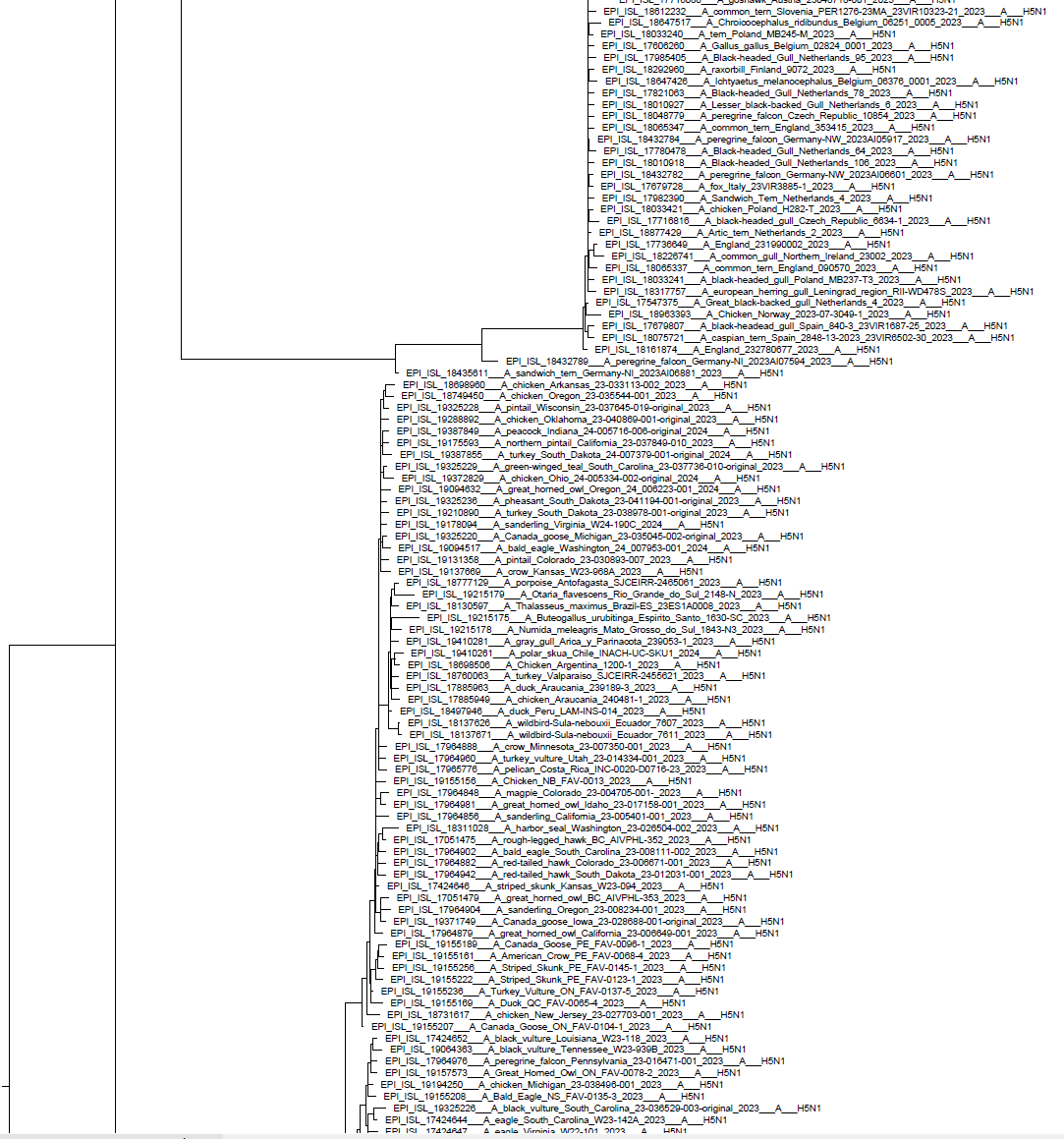


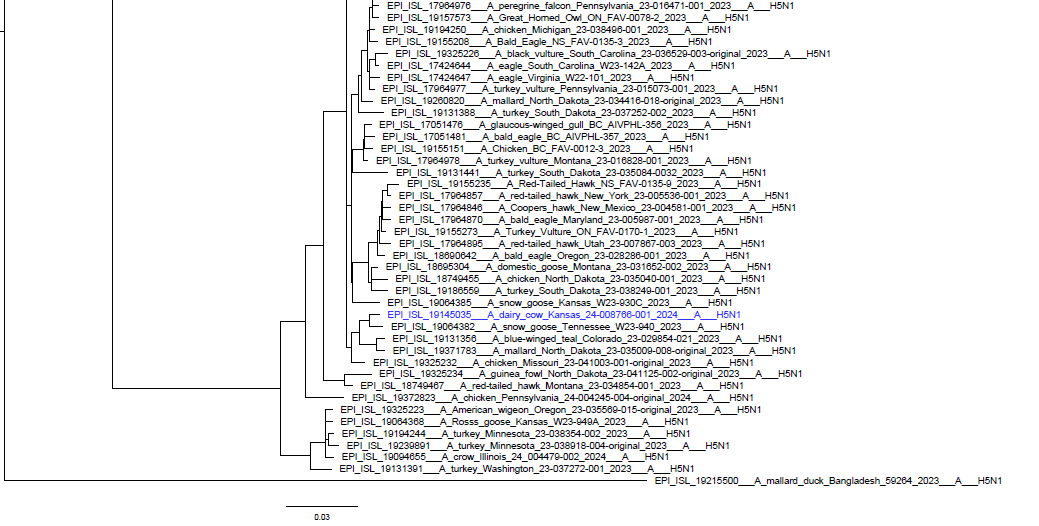

Supplement: Appendix Figure 6.docx [file TEMI_A_2505649_SM3535.docx]

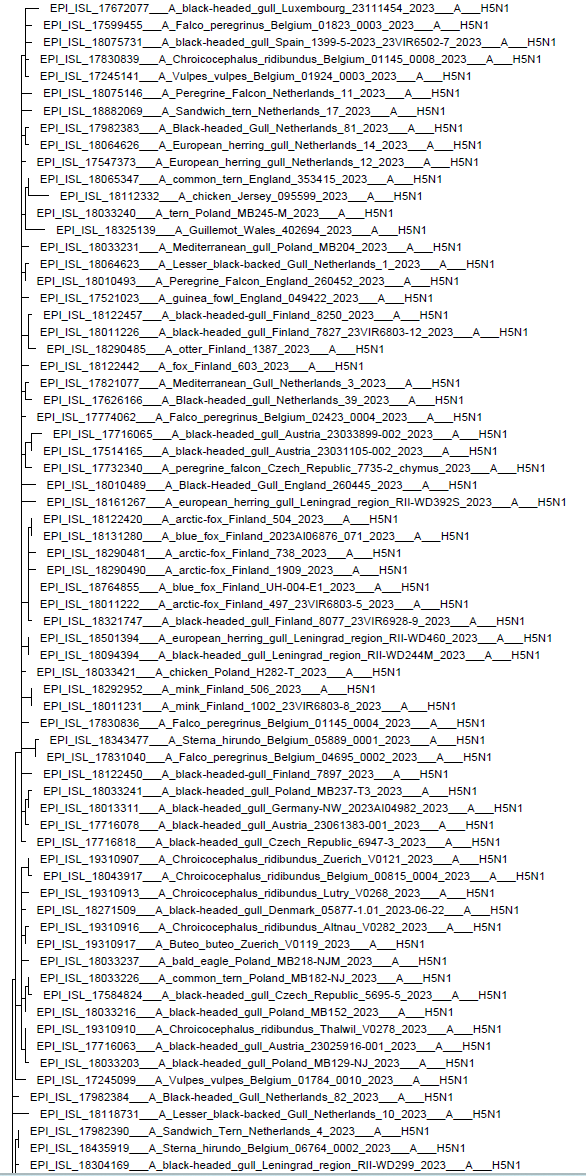


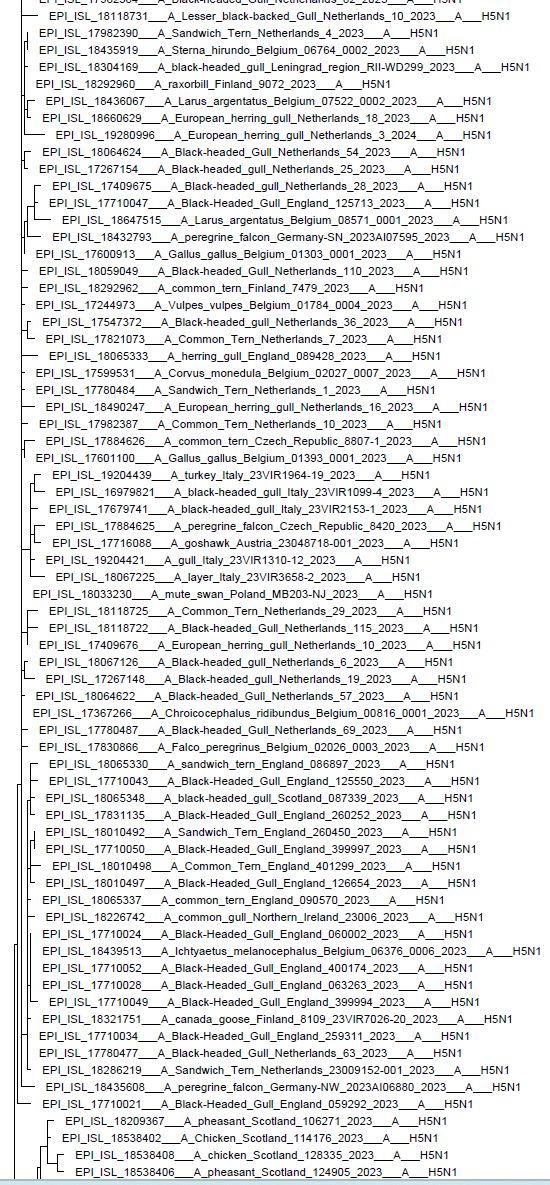


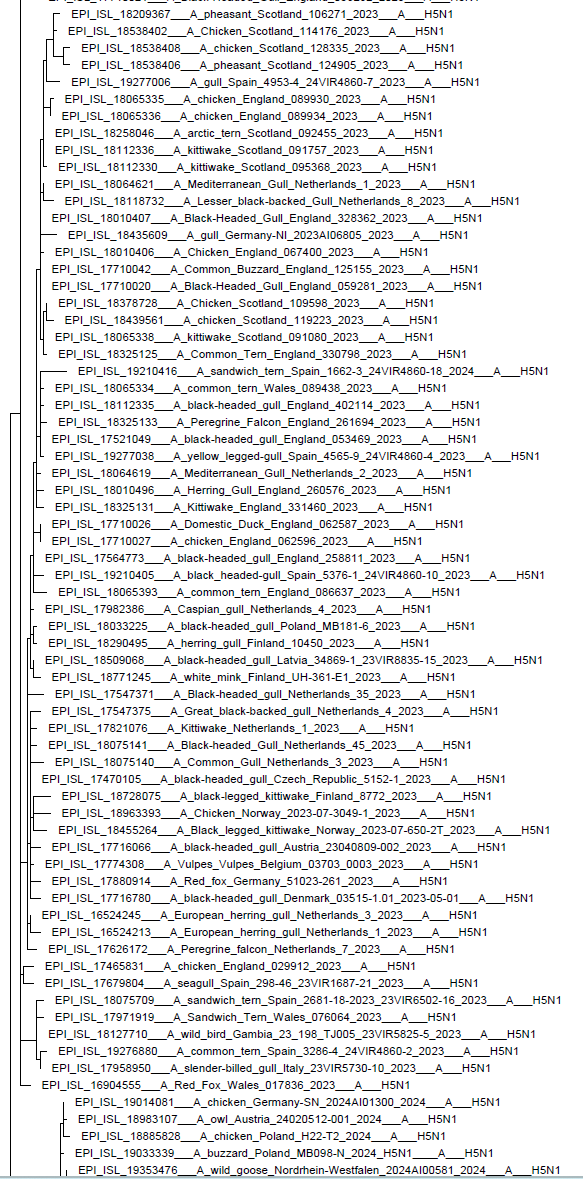


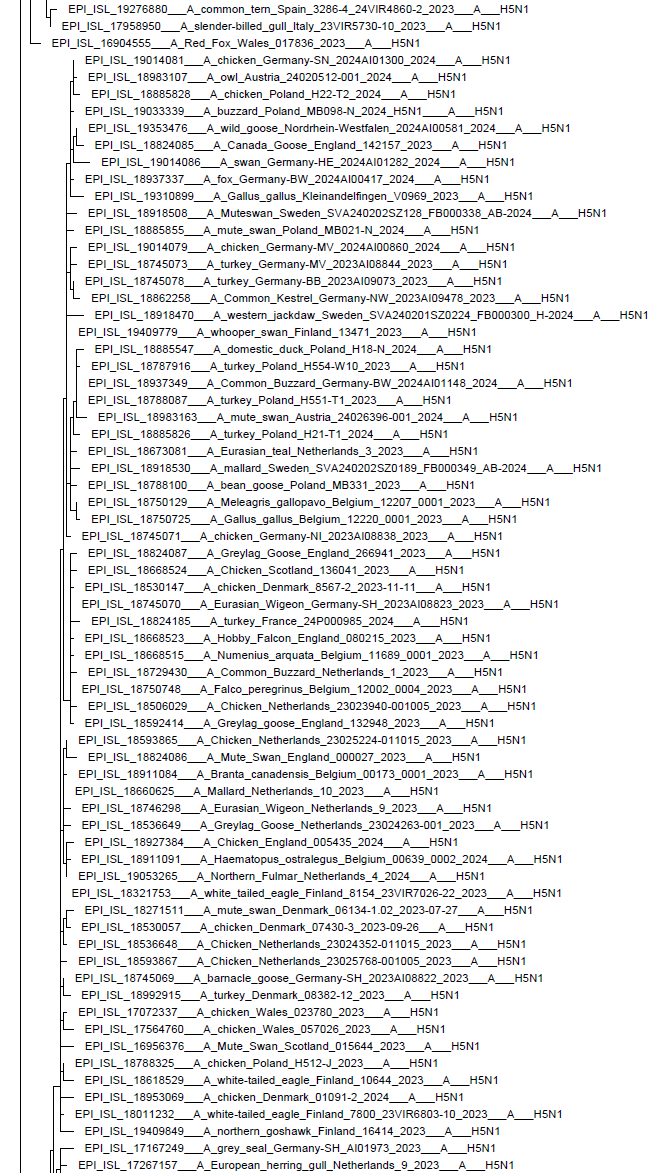


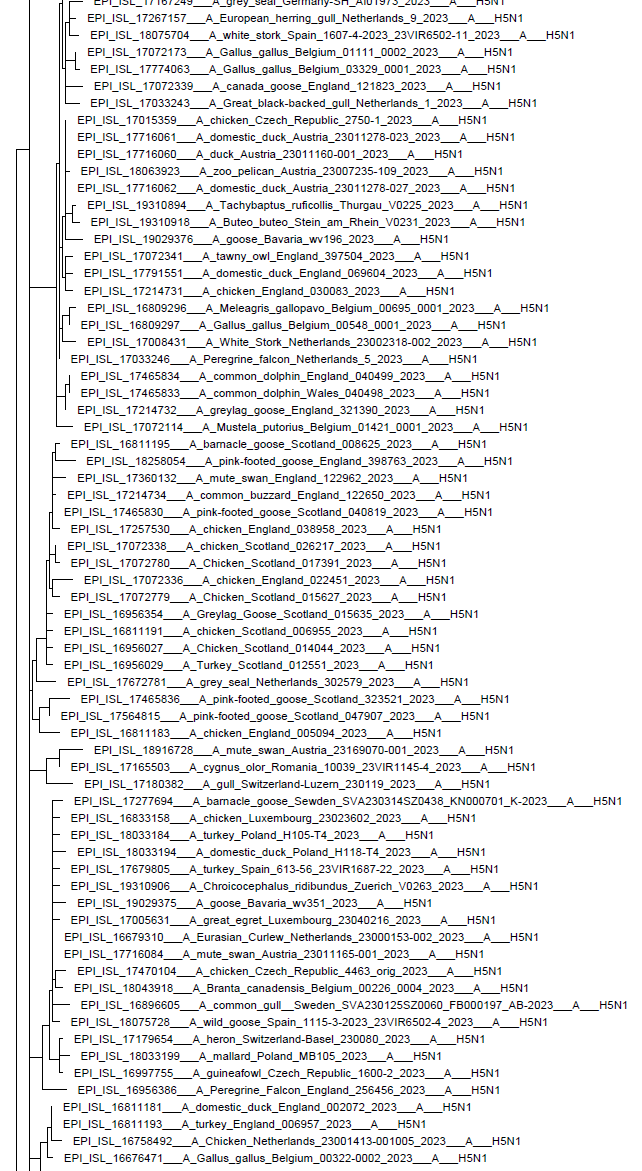


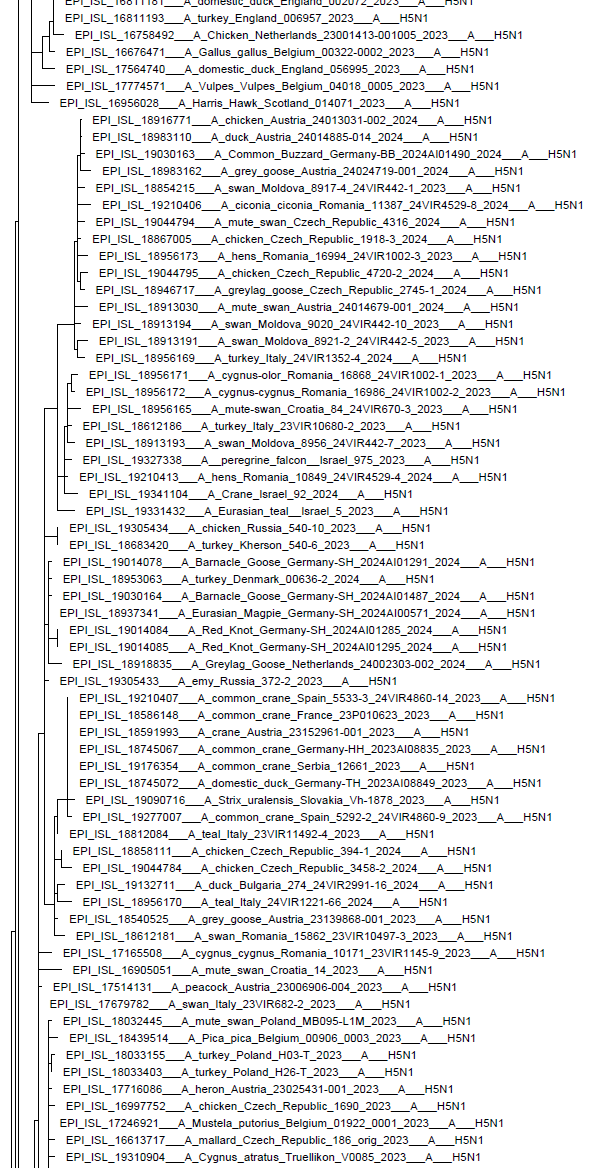


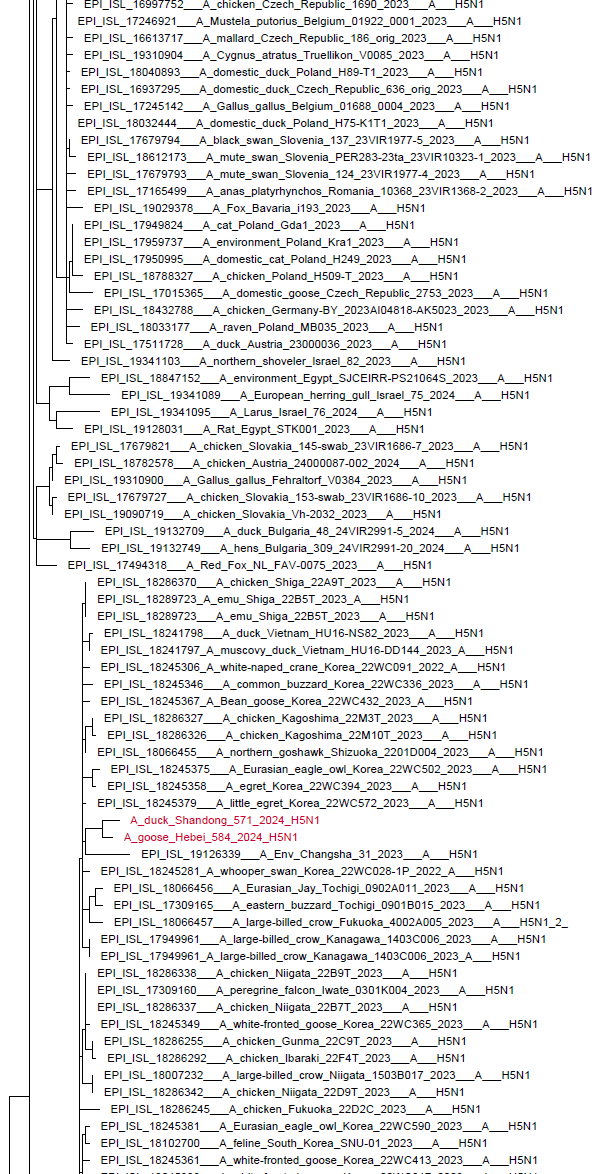


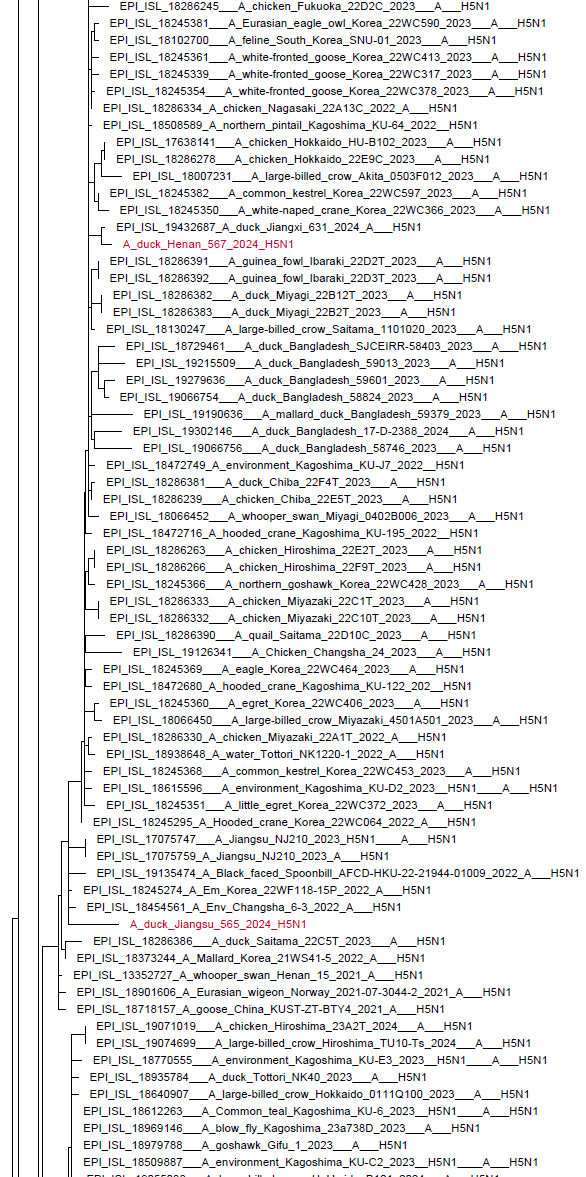


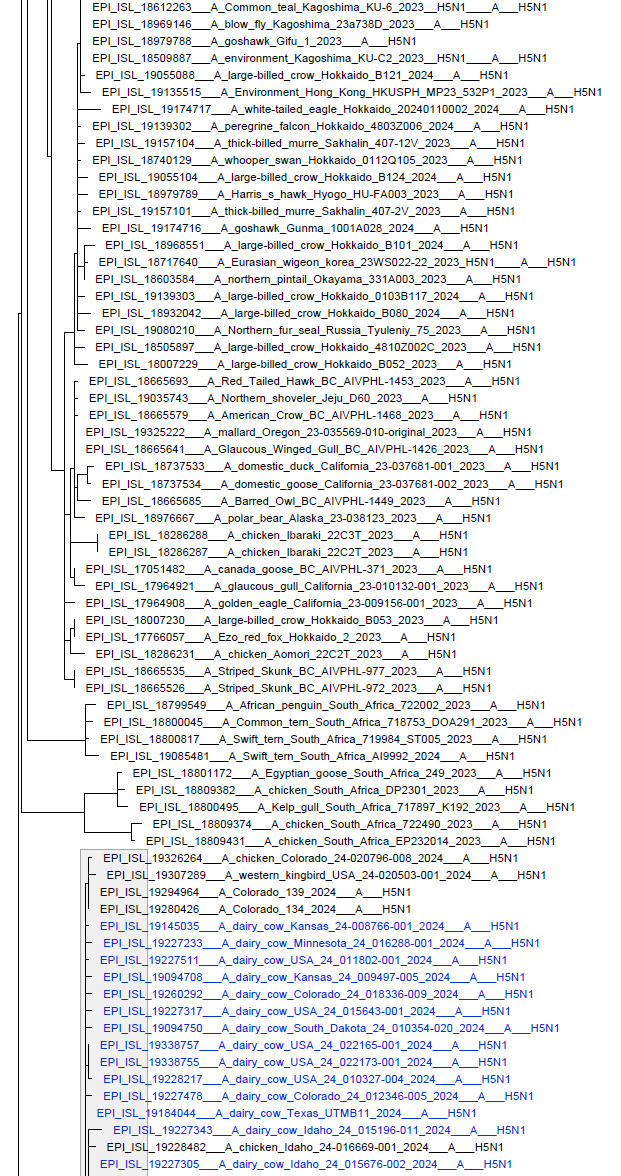


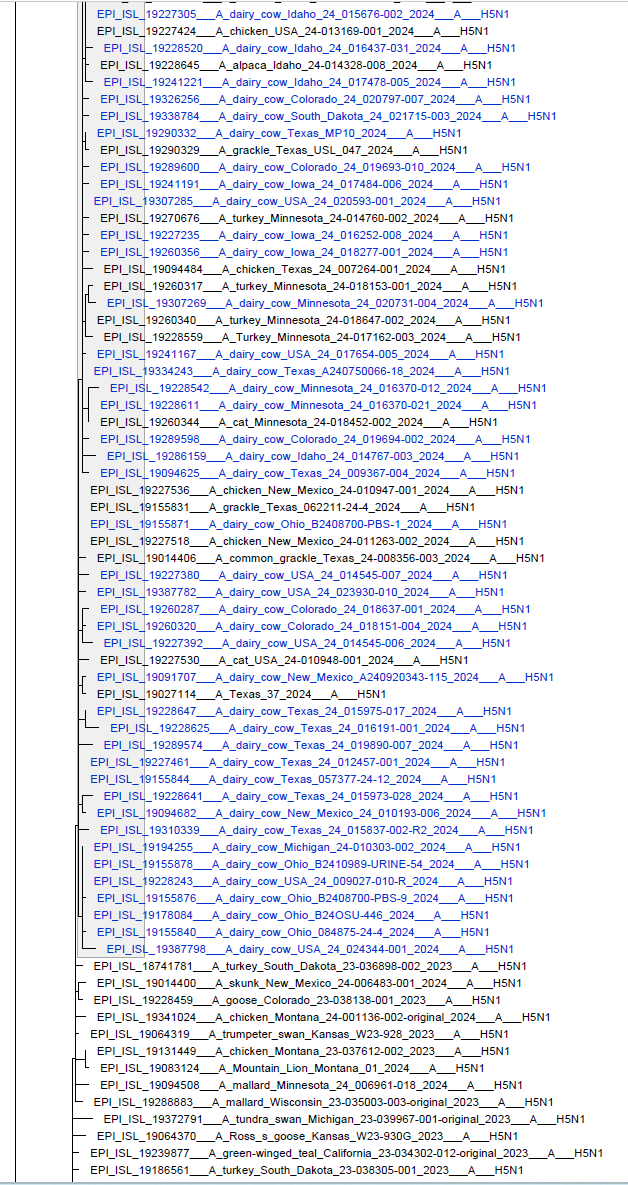


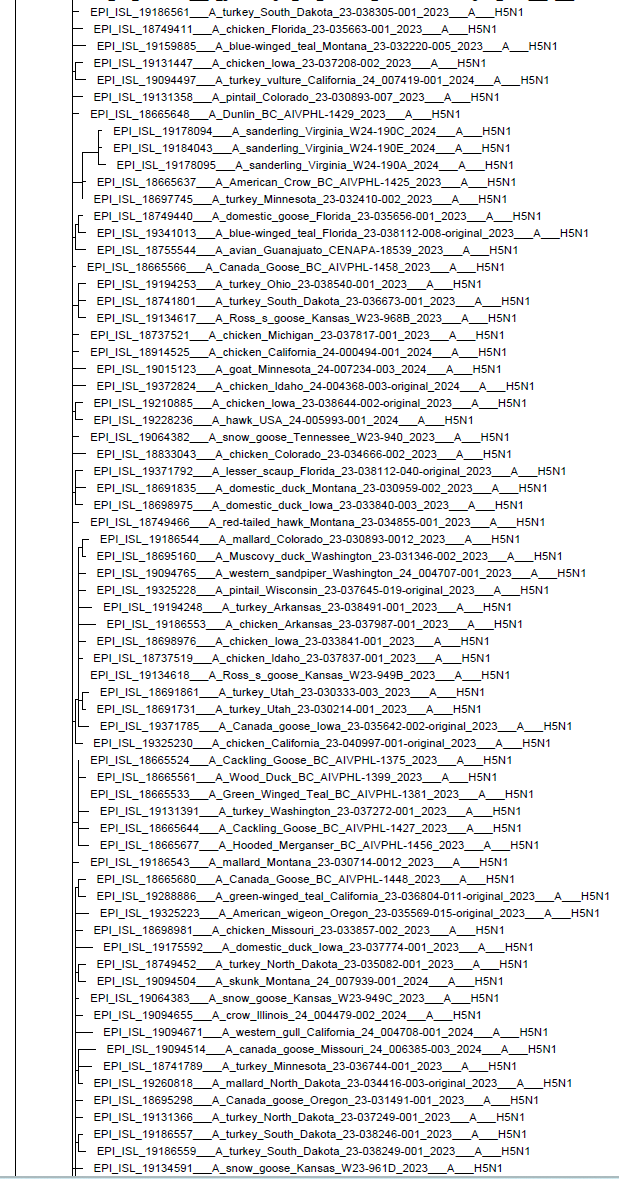


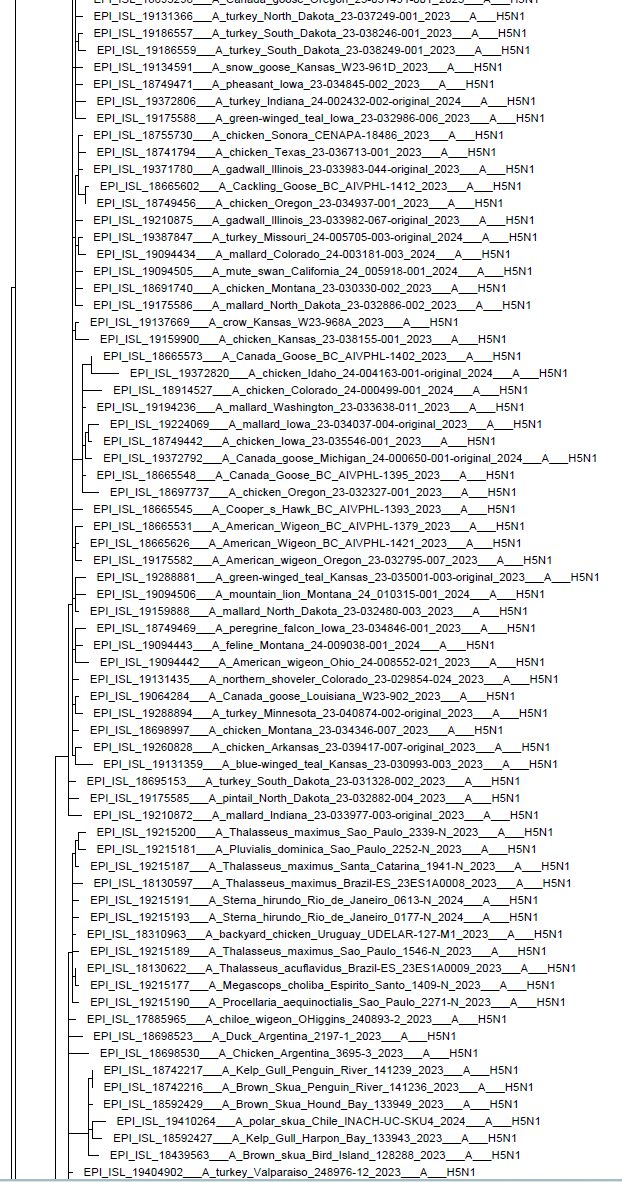


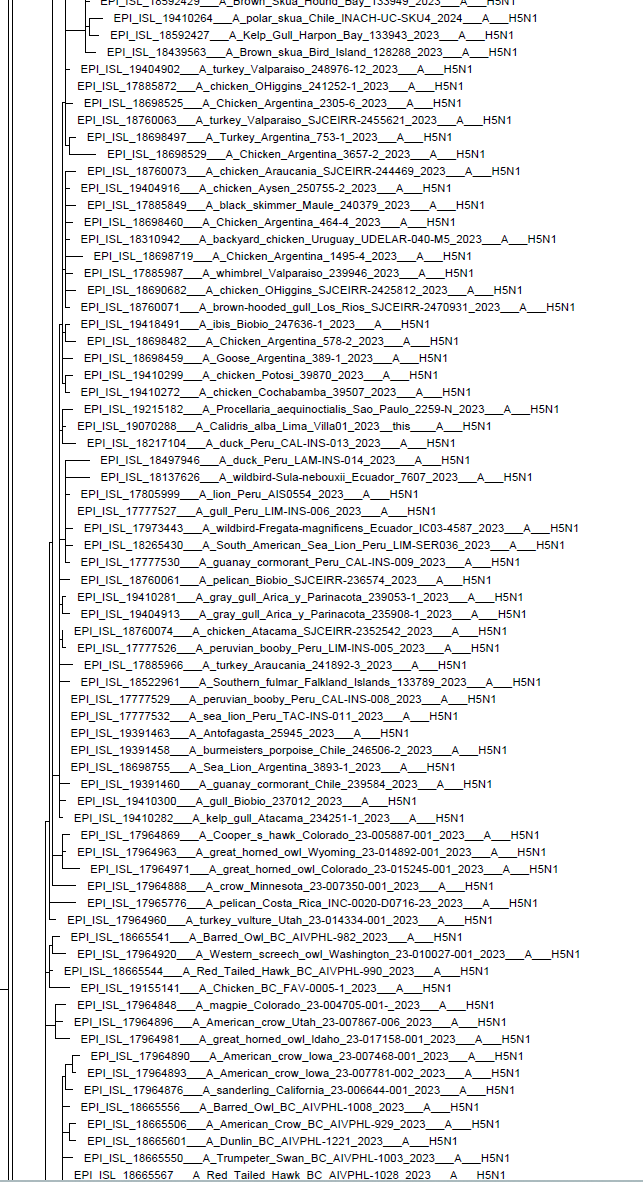


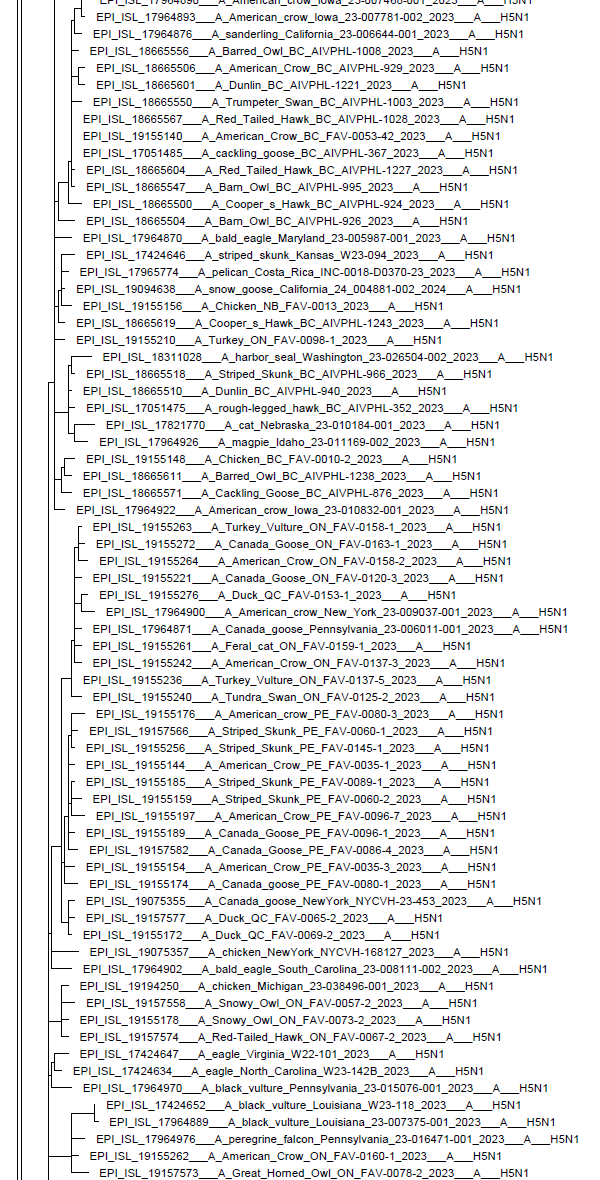


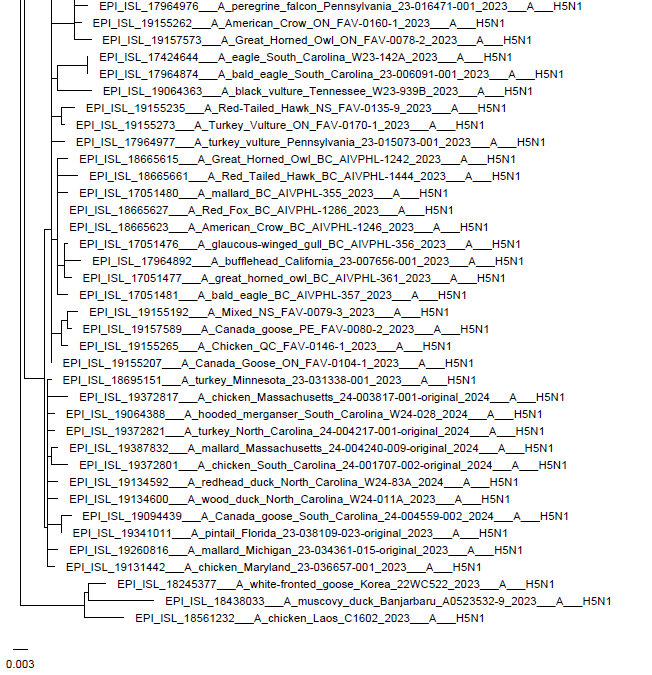

Supplement: Appendix Figure 2.docx [file TEMI_A_2505649_SM3534.docx]

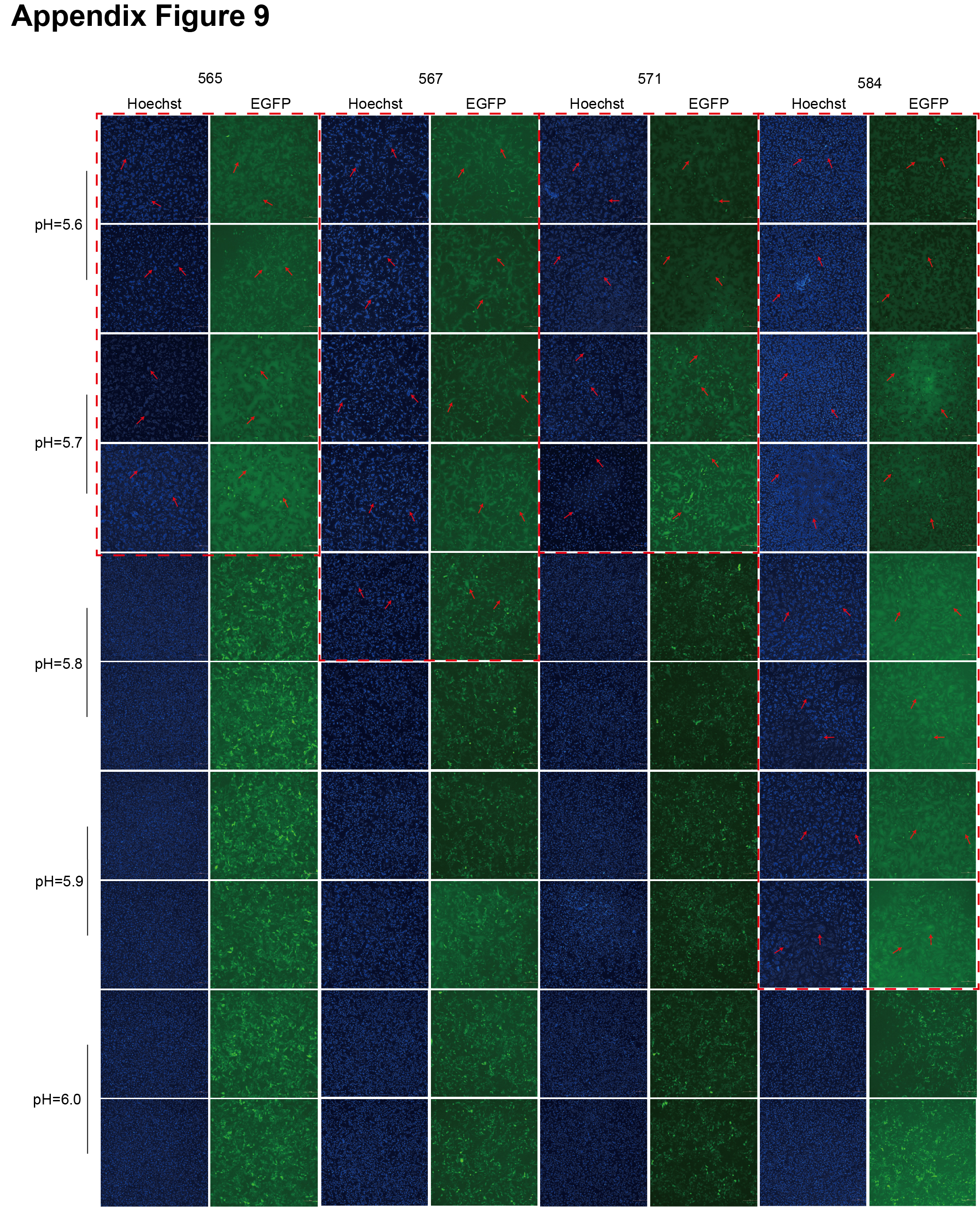

Supplement: Appendix Figure 9.jpg [file TEMI_A_2505649_SM3531.jpg]

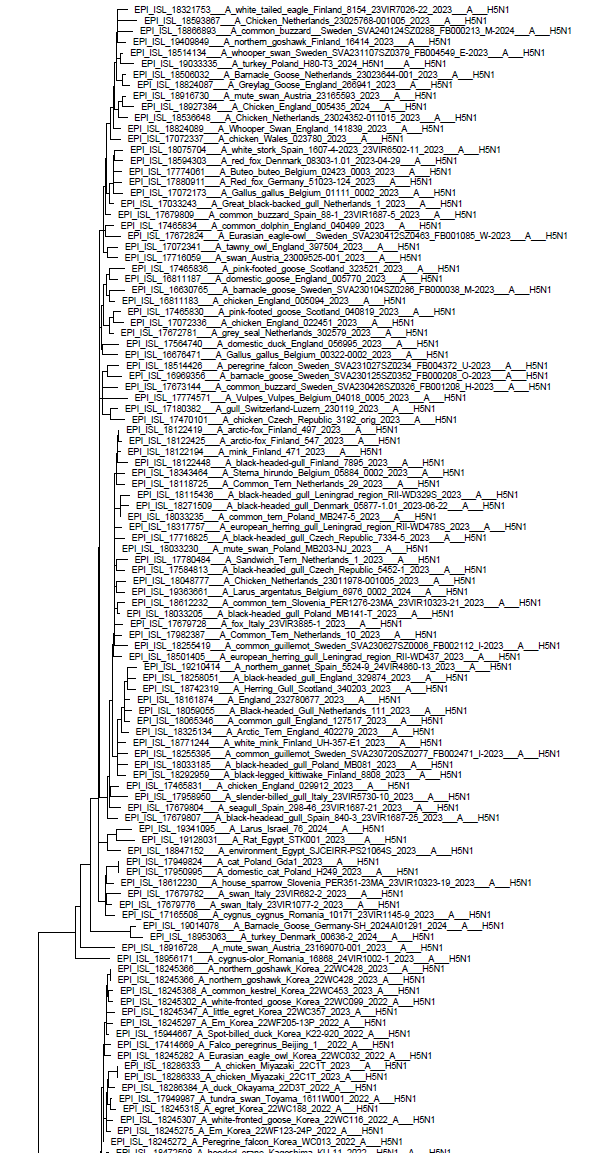


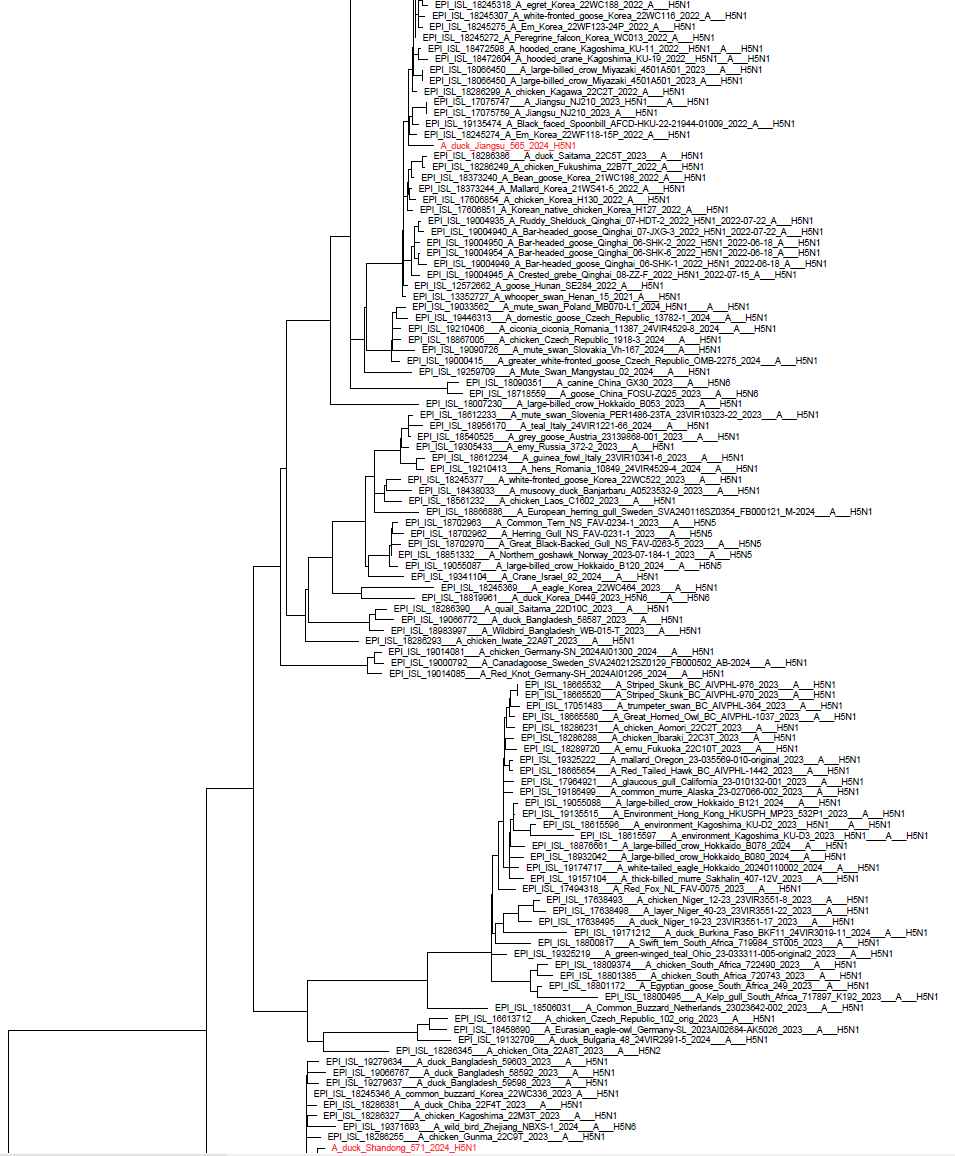


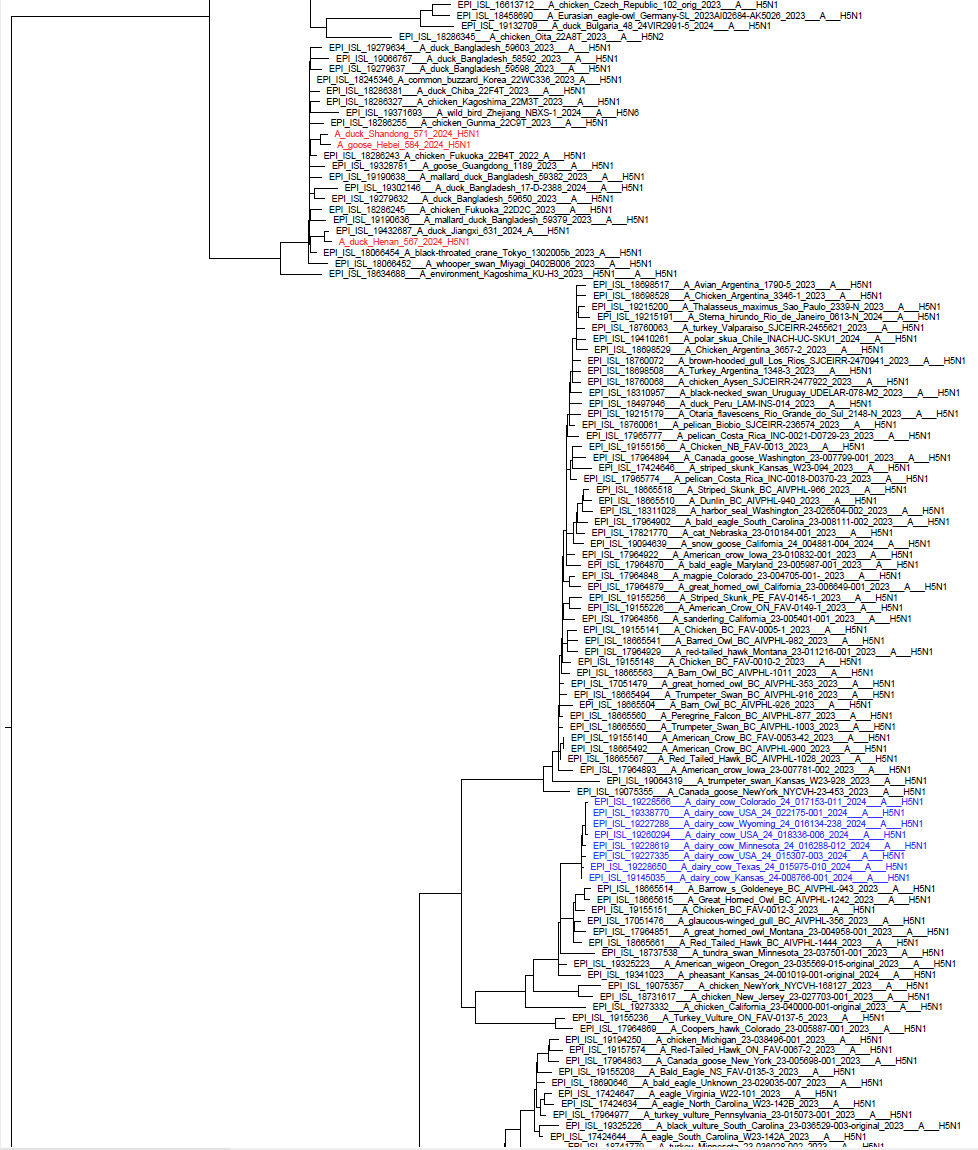


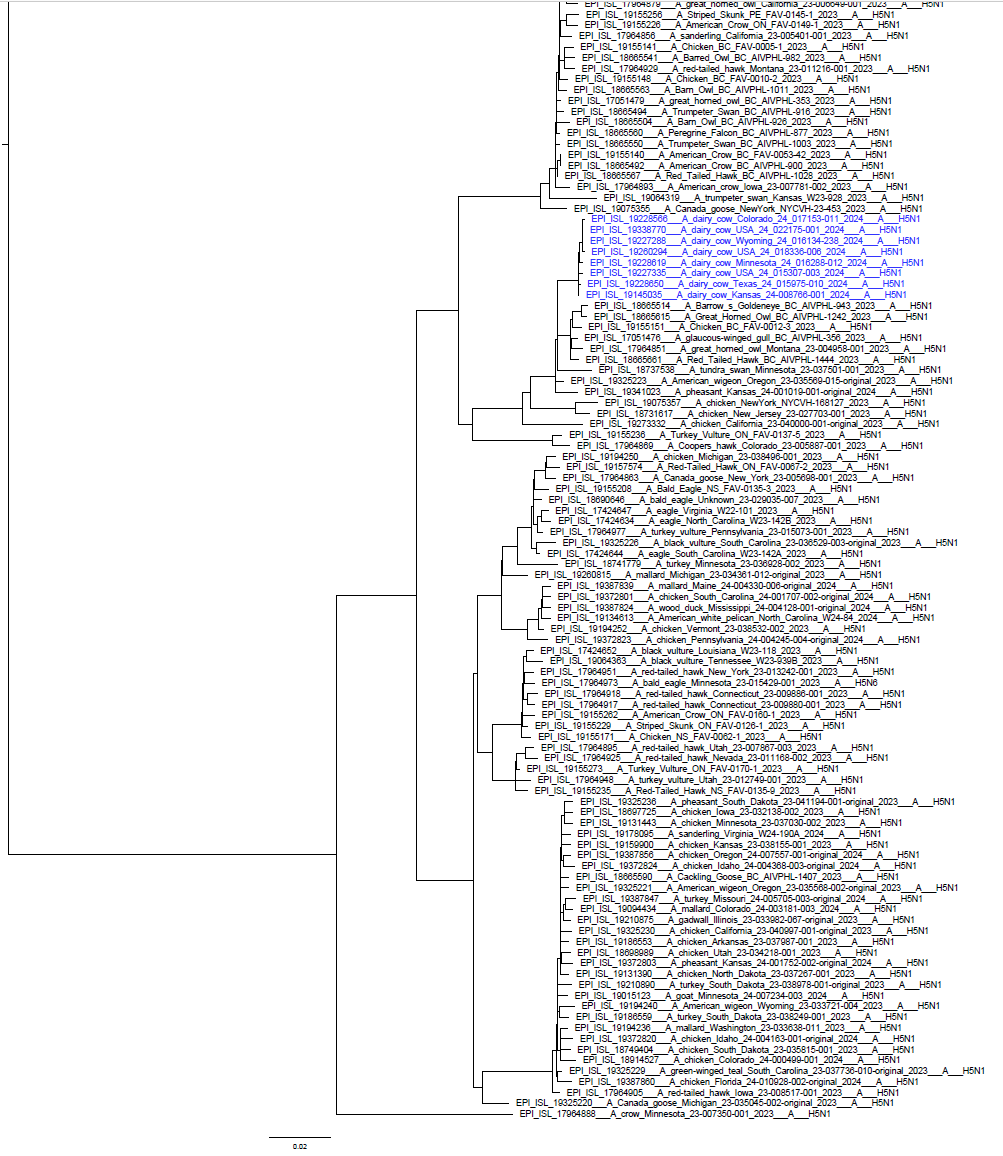

Supplement: Appendix Figure 3.docx [file TEMI_A_2505649_SM3530.docx]

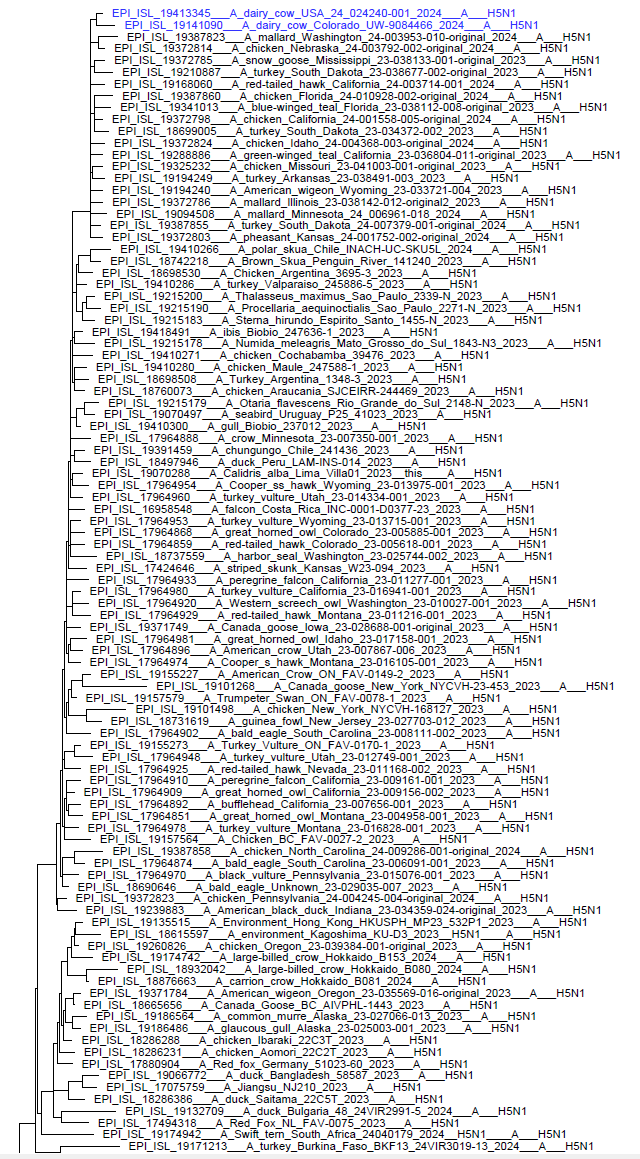


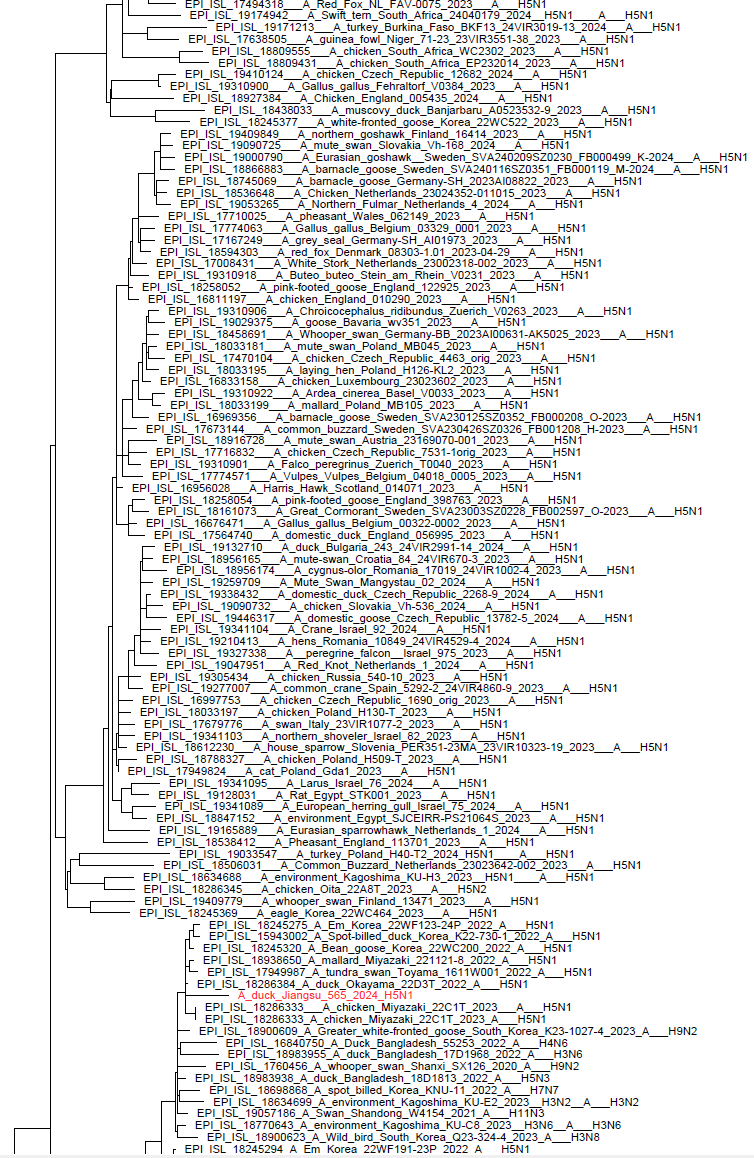


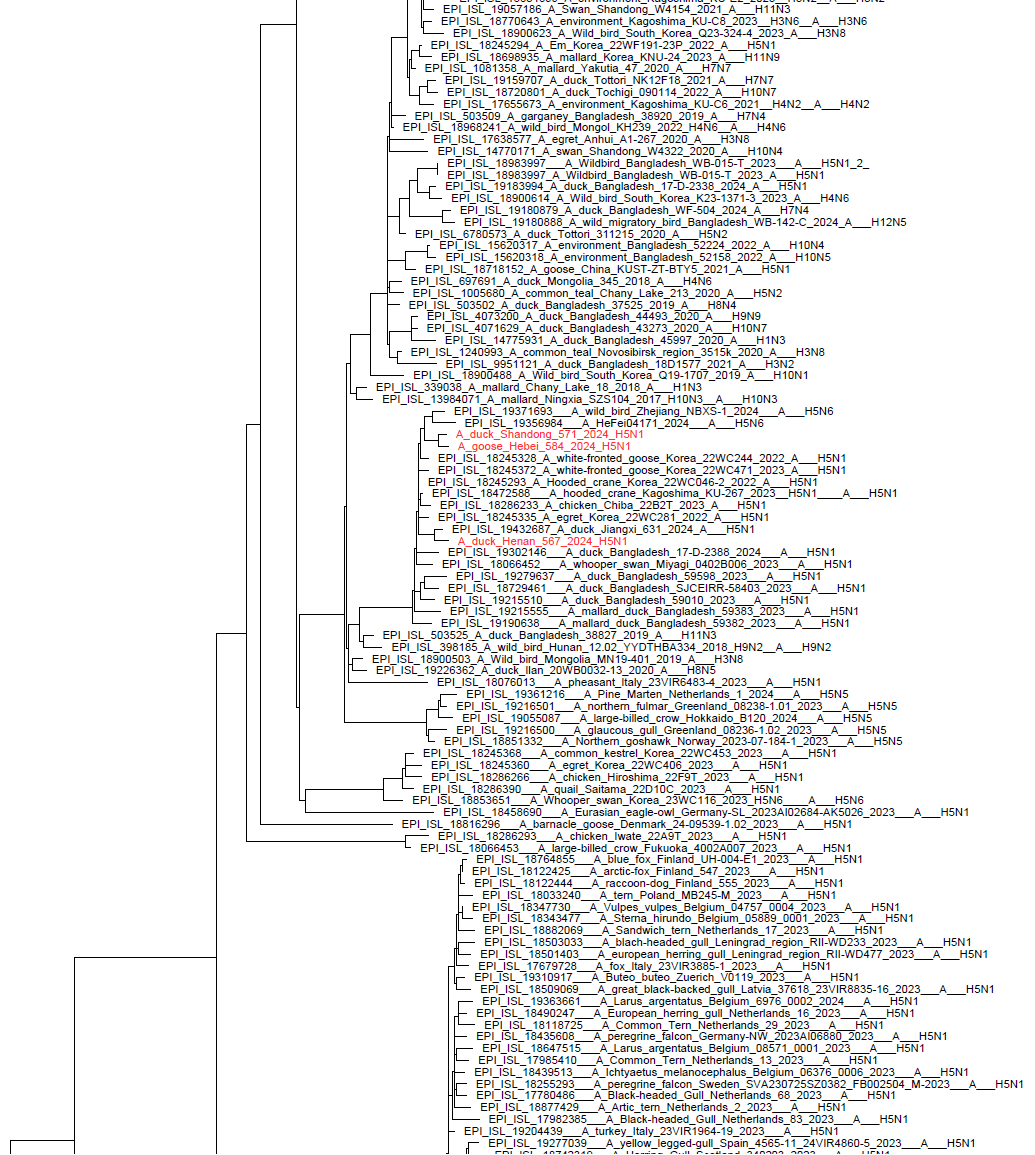


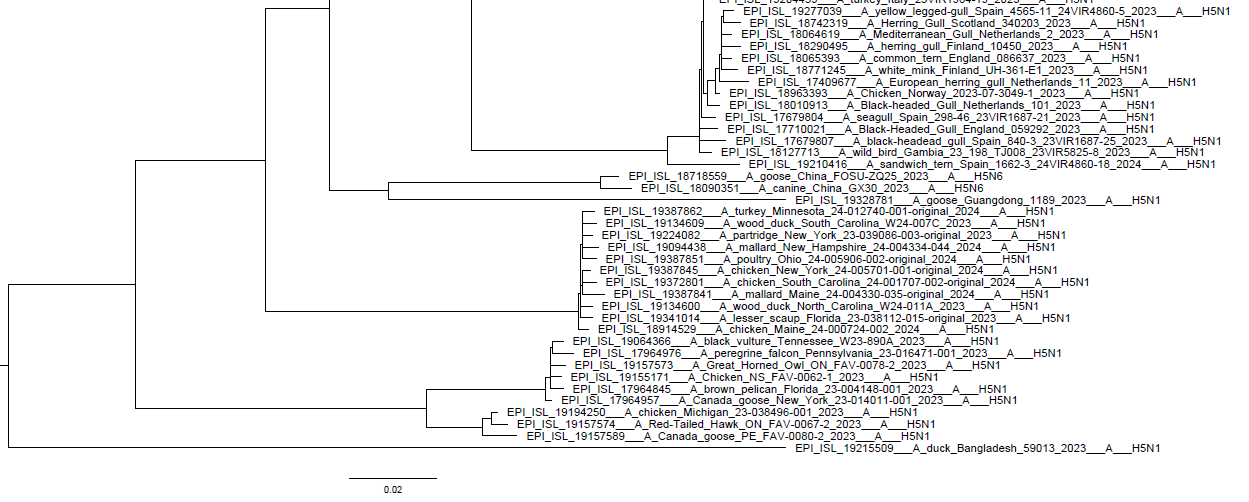

Supplement: Appendix Figure 5.docx [file TEMI_A_2505649_SM3529.docx]

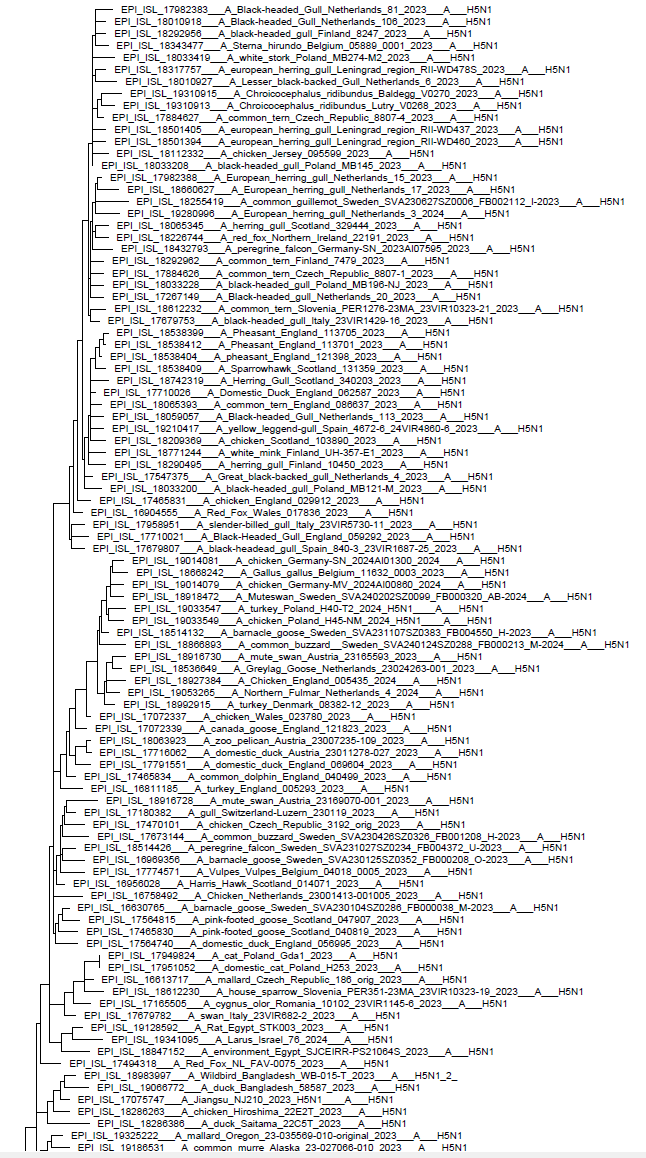


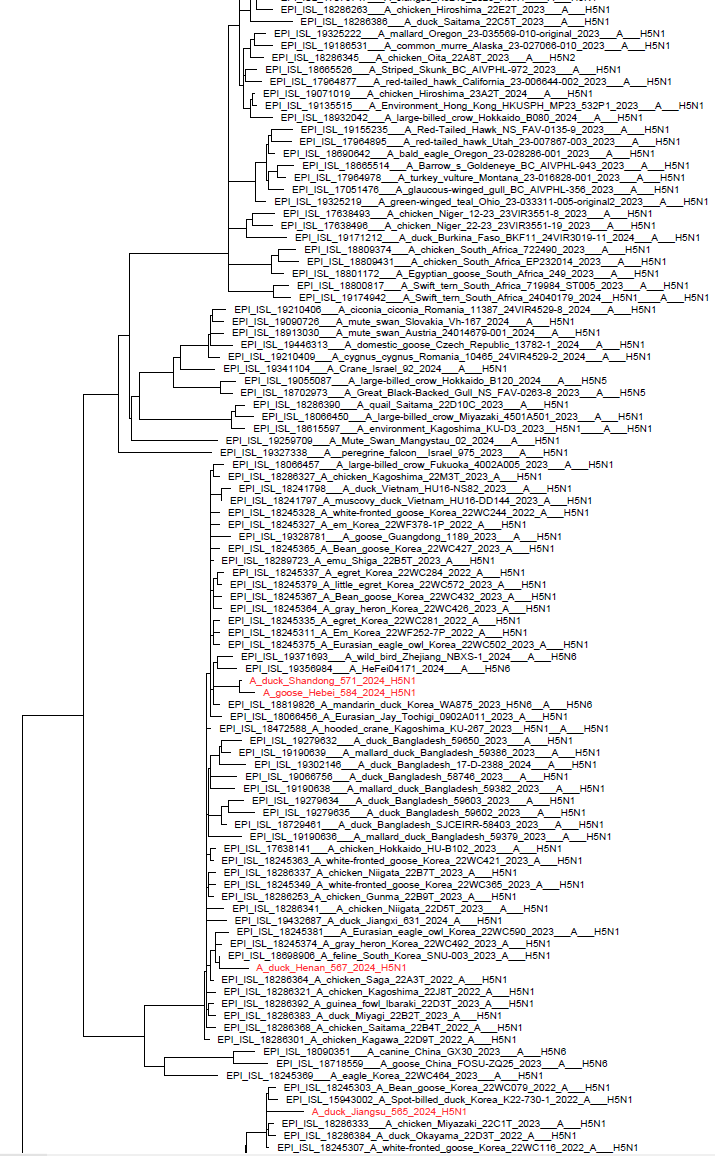


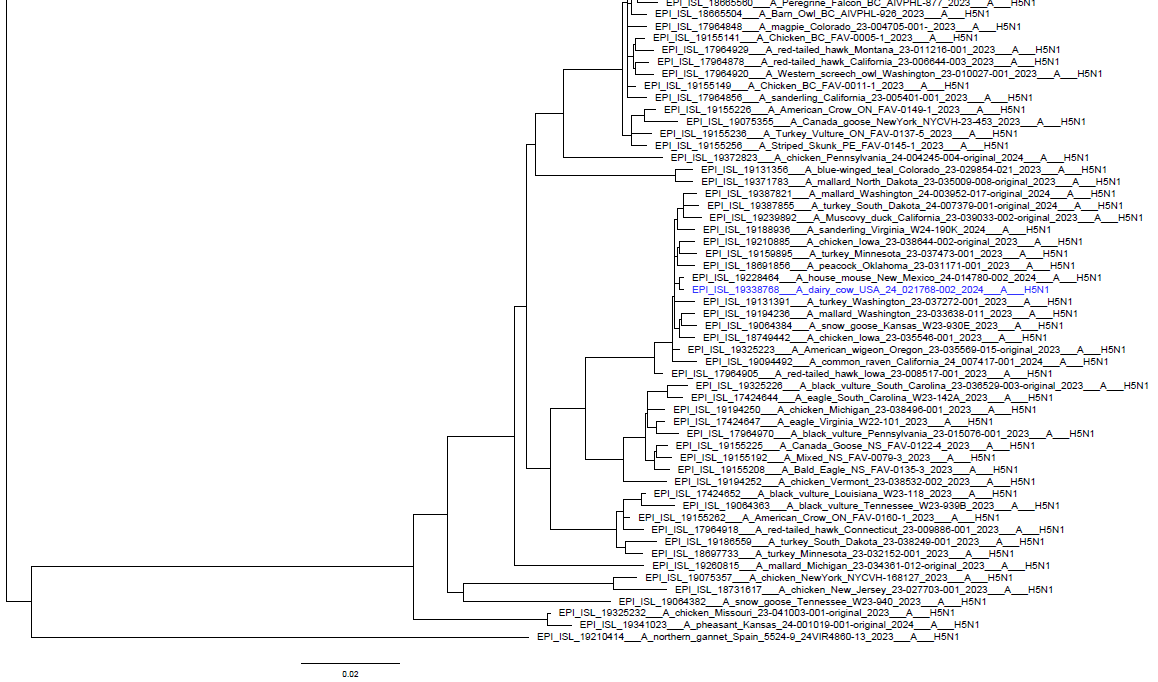

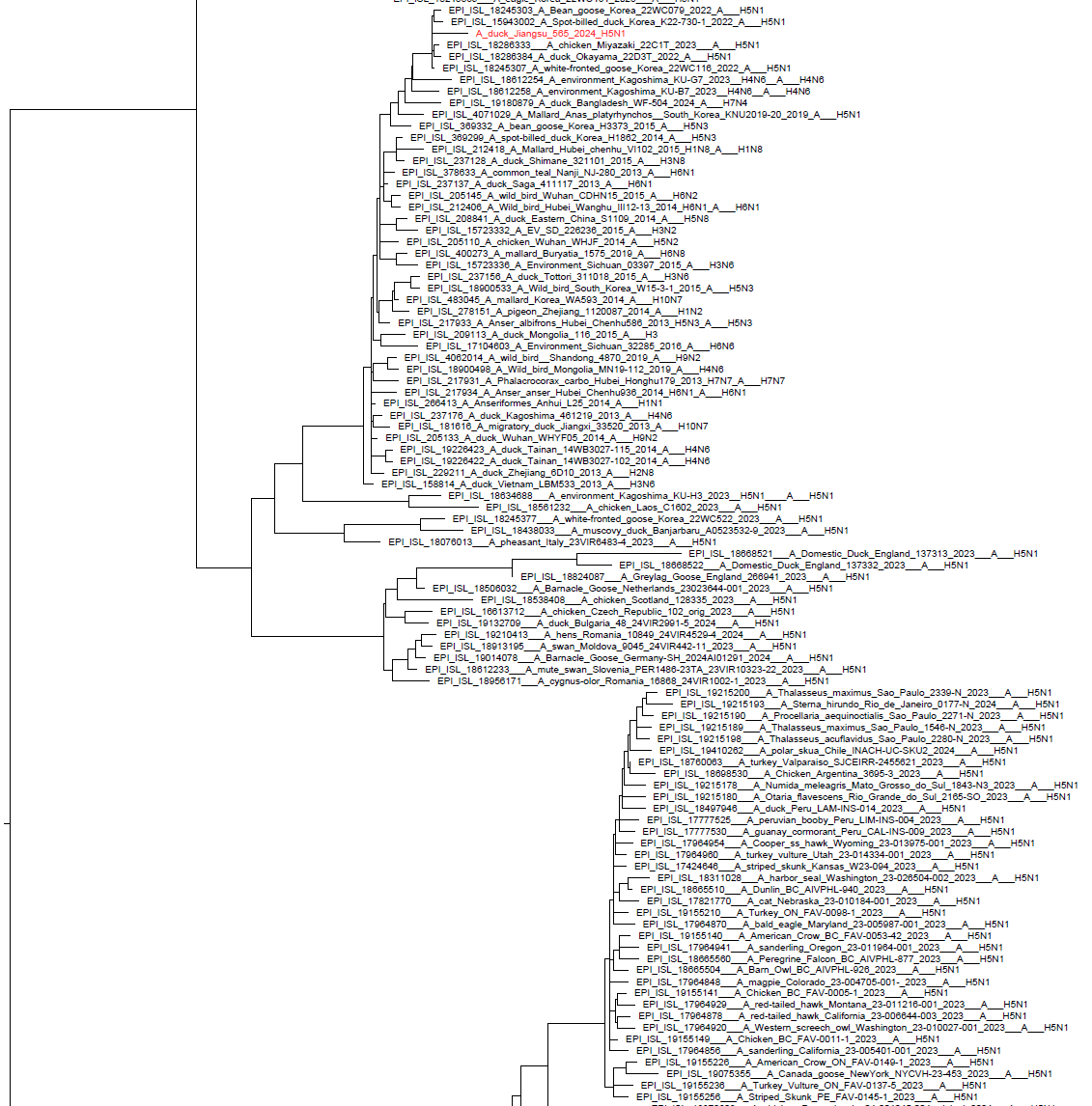

Supplement: Appendix Figure 4.docx [file TEMI_A_2505649_SM3528.docx]

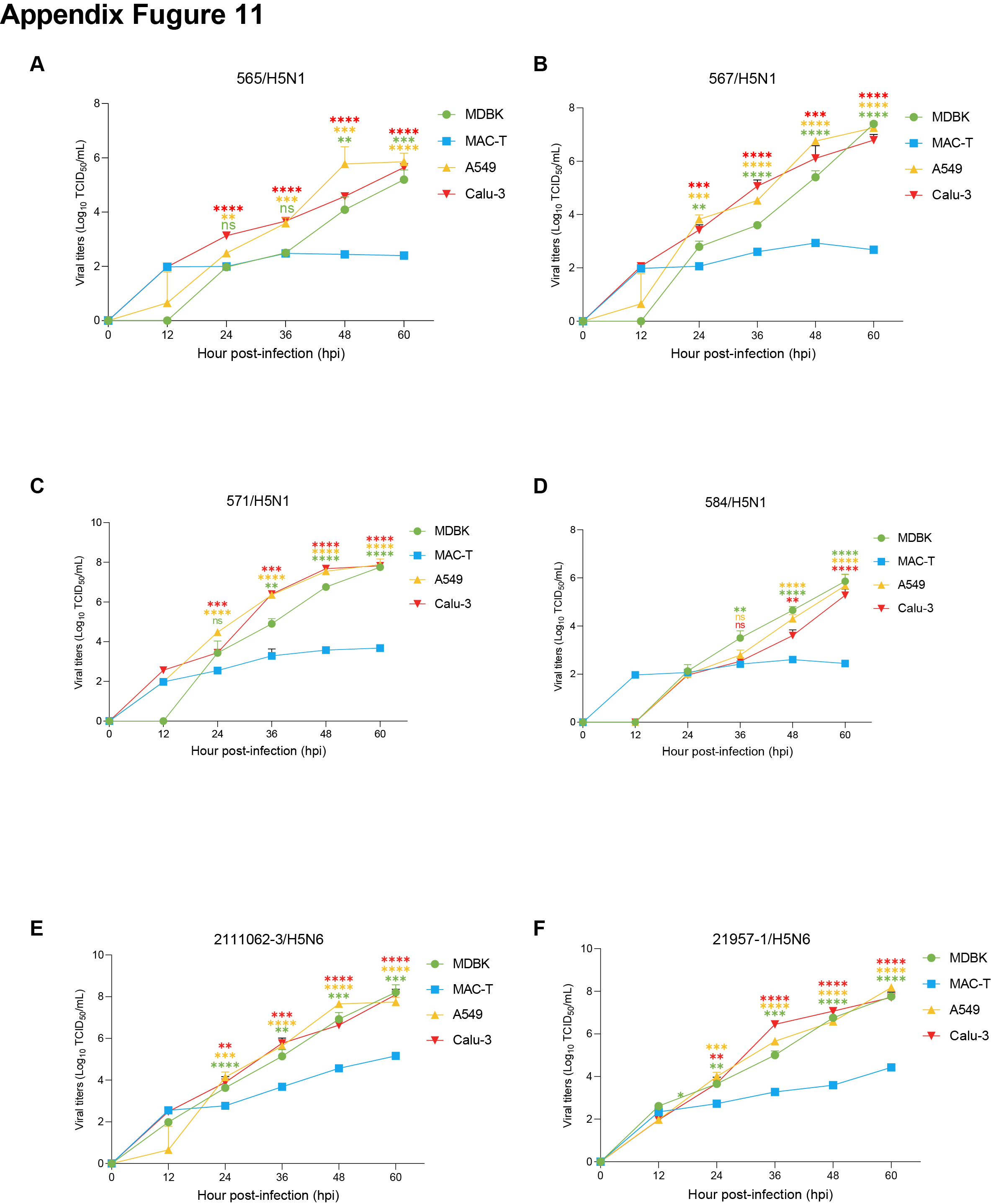

Supplement: Appendix Figure 11.jpg [file TEMI_A_2505649_SM3525.jpg]

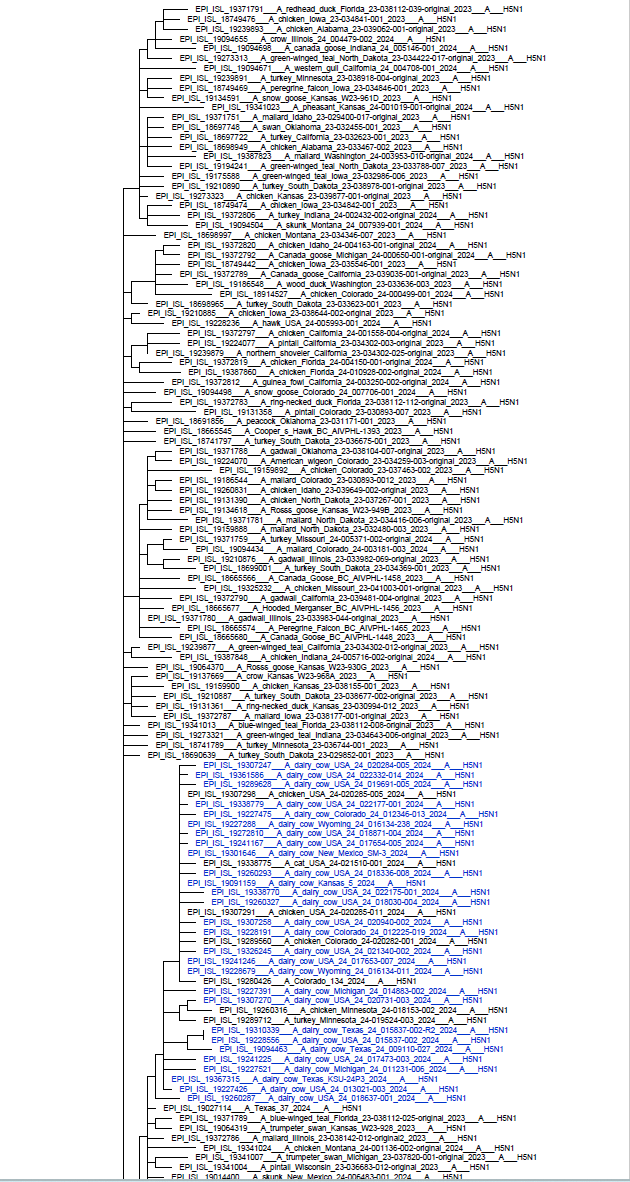


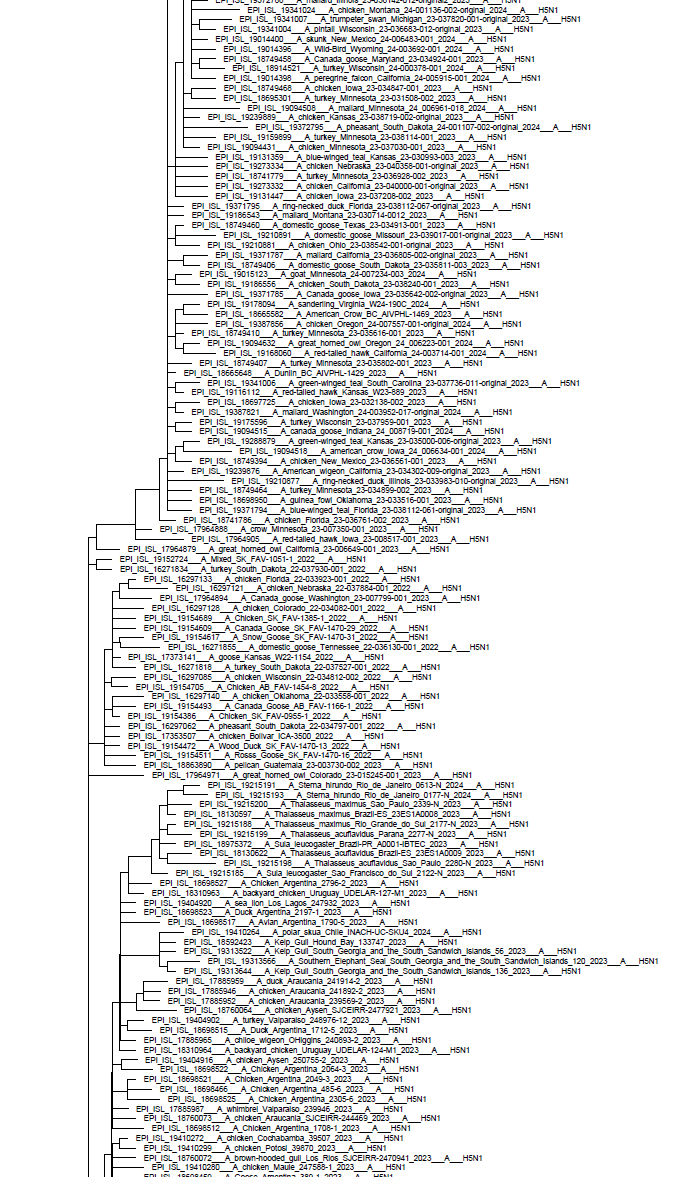


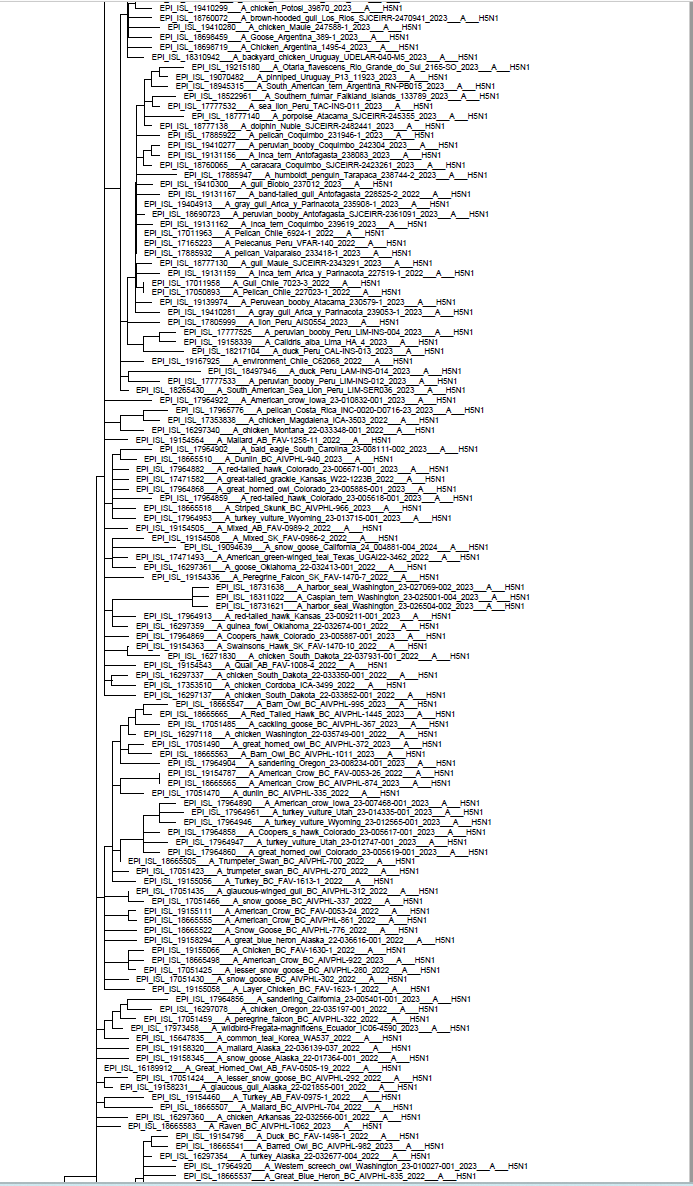


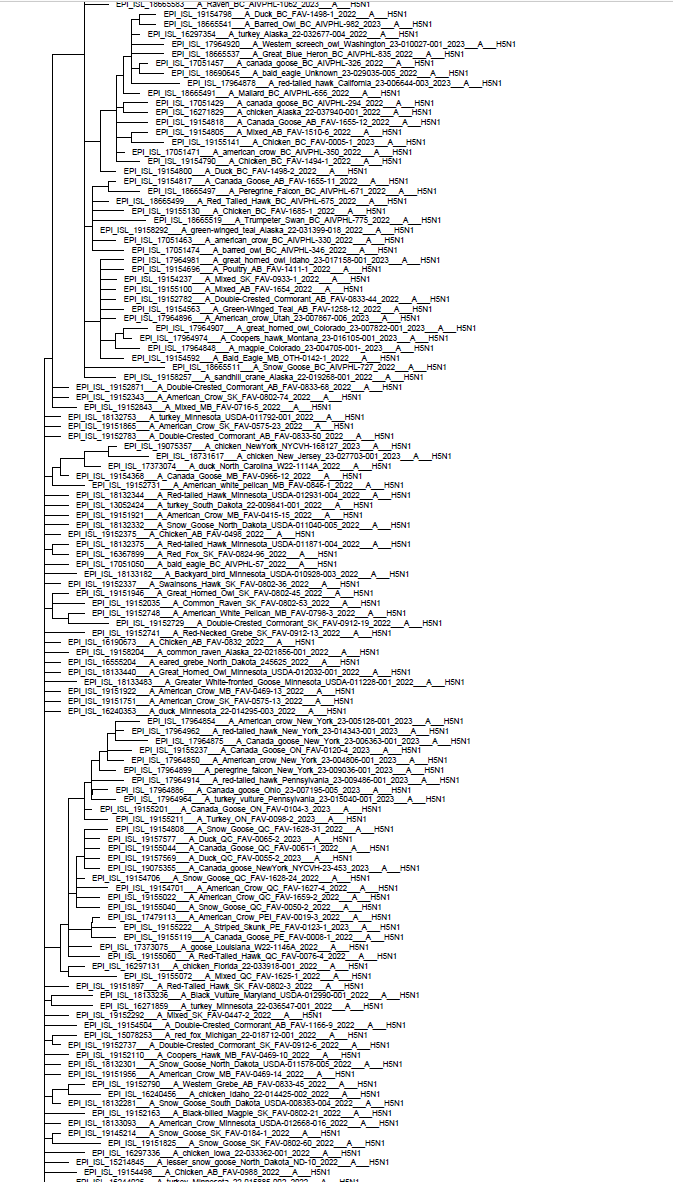


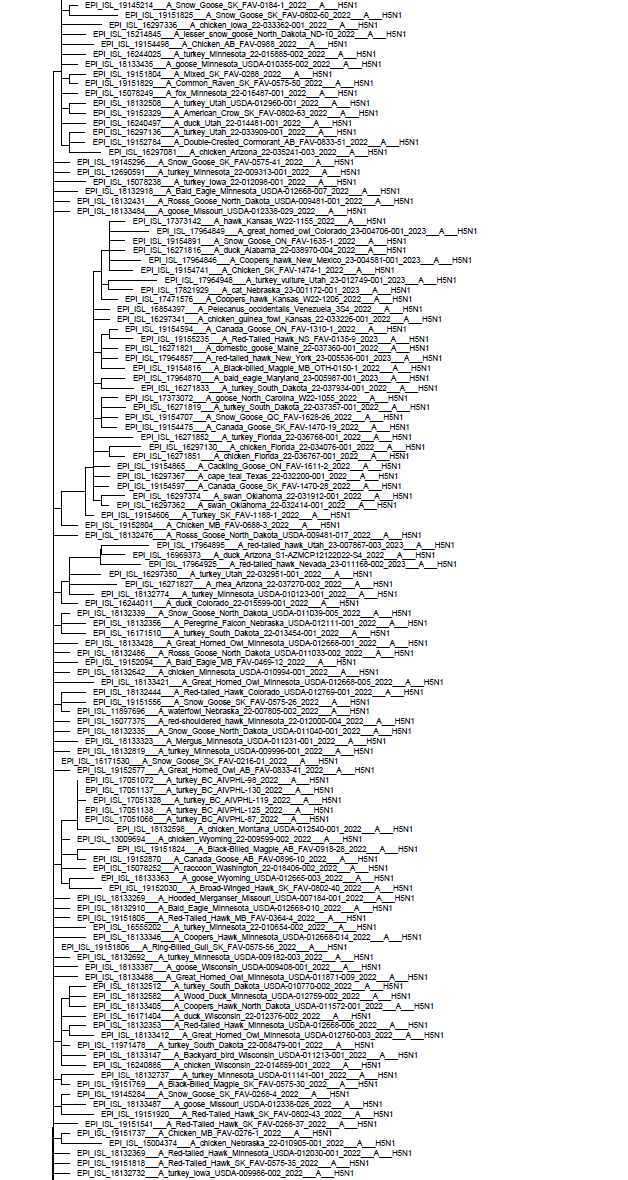


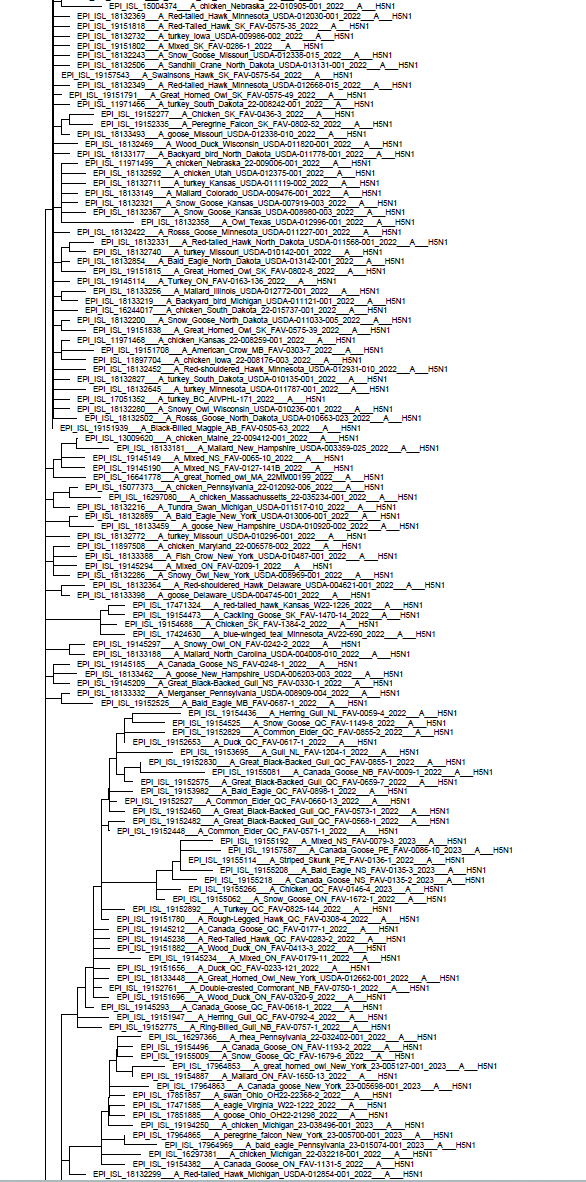


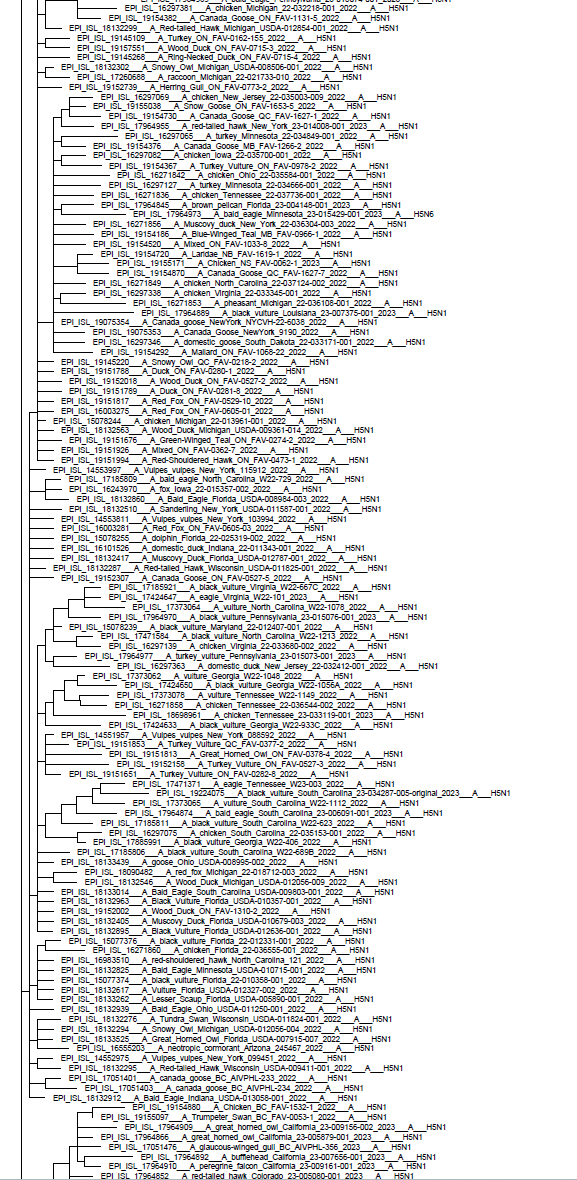


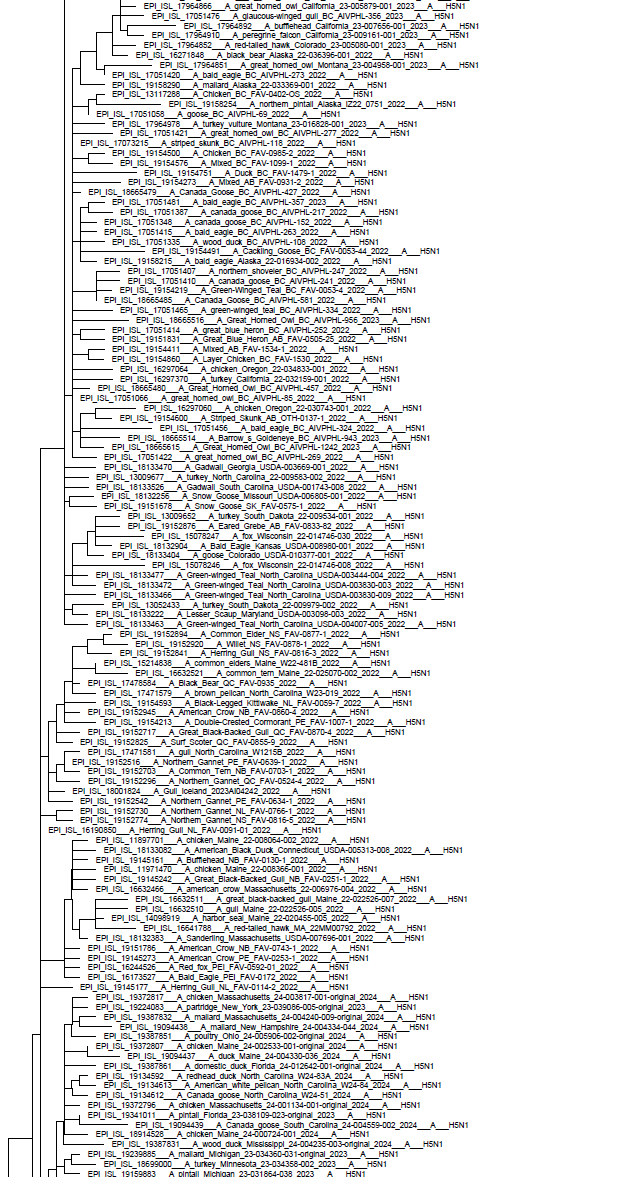


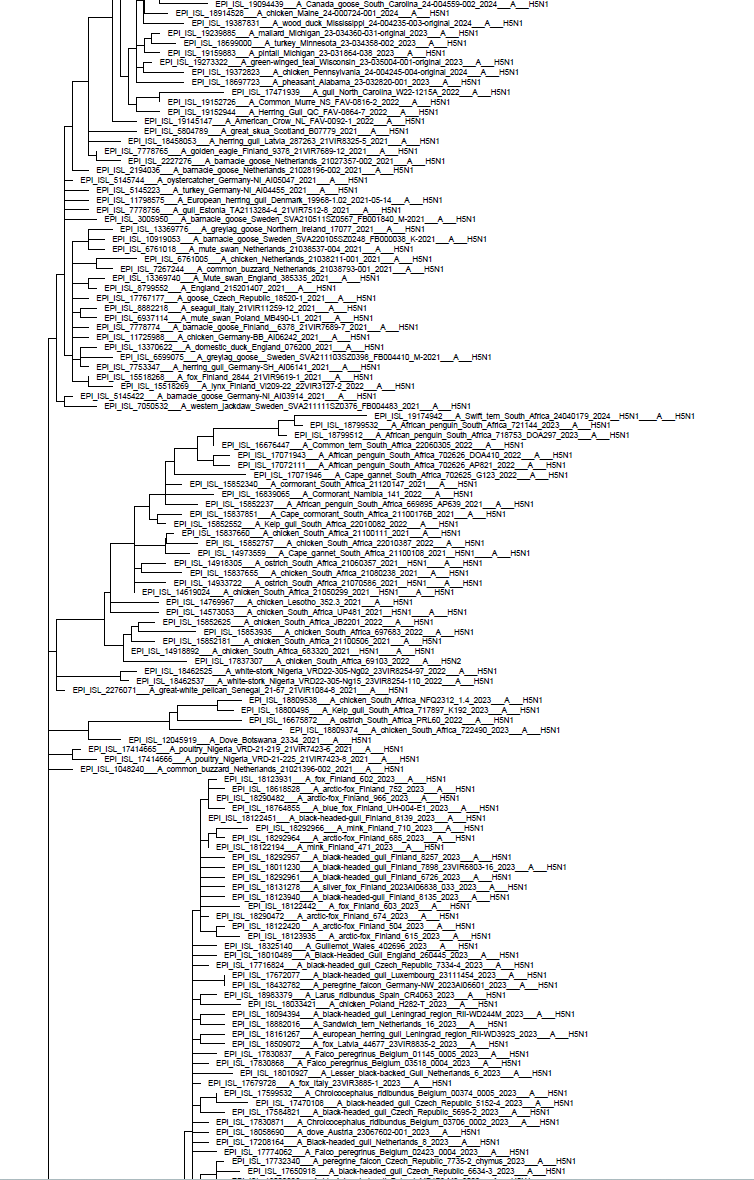


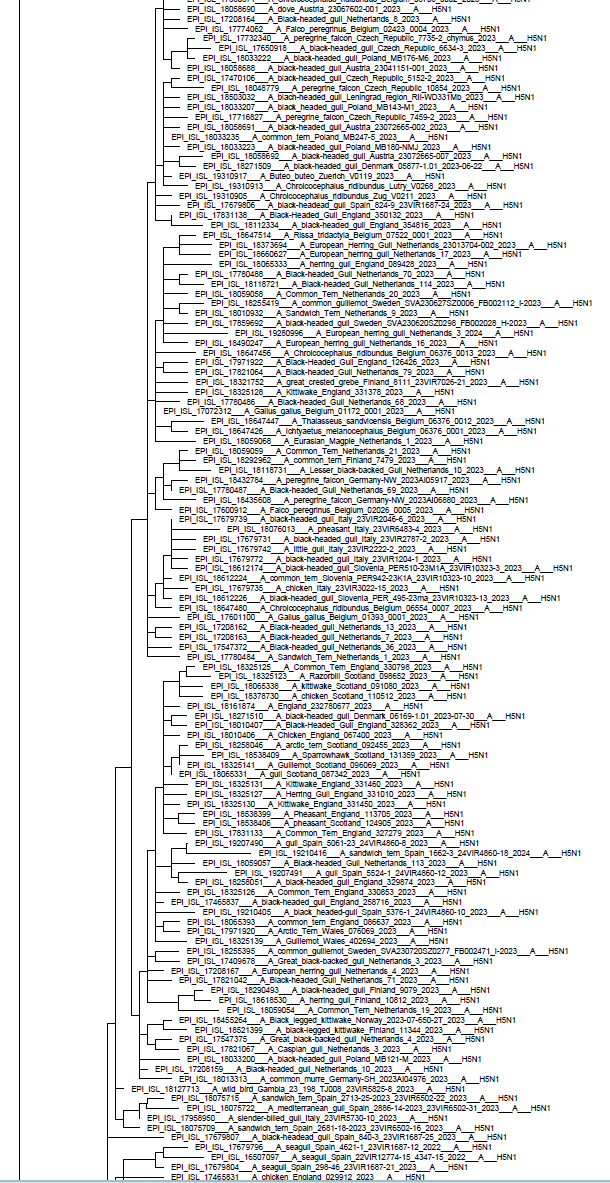


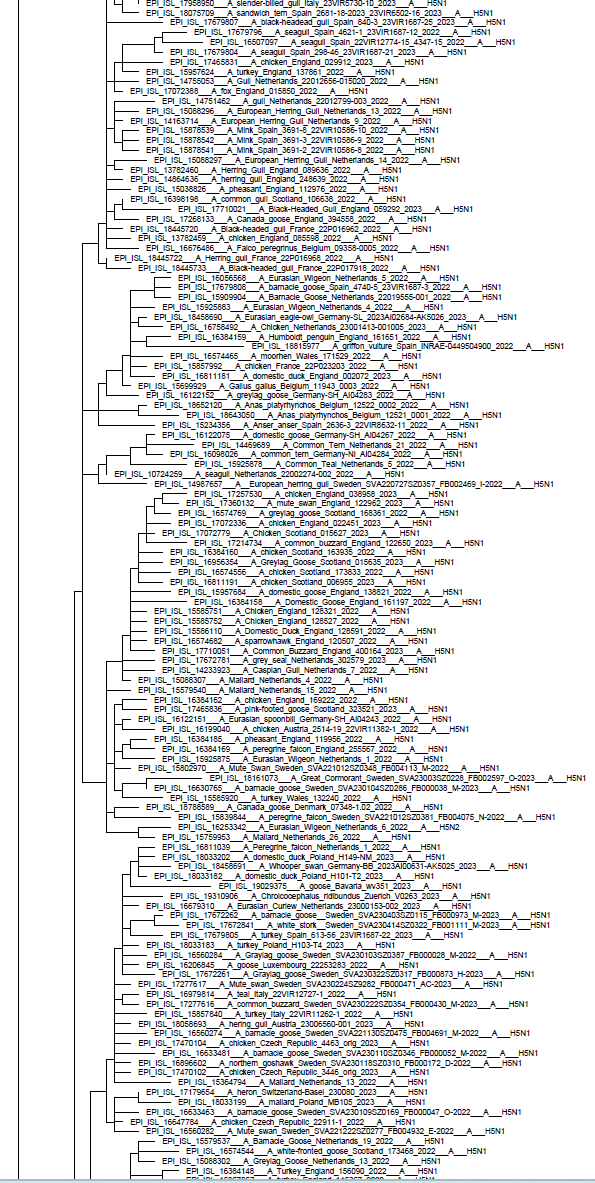


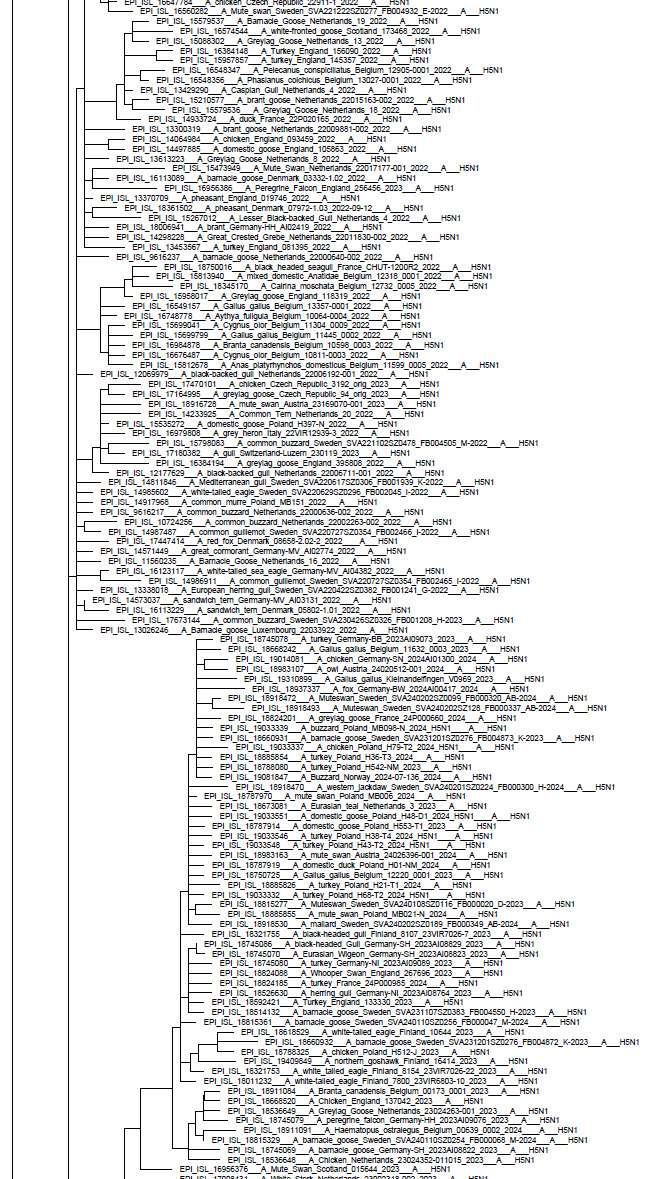


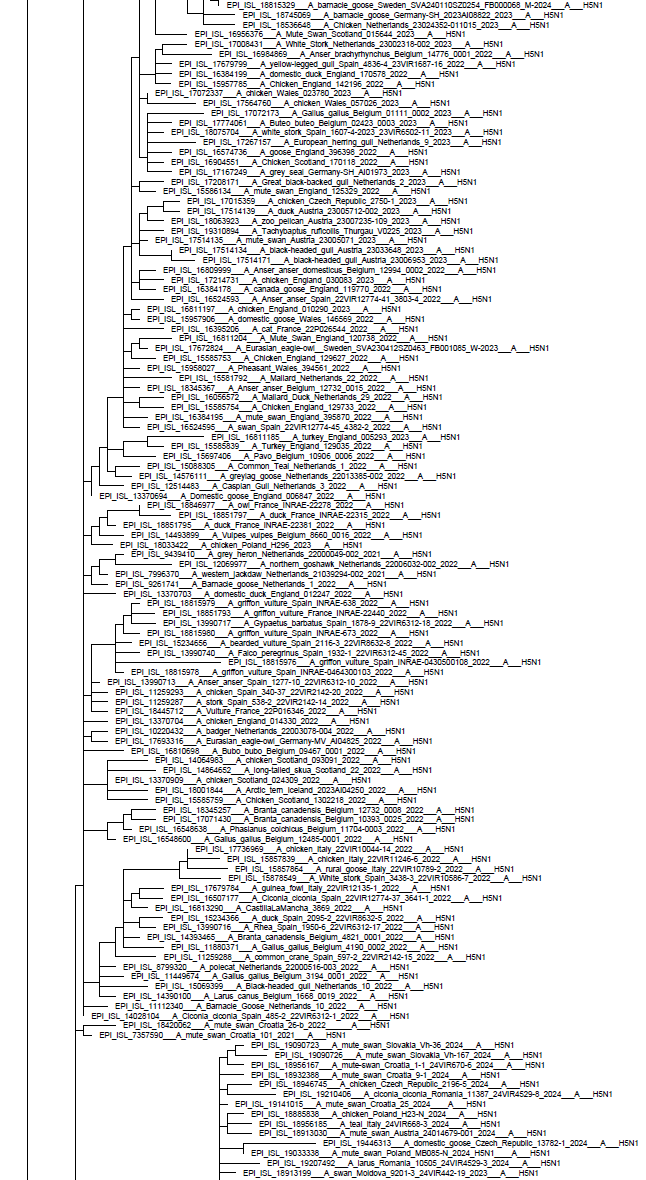


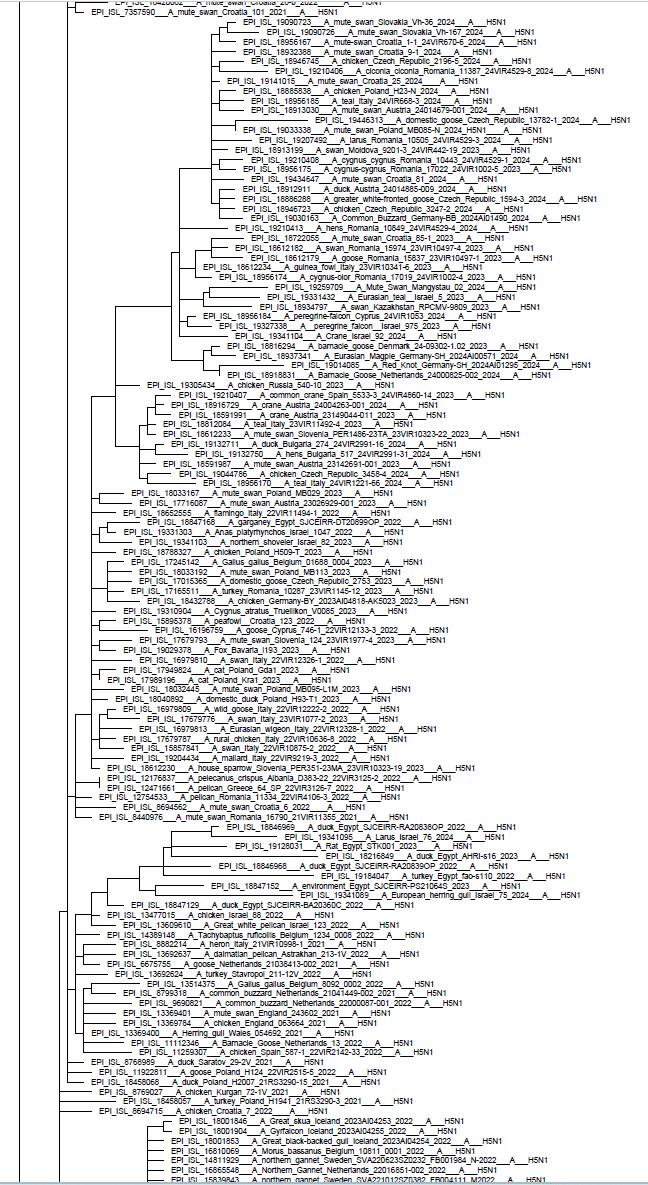


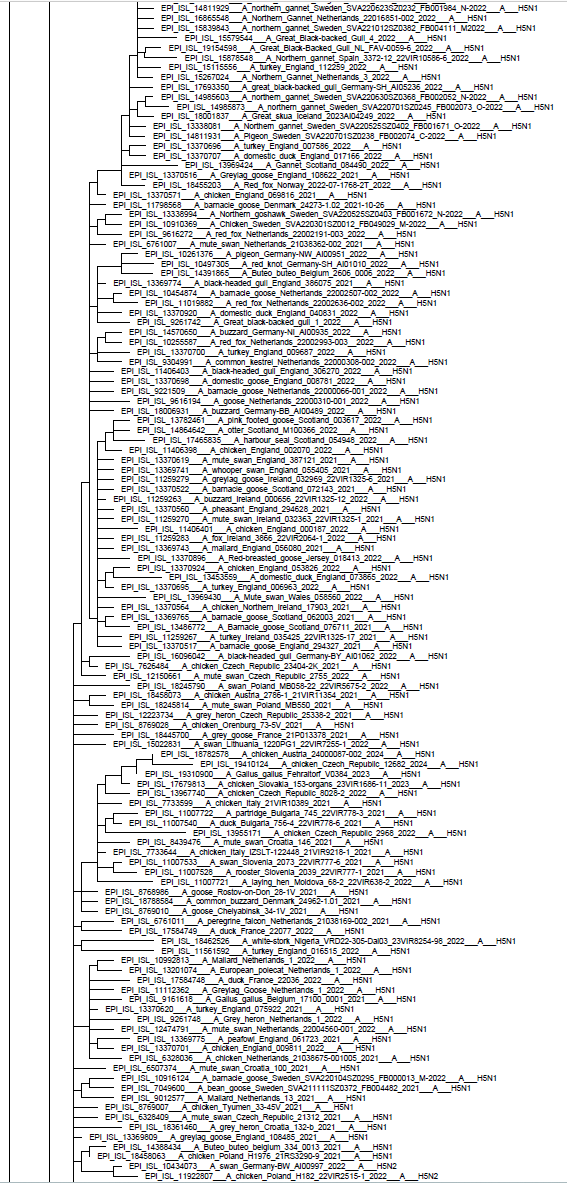

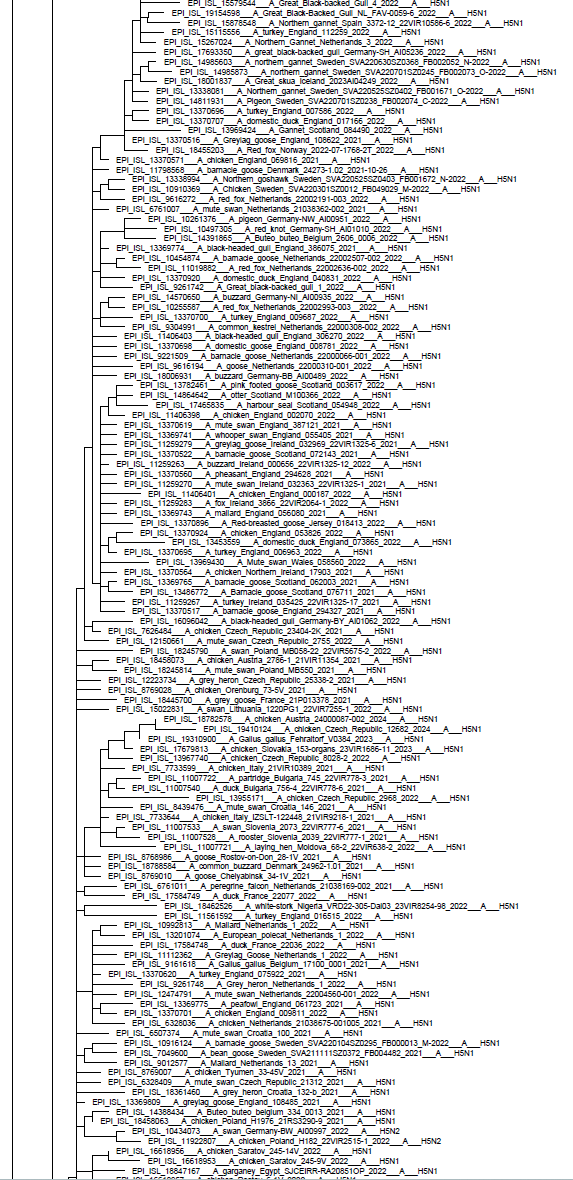


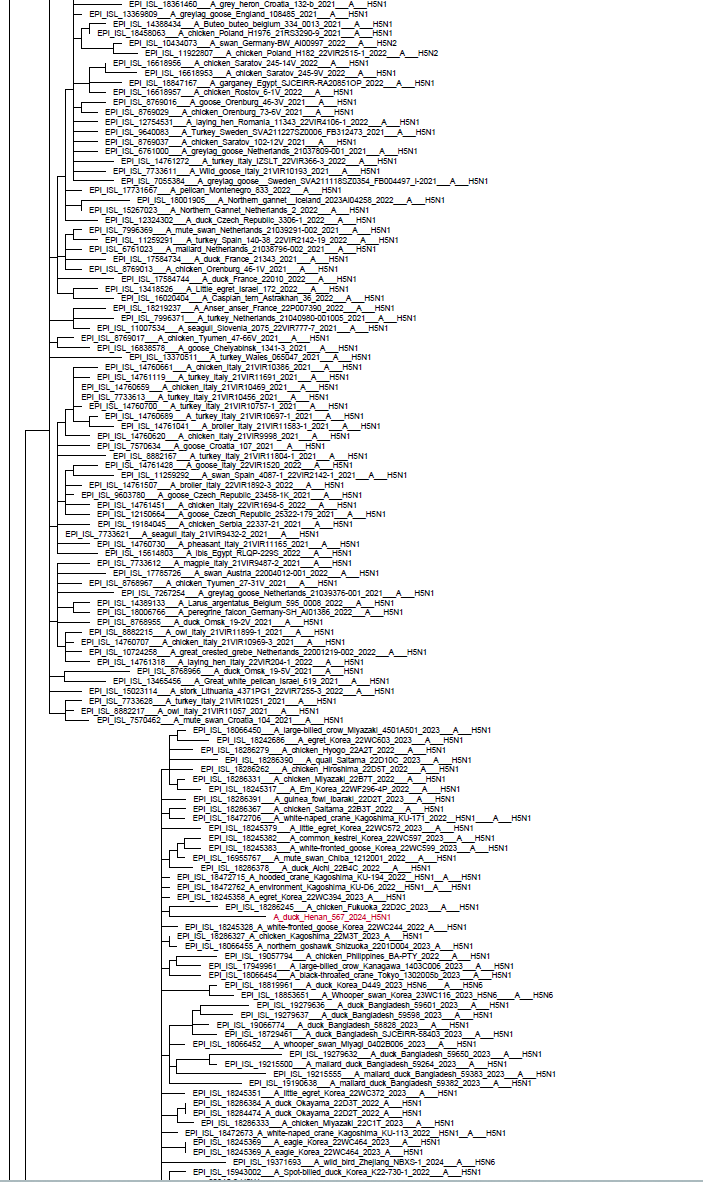


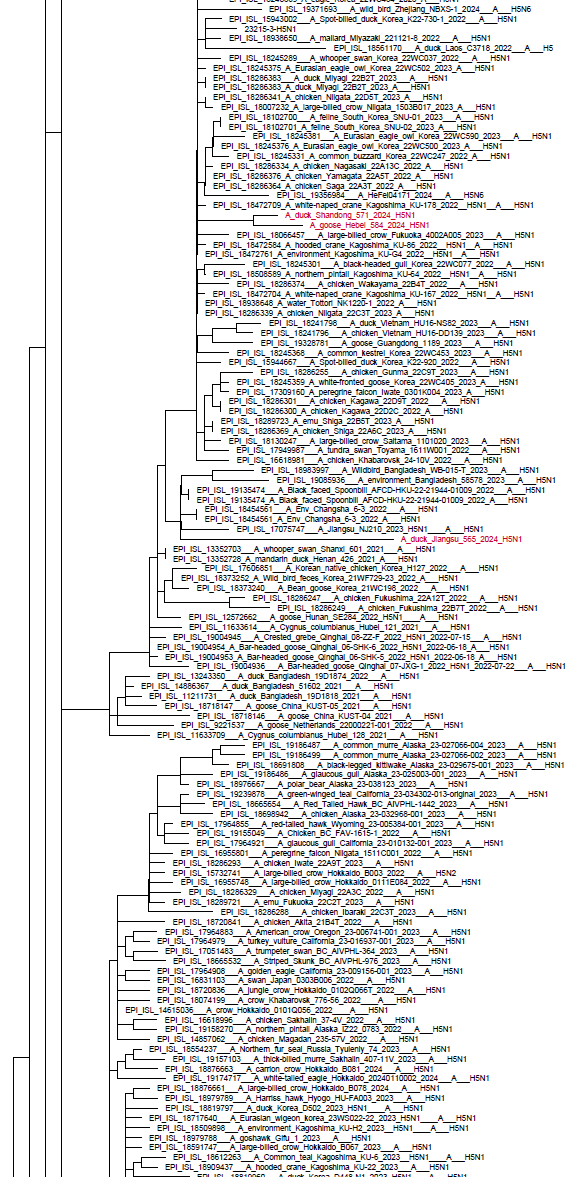


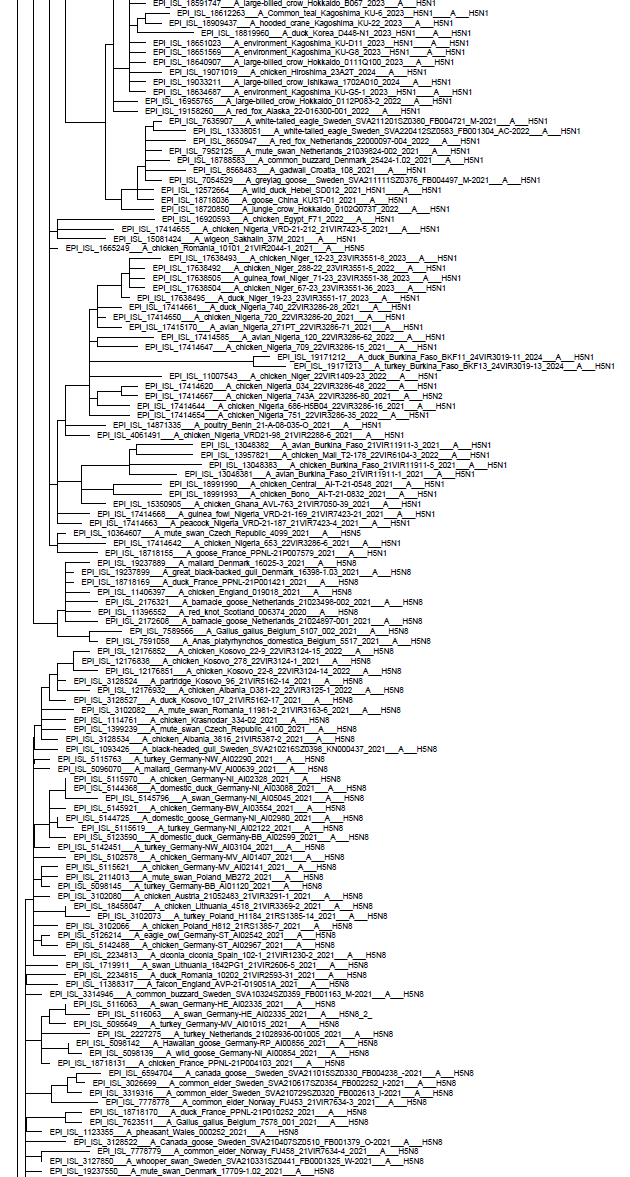


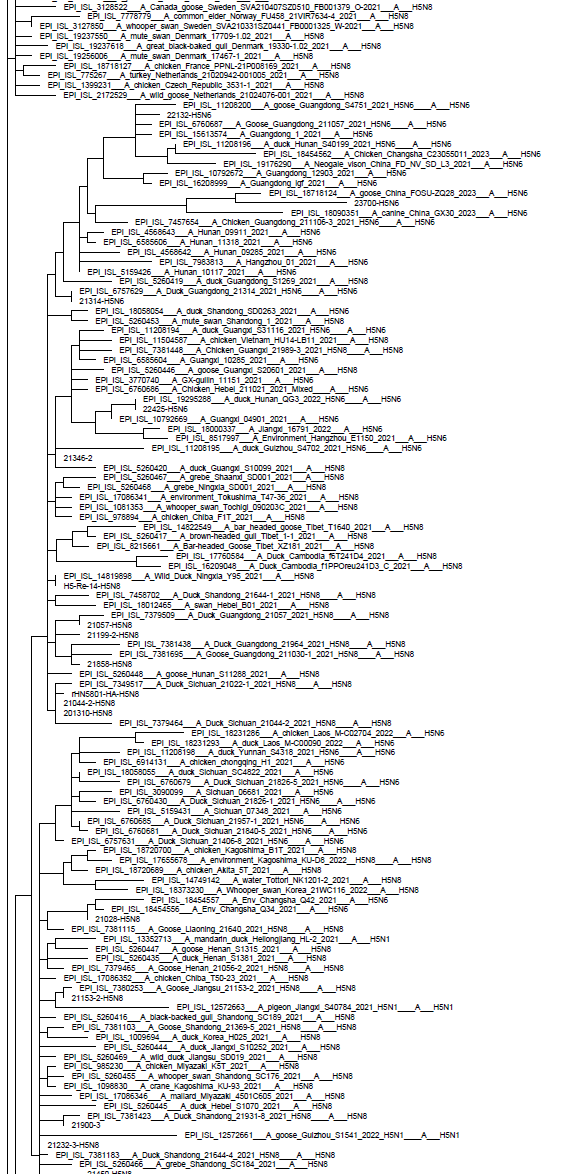


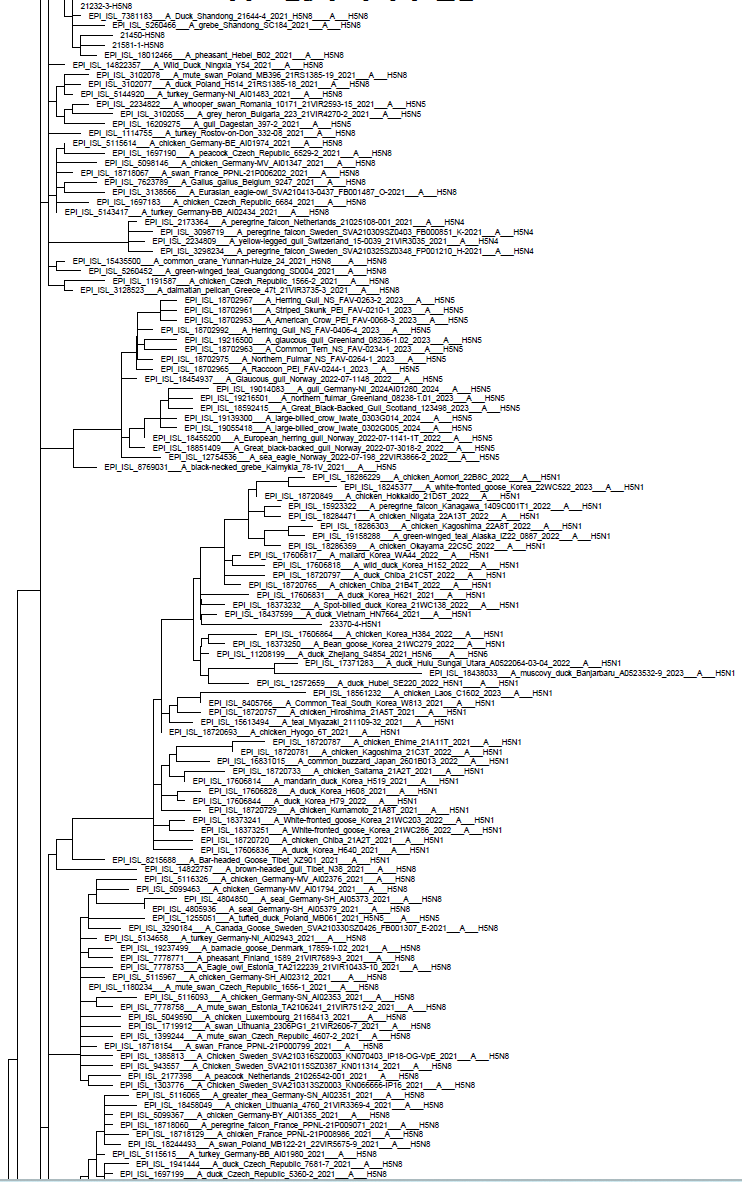


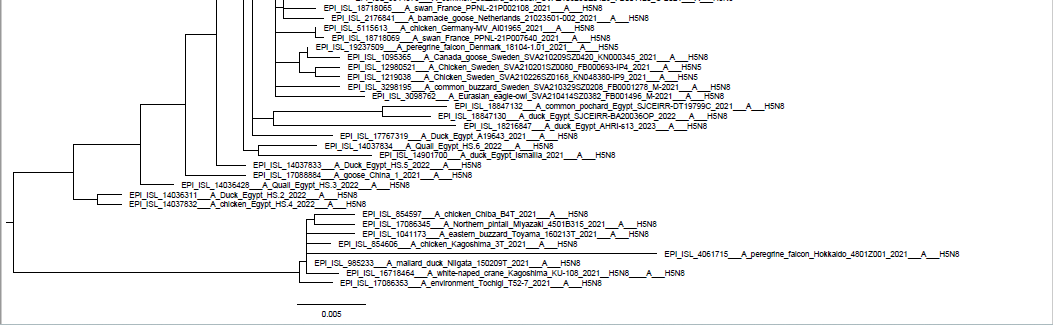

Supplement: Appendix Figure 1.docx [file TEMI_A_2505649_SM3524.docx]

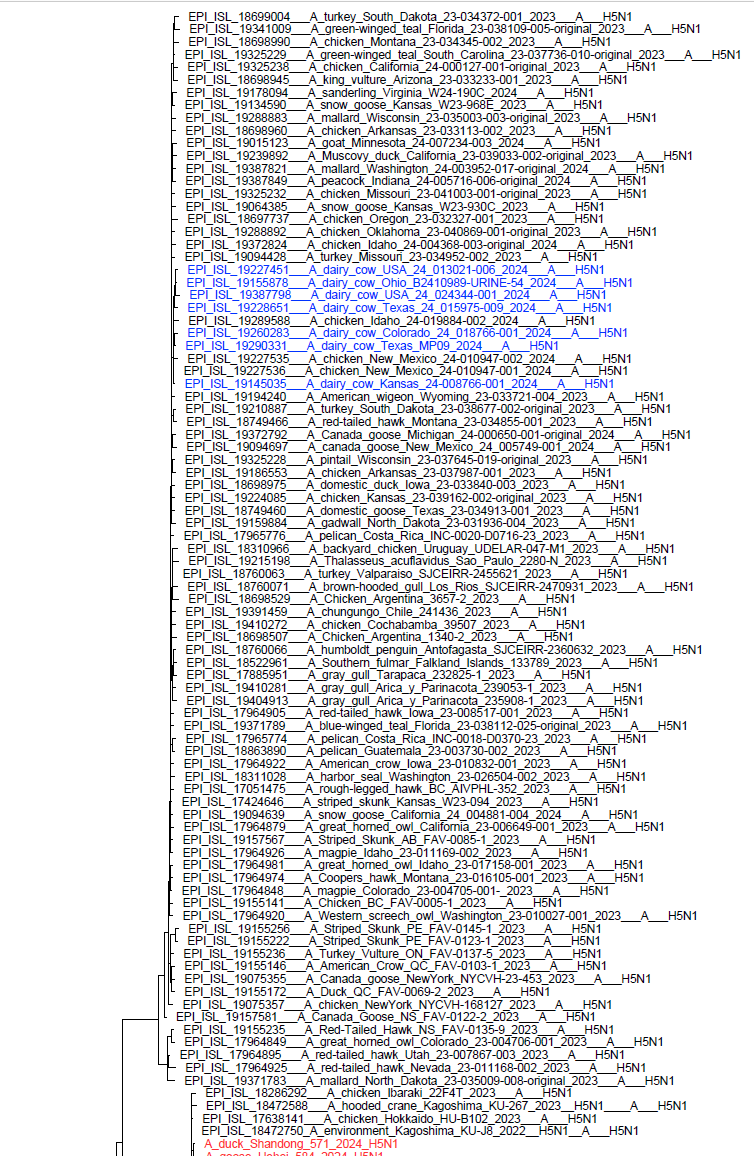


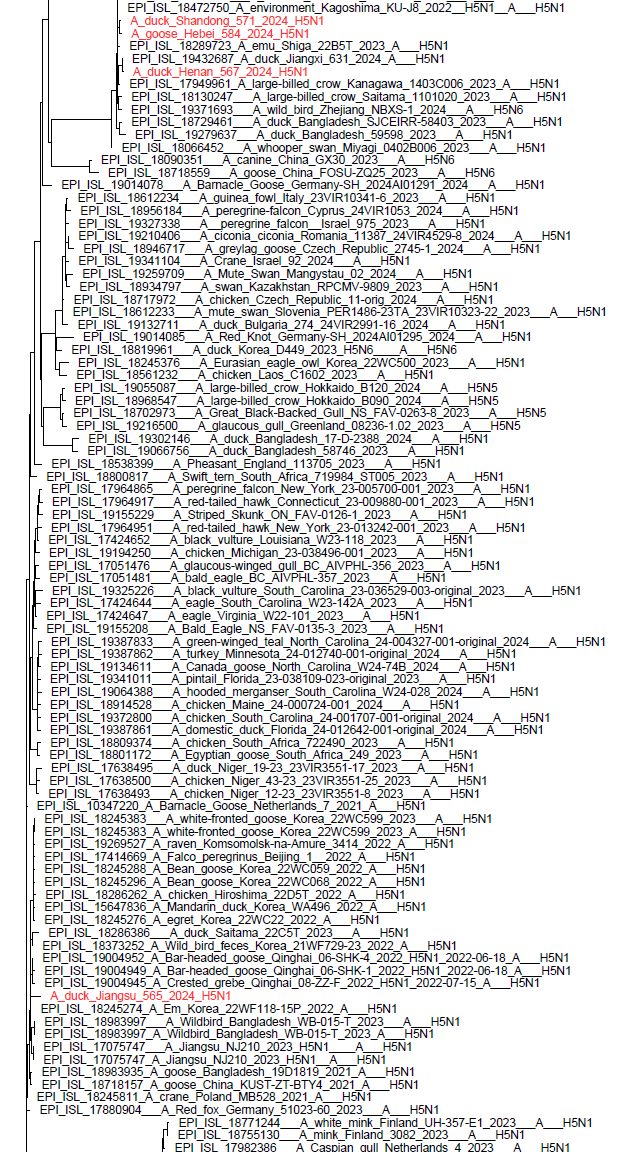


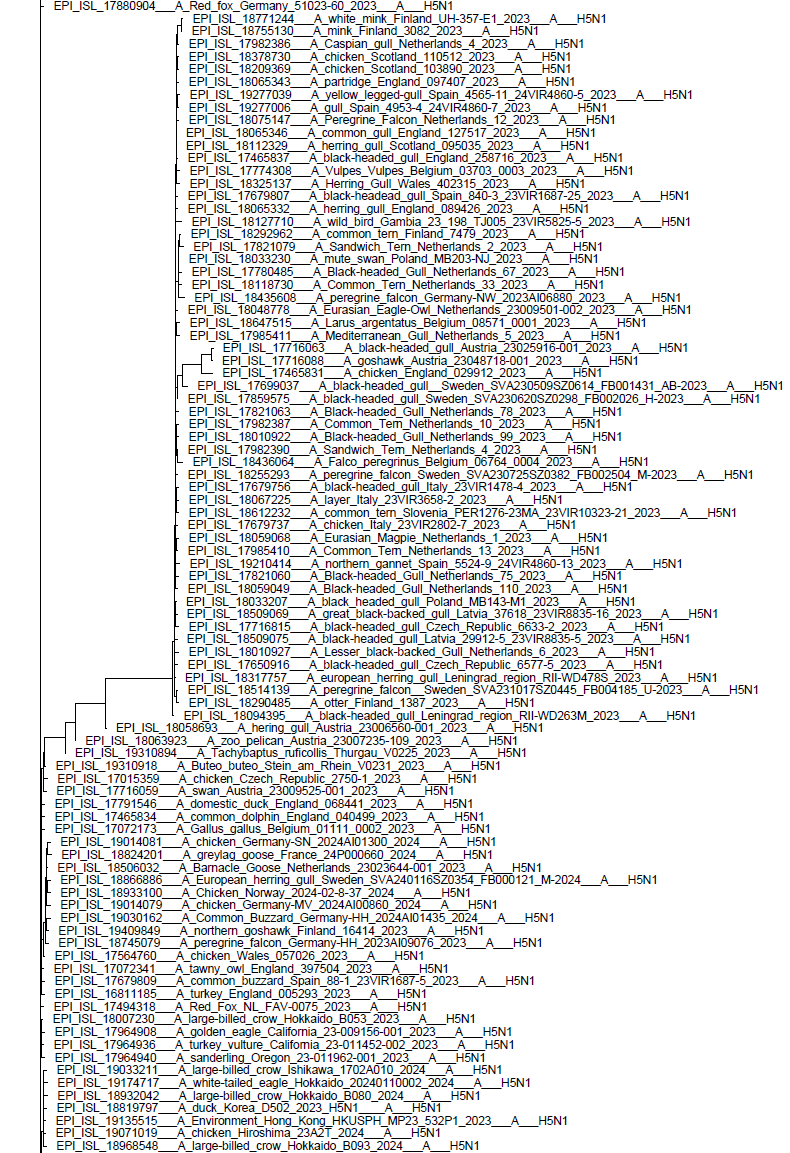


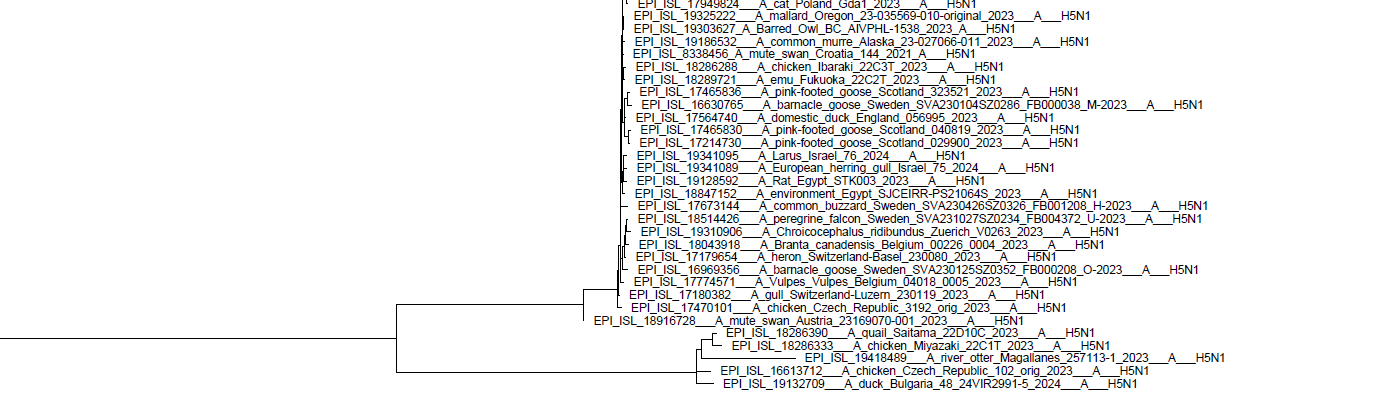

Supplement: Appendix Figure 8.docx [file TEMI_A_2505649_SM3523.docx]
