# Supplementary material for: Clade 2.3.4.4b highly pathogenic H5N1 influenza viruses from birds in China replicate effectively in bovine cells and pose potential public health risk
Source: Emerg Microbes Infect. 2025 May 12;14(1):2505649. doi: 10.1080/22221751.2025.2505649 (PMC12128135; doi:10.1080/22221751.2025.2505649)
Supplement: Appendix Table 3.docx [file TEMI_A_2505649_SM3533.docx]

**Appendix Table 3. The GISAID Numbers in Avian Influenza H5N1 Strain Homology Comparison.**

| Number | Viruses | Isolate ID |
| --- | --- | --- |
| 1 | A/bean goose/Korea/22WC200/2022(H5N1) | EPI_ISL_18245320 |
| 2 | A/chicken/Kagawa/22C2T/2022(H5N1) | EPI_ISL_18286299 |
| 3 | A/chicken/Kagawa/22D9T/2022(H5N1) | EPI_ISL_18286301 |
| 4 | A/chicken/Korea/C004/2023(H5N1) | EPI_ISL_19453498 |
| 5 | A/chicken/Korea/C064/2023(H5N1) | EPI_ISL_19453505 |
| 6 | A/duck/Korea/H515/2022(H5N1) | EPI_ISL_19453442 |
| 7 | A/chicken/Korea/H751/2022(H5N1) | EPI_ISL_19453492 |
| 8 | A/chicken/Niigata/22C3T/2023(H5N1) | EPI_ISL_18286339 |
| 9 | A/chicken/Niigata/22C6T/2023(H5N1) | EPI_ISL_18286340 |
| 10 | A/chicken/Niigata/22E12T/2023(H5N1) | EPI_ISL_18286344 |
| 11 | A/chicken/Okinawa/22A7T/2022(H5N1) | EPI_ISL_18286362 |
| 12 | A/chicken/Yamagata/22A5T/2022(H5N1) | EPI_ISL_18286376 |
| 13 | A/common buzzard/Korea/22WC336/2023(H5N1) | EPI_ISL_18245346 |
| 14 | A/common teal/Korea/WA709/2022(H5N1) | EPI_ISL_19453432 |
| 15 | A/duck/Korea/D114/2023(H5N1) | EPI_ISL_19453509 |
| 16 | A/duck/Korea/D195/2023(H5N1) | EPI_ISL_19453513 |
| 17 | A/duck/Korea/H515/2022(H5N1) | EPI_ISL_19453442 |
| 18 | A/duck/Okayama/22D3T/2022(H5N1) | EPI_ISL_18286384 |
| 19 | A/egret/Korea/22WC188/2022(H5N1) | EPI_ISL_18245318 |
| 20 | A/egret/Korea/22WC281/2022(H5N1) | EPI_ISL_18245335 |
| 21 | A/environment/Kagoshima/KU-B3/2022 (H5N1) | EPI_ISL_18472734 |
| 22 | A/environment/Kagoshima/KU-G3/2022(H5N1) | EPI_ISL_18472738 |
| 23 | A/hooded crane/Kagoshima/KU-75/2022(H5N1) | EPI_ISL_18472651 |
| 24 | A/hooded crane/Korea/22WC064/2022(H5N1) | EPI_ISL_18245295 |
| 25 | A/mallard/Korea/21WS41-5/2022(H5N1) | EPI_ISL_18373244 |
| 26 | A/northern goshawk/Shizuoka/2201D004/2022(H5N1) | EPI_ISL_18066455 |
| 27 | A/spot-billed duck/Korea/K22-862-1/2022(H5N1) | EPI_ISL_15944665 |
| 28 | A/white-fronted goose/Korea/22WC116/2022(H5N1) | EPI_ISL_18245307 |
| 29 | A/white-fronted goose/Korea/22WC401-3P/2023(H5N1) | EPI_ISL_18245357 |
| 30 | A/white-naped crane/Kagoshima/KU-178/2022 (H5N1) | EPI_ISL_18472709 |
| 31 | A/wild bird/Korea/H588-4/2022(H5N1) | EPI_ISL_19453436 |
| 32 | A/wild duck/Korea/H536-4/2022(H5N1) | EPI_ISL_19453427 |
| 33 | A/wild duck/Korea/H600-1/2022(H5N1) | EPI_ISL_19453438 |
| 34 | A/Em/Korea/22WF118-15P/2022(H5N1) | EPI_ISL_18245274 |
| 35 | A/Em/Korea/22WF123-24P/2022(H5N1) | EPI_ISL_18245275 |
| 36 | A/Em/Korea/22WF167-12P/2022(H5N1) | EPI_ISL_18245283 |
| 37 | A/dairy cow/New Mexico/24-009306-003/2024 | EPI_ISL_19175606 |
| 38 | A/dairy cow/Texas/24_009367-002/2024 | EPI_ISL_19094613 |
| 39 | A/dairy cow/Kansas/24_009497-004/2024 | EPI_ISL_19094717 |
