# Supplementary material for: Clade 2.3.4.4b highly pathogenic H5N1 influenza viruses from birds in China replicate effectively in bovine cells and pose potential public health risk
Source: Emerg Microbes Infect. 2025 May 12;14(1):2505649. doi: 10.1080/22221751.2025.2505649 (PMC12128135; doi:10.1080/22221751.2025.2505649)
Supplement: Appendix Table 1.docx [file TEMI_A_2505649_SM3532.docx]

**Appendix Table 1. The GISAID numbers of the whole genomes of H5N1 influenza viruses.**

|  | A/duck/Jiangsu/565/2024 (H5N1) | A/duck/Henan/567/2024 (H5N1) | A/duck/Shandong/571/2024 | A/goose/Hebei/584/2024 (H5N1) |
| --- | --- | --- | --- | --- |
| Isolate ID | EPI_ISL_19468754 | EPI_ISL_19468755 | EPI_ISL_19468756 | EPI_ISL_19468757 |
| PB2 | EPI3597108 | EPI3597109 | EPI3597117 | EPI3597125 |
| PB1 | EPI3597133 | EPI3597110 | EPI3597118 | EPI3597126 |
| PA | EPI3597134 | EPI3597111 | EPI3597119 | EPI3597127 |
| HA | EPI3597135 | EPI3597112 | EPI3597120 | EPI3597128 |
| NP | EPI3597136 | EPI3597113 | EPI3597121 | EPI3597129 |
| NA | EPI3597137 | EPI3597114 | EPI3597122 | EPI3597130 |
| M | EPI3597138 | EPI3597115 | EPI3597123 | EPI3597131 |
| NS | EPI3597139 | EPI3597116 | EPI3597124 | EPI3597132 |
