# Supplementary material for: Clade 2.3.4.4b highly pathogenic H5N1 influenza viruses from birds in China replicate effectively in bovine cells and pose potential public health risk
Source: Emerg Microbes Infect. 2025 May 12;14(1):2505649. doi: 10.1080/22221751.2025.2505649 (PMC12128135; doi:10.1080/22221751.2025.2505649)
Supplement: Appendix Table 4.docx [file TEMI_A_2505649_SM3527.docx]

**Appendix Table 4. Compilation of Amino Acid Residues at Mammalian Adaptation Sites in Isolated Viral Strains**

| Site | Functional type | 565/H5N1 | 567/H5N1 | 571/H5N1 | 584/H5N1 |
| --- | --- | --- | --- | --- | --- |
| PB2-E627K [1] | increase pathogenicity | E | E | E | E |
| PB2-M631L [2] | increase mammal adapt | M | M | M | M |
| PB2-D701N [3] | increase pathogenicity | D | D | D | D |
| PB2-S714R [3] | promote adaptation  to a mammalian host | S | S | S | S |
| PB2-T271A [4] | promote adaptation  to a mammalian host | T | T | T | T |
| PB2-V495I [5] | increase mammal adapt | V | V | V | V |
| PB1-T296R [6] | enhance polymerase activity | T | T | T | T |
| HA-E79K [12] | increase human adapt | E | E | E | E |
| HA-S125F [12] | change human adapt | S | S | S | S |
| HA-Q142R [12] | change pathogenicity | Q | Q | Q | Q |
| HA-A160T [12] | decrease human adapt | A | A | A | A |
| HA-T199I [7] | increases receptor binding breadth | T | T | T | T |
| HA-N197T [12] | change human adapt | N | N | N | N |
| HA Q222K/R [12] | change virulence | Q | Q | Q | Q |
| HA-N224K [8] | adapted to the mammalian host | N | N | N | N |
| HA-Q226L [9] | adapted to the mammalian host | Q | Q | Q | Q |
| HA-G228-R [12] | increase human adapt | G | G | G | G |
| HA-S324N [12] | change pathogenicity | S | S | S | S |
| HA-P335L [12] | increase virulence | P | P | P | P |
| NA-A55T [12] | virulence / host range | T | T | T | T |
| NA-E57G [12] | virulence / host range | E | E | E | E |
| NA-V67I [12] | virulence / host range | V | V | V | V |
| NA-N70S/D [12] | virulence / host range | N | N | N | N |
| NA-N71S [12] | virulence / host range | N | N | N | N |
| NA-E119V [10] | resistant phenotype | E | E | E | E |
| NA-R150K [10] | resistant phenotype | K | K | K | K |
| NA-H275Y [10] | resistant phenotype | H | H | H | H |
| NA-T438A/I [12] | increase antiviral resist | T | T | T | T |
| ‌NP-D375N [11] | mediate viral escape from the importin-α7 mediated nuclear import pathway | D | D | D | D |
| NP-V105K [11] | mediate viral escape from the importin-α7 mediated nuclear import pathway | V | V | V | V |
| NP-G102R [11] | mediate viral escape from the importin-α7 mediated nuclear import pathway | G | G | G | G |
| NS-E229K [12] | change virulence | E | E | E | E |
| NS1-P42S [12] | adapted to the mammalian host | S | S | S | S |
| M-R77K [12] | change pathogenicity | R | R | R | R |
| M-S207G [12] | decrease pathogenicity | S | S | S | S |

# **References**

[1] Gu C, Maemura T, Guan L, et al. A human isolate of bovine H5N1 is transmissible and lethal in animal models. Nature. 2024 Dec;636(8043):711-718.

[2] Idoko-Akoh A, Goldhill DH, Sheppard CM, et al. Creating resistance to avian influenza infection through genome editing of the ANP32 gene family. Nat Commun. 2023 Oct 10;14(1):6136.

[3] Czudai-Matwich V, Otte A, Matrosovich M, et al. PB2 mutations D701N and S714R promote adaptation of an influenza H5N1 virus to a mammalian host. J Virol. 2014 Aug;88(16):8735-42.

[4] Restori KH, Septer KM, Field CJ, et al. Risk assessment of a highly pathogenic H5N1 influenza virus from mink. Nat Commun. 2024 May 15;15(1):4112.

[5] Nguyen T-Q, Hutter C, Markin A, et al. 2024.

[6] Yu Z, Cheng K, Sun W, et al. A PB1 T296R substitution enhance polymerase activity and confer a virulent phenotype to a 2009 pandemic H1N1 influenza virus in mice. Virology. 2015 Dec;486:180-6.

[7] Good MR, Fernández-Quintero ML, Ji W, et al. A single mutation in dairy cow-associated H5N1 viruses increases receptor binding breadth. Nat Commun. 2024 Dec 30;15(1):10768.

[8] Lin TH, Zhu X, Wang S, et al. A single mutation in bovine influenza H5N1 hemagglutinin switches specificity to human receptors. Science. 2024 Dec 6;386(6726):1128-1134.

[9] Liu Q, Zhou B, Ma W, et al. Analysis of recombinant H7N9 wild-type and mutant viruses in pigs shows that the Q226L mutation in HA is important for transmission. J Virol. 2014 Jul;88(14):8153-65.

[10] Treurnicht FK, Buys A, Tempia S, et al. Replacement of neuraminidase inhibitor-susceptible influenza A(H1N1) with resistant phenotype in 2008 and circulation of susceptible influenza A and B viruses during 2009-2013, South Africa. Influenza Other Respir Viruses. 2019 Jan;13(1):54-63.

[11] Resa-Infante P, Bonet J, Thiele S, et al. Alternative interaction sites in the influenza A virus nucleoprotein mediate viral escape from the importin-α7 mediated nuclear import pathway. Febs j. 2019 Sep;286(17):3374-3388.

[12] Nguyen T-Q, Hutter C, Markin A, et al. 2024. Emergence and interstate spread of highly pathogenic avian influenza A(H5N1) in dairy cattle. *bioRxiv*.
