# Supplementary material for: Clade 2.3.4.4b highly pathogenic H5N1 influenza viruses from birds in China replicate effectively in bovine cells and pose potential public health risk
Source: Emerg Microbes Infect. 2025 May 12;14(1):2505649. doi: 10.1080/22221751.2025.2505649 (PMC12128135; doi:10.1080/22221751.2025.2505649)
Supplement: Appendix Figure 10.docx [file TEMI_A_2505649_SM3526.docx]

**A** Percent Identity

**———————————————————————————————————**

|  | **1** | **2** | **3** | **4** | **5** | **6** |  |
| --- | --- | --- | --- | --- | --- | --- | --- |
| **1** |  | 91.9 | 92.1 | 92.0 | 99.0 | 92.4 | **1** |
| **2** | 8.7 |  | 98.7 | 98.6 | 92.2 | 99.3 | **2** |
| **3** | 8.4 | 1.3 |  | 99.5 | 92.4 | 99.4 | **3** |
| **4** | 8.6 | 1.4 | 0.5 |  | 92.3 | 99.3 | **4** |
| **5** | 1.0 | 8.3 | 8.1 | 8.3 |  | 92.7 | **5** |
| **6** | 8.2 | 0.7 | 0.6 | 0.7 | 7.8 |  | **6** |
|  | **1** | **2** | **3** | **4** | **5** | **6** |  |


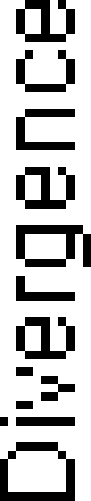


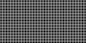

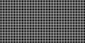

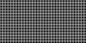

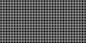

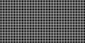

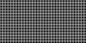


## PB2

A-duck-Jiangsu-565-2024-H5N1 A-duck-Henan-567-2024-H5N1

A-duck-Shandong-571-2024-H5N1 A-goose-Hebei-584-2024-H5N1

A-chicken-Kagawa-22C2T-2022-H5N1 A-chicken-Korea-C004-2023-H5N1

# B

Percent Identity

**——————————————————————————————————————————————————————————————**

|  | **1** | **2** | **3** | **4** | **5** | **6** | **7** | **8** | **9** | **10** | **11** | **12** |  |
| --- | --- | --- | --- | --- | --- | --- | --- | --- | --- | --- | --- | --- | --- |
| **1** |  | 92.1 | 92.3 | 92.1 | 99.2 | 99.2 | 99.2 | 99.1 | 92.5 | 92.4 | 92.6 | 92.5 | **1** |
| **2** | 8.5 |  | 98.7 | 98.5 | 92.6 | 92.5 | 92.5 | 92.5 | 99.4 | 99.3 | 99.3 | 99.2 | **2** |
| **3** | 8.3 | 1.3 |  | 99.6 | 92.7 | 92.6 | 92.6 | 92.6 | 99.1 | 99.0 | 99.4 | 99.3 | **3** |
| **4** | 8.5 | 1.6 | 0.4 |  | 92.5 | 92.4 | 92.4 | 92.4 | 98.8 | 98.8 | 99.1 | 99.1 | **4** |
| **5** | 0.8 | 7.9 | 7.8 | 8.0 |  | 99.9 | 99.9 | 99.8 | 93.0 | 92.9 | 93.1 | 93.0 | **5** |
| **6** | 0.8 | 8.0 | 7.9 | 8.1 | 0.1 |  | 99.9 | 100.0 | 92.9 | 92.8 | 93.0 | 92.9 | **6** |
| **7** | 0.8 | 8.0 | 7.9 | 8.1 | 0.1 | 0.1 |  | 99.9 | 92.9 | 92.8 | 93.0 | 92.9 | **7** |
| **8** | 0.9 | 8.1 | 8.0 | 8.2 | 0.2 | 0.0 | 0.1 |  | 92.8 | 92.8 | 92.9 | 92.9 | **8** |
| **9** | 8.1 | 0.6 | 0.9 | 1.2 | 7.5 | 7.6 | 7.6 | 7.7 |  | 100.0 | 99.6 | 99.6 | **9** |
| **10** | 8.1 | 0.7 | 1.0 | 1.2 | 7.6 | 7.7 | 7.7 | 7.7 | 0.0 |  | 99.6 | 99.6 | **10** |
| **11** | 8.0 | 0.8 | 0.6 | 0.9 | 7.4 | 7.5 | 7.5 | 7.6 | 0.4 | 0.4 |  | 100.0 | **11** |
| **12** | 8.0 | 0.8 | 0.7 | 0.9 | 7.5 | 7.6 | 7.6 | 7.6 | 0.4 | 0.4 | 0.0 |  | **12** |
|  | **1** | **2** | **3** | **4** | **5** | **6** | **7** | **8** | **9** | **10** | **11** | **12** |  |


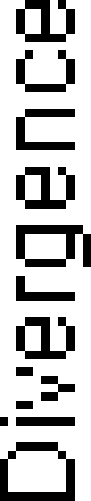


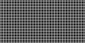

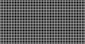

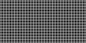

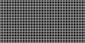

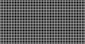

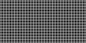

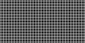

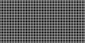

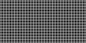

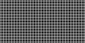

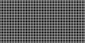

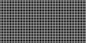


## PB1

A-duck-Jiangsu-565-2024-H5N1 A-duck-Henan-567-2024-H5N1

A-duck-Shandong-571-2024-H5N1 A-goose-Hebei-584-2024-H5N1

A-white-fronted_goose-Korea-22WC116-2022-H5N1 A-Em-Korea-22WF167-12P-2022-H5N1

A-Em-Korea-22WF123-24P-2022-H5N1

A-wild_bird-Korea-H588-4-2022-H5N1 A-duck-Korea-D114-2023-H5N1

A-chicken-Korea-C064-2023-H5N1

A-common_buzzard-Korea-22WC336-2023-H5N A-common_teal-Korea-WA709-2022-H5N1

Percent Identity

**——————————————————————————————————————————————————————————————**

|  | **1** | **2** | **3** | **4** | **5** | **6** | **7** | **8** | **9** | **10** | **11** | **12** |  |
| --- | --- | --- | --- | --- | --- | --- | --- | --- | --- | --- | --- | --- | --- |
| **1** |  | 95.6 | 96.0 | 96.0 | 98.9 | 96.3 | 96.2 | 96.3 | 99.0 | 96.2 | 96.2 | 96.2 | **1** |
| **2** | 4.6 |  | 98.8 | 98.8 | 96.3 | 99.3 | 99.3 | 99.3 | 96.3 | 99.3 | 99.3 | 99.3 | **2** |
| **3** | 4.2 | 1.2 |  | 99.6 | 96.6 | 99.3 | 99.3 | 99.3 | 96.7 | 99.3 | 99.3 | 99.3 | **3** |
| **4** | 4.1 | 1.2 | 0.4 |  | 96.7 | 99.3 | 99.3 | 99.3 | 96.7 | 99.2 | 99.3 | 99.3 | **4** |
| **5** | 1.1 | 3.9 | 3.5 | 3.5 |  | 97.0 | 96.9 | 96.9 | 99.9 | 96.8 | 96.9 | 96.9 | **5** |
| **6** | 3.8 | 0.7 | 0.7 | 0.7 | 3.1 |  | 99.9 | 100.0 | 97.0 | 99.9 | 99.9 | 99.9 | **6** |
| **7** | 3.9 | 0.7 | 0.7 | 0.7 | 3.2 | 0.1 |  | 100.0 | 96.9 | 99.9 | 99.9 | 99.9 | **7** |
| **8** | 3.9 | 0.7 | 0.7 | 0.7 | 3.2 | 0.0 | 0.0 |  | 97.0 | 99.9 | 100.0 | 100.0 | **8** |
| **9** | 1.0 | 3.8 | 3.5 | 3.4 | 0.1 | 3.1 | 3.2 | 3.1 |  | 96.9 | 96.9 | 96.9 | **9** |
| **10** | 3.9 | 0.7 | 0.7 | 0.8 | 3.3 | 0.1 | 0.1 | 0.1 | 3.2 |  | 99.9 | 99.9 | **10** |
| **11** | 3.9 | 0.7 | 0.7 | 0.7 | 3.2 | 0.1 | 0.1 | 0.0 | 3.2 | 0.1 |  | 99.9 | **11** |
| **12** | 3.9 | 0.7 | 0.7 | 0.7 | 3.2 | 0.1 | 0.1 | 0.0 | 3.2 | 0.1 | 0.1 |  | **12** |
|  | **1** | **2** | **3** | **4** | **5** | **6** | **7** | **8** | **9** | **10** | **11** | **12** |  |


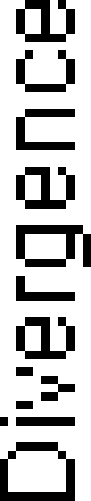


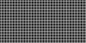

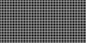

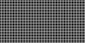

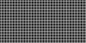

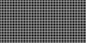

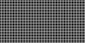

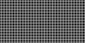

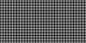

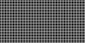

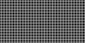

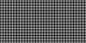

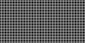


## PA

A-duck-Jiangsu-565-2024-H5N1 A-duck-Henan-567-2024-H5N1

A-duck-Shandong-571-2024-H5N1 A-goose-Hebei-584-2024-H5N1

A-egret-Korea-22WC188-2022-H5N1

A-wild_duck-Korea-H600-1-2022-H5N1

A-common_teal-Korea-WA709-2022-H5N1 A-wild_duck-Korea-H536-4-2022-H5N1

A-duck-Okayama-22D3T-2022-H5N1

A-northern_goshawk-Shizuoka-2201D004-20 A-chicken-Okinawa-22A7T-2022-H5N1

A-chicken-Niigata-22C6T-2023-H5N1

# D

Percent Identity

**—————————————————————————————————————————————————————————**

|  | **1** | **2** | **3** | **4** | **5** | **6** | **7** | **8** | **9** | **10** | **11** |  |
| --- | --- | --- | --- | --- | --- | --- | --- | --- | --- | --- | --- | --- |
| **1** |  | 97.6 | 97.7 | 97.5 | 98.3 | 98.3 | 98.3 | 98.2 | 98.2 | 98.2 | 98.0 | **1** |
| **2** | 2.5 |  | 98.7 | 98.5 | 99.2 | 99.2 | 99.1 | 99.2 | 99.2 | 99.2 | 99.0 | **2** |
| **3** | 2.4 | 1.3 |  | 99.5 | 99.4 | 99.4 | 99.2 | 99.4 | 99.5 | 99.4 | 99.4 | **3** |
| **4** | 2.6 | 1.5 | 0.5 |  | 99.2 | 99.2 | 99.1 | 99.3 | 99.3 | 99.2 | 99.0 | **4** |
| **5** | 1.7 | 0.8 | 0.6 | 0.8 |  | 99.9 | 99.8 | 99.9 | 99.9 | 99.9 | 99.7 | **5** |
| **6** | 1.7 | 0.8 | 0.6 | 0.8 | 0.1 |  | 99.8 | 99.9 | 99.9 | 99.9 | 99.7 | **6** |
| **7** | 1.7 | 0.9 | 0.8 | 0.9 | 0.2 | 0.2 |  | 99.8 | 99.8 | 99.8 | 99.6 | **7** |
| **8** | 1.8 | 0.8 | 0.6 | 0.7 | 0.1 | 0.1 | 0.2 |  | 99.9 | 99.9 | 99.7 | **8** |
| **9** | 1.8 | 0.8 | 0.5 | 0.7 | 0.1 | 0.1 | 0.2 | 0.1 |  | 99.9 | 99.7 | **9** |
| **10** | 1.8 | 0.8 | 0.6 | 0.8 | 0.1 | 0.1 | 0.2 | 0.1 | 0.1 |  | 99.8 | **10** |
| **11** | 2.0 | 1.0 | 0.6 | 1.0 | 0.3 | 0.3 | 0.4 | 0.3 | 0.3 | 0.2 |  | **11** |
|  | **1** | **2** | **3** | **4** | **5** | **6** | **7** | **8** | **9** | **10** | **11** |  |


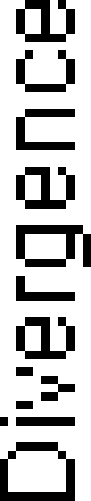


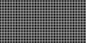

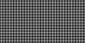

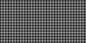

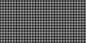

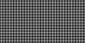

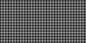

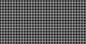

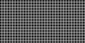

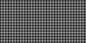

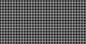

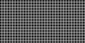


## HA

A-duck-Jiangsu-565-2024-H5N1 A-duck-Henan-567-2024-H5N1

A-duck-Shandong-571-2024-H5N1 A-goose-Hebei-584-2024-H5N1

A-white-naped_crane-Kagoshima-KU-178-20 A-chicken-Niigata-22E12T-2023-H5N1

A-chicken-Kagawa-22D9T-2022-H5N1 A-chicken-Yamagata-22A5T-2022-H5N1

A-hooded_crane-Kagoshima-KU-75-2022-H5N1 A-chicken-Korea-C004-2023-H5N1

A-duck-Korea-H515-2022-H5N1

# E

Percent Identity

**————————————————————————————————————————————————**

|  | **1** | **2** | **3** | **4** | **5** | **6** | **7** | **8** | **9** |  |
| --- | --- | --- | --- | --- | --- | --- | --- | --- | --- | --- |
| **1** |  | 97.9 | 97.7 | 97.7 | 99.4 | 99.4 | 99.3 | 99.3 | 98.2 | **1** |
| **2** | 2.2 |  | 99.2 | 99.2 | 98.2 | 98.3 | 98.3 | 98.3 | 99.7 | **2** |
| **3** | 2.3 | 0.8 |  | 99.9 | 98.1 | 98.2 | 98.1 | 98.1 | 99.5 | **3** |
| **4** | 2.3 | 0.8 | 0.1 |  | 98.1 | 98.2 | 98.1 | 98.1 | 99.5 | **4** |
| **5** | 0.6 | 1.8 | 2.0 | 2.0 |  | 99.9 | 99.8 | 99.9 | 98.5 | **5** |
| **6** | 0.6 | 1.7 | 1.8 | 1.8 | 0.1 |  | 99.9 | 99.9 | 98.7 | **6** |
| **7** | 0.6 | 1.7 | 1.8 | 1.8 | 0.1 | 0.0 |  | 99.9 | 98.6 | **7** |
| **8** | 0.7 | 1.8 | 1.9 | 1.9 | 0.1 | 0.1 | 0.1 |  | 98.6 | **8** |
| **9** | 1.8 | 0.3 | 0.5 | 0.5 | 1.5 | 1.4 | 1.4 | 1.4 |  | **9** |
|  | **1** | **2** | **3** | **4** | **5** | **6** | **7** | **8** | **9** |  |


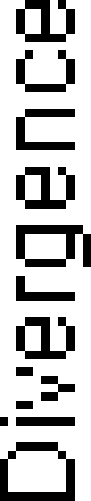


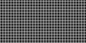

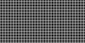

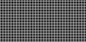

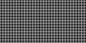

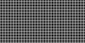

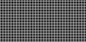

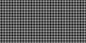

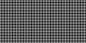

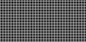


## NP

A-duck-Jiangsu-565-2024-H5N1 A-duck-Henan-567-2024-H5N1

A-duck-Shandong-571-2024-H5N1 A-goose-Hebei-584-2024-H5N1

A-Bean_goose-Korea-22WC200-2022-H5N1 A-Em-Korea-22WF167-12P-202-H5N1

A-Spot-billed_duck-Korea-K22-862-1-2022 A-wild_bird-Korea-H588-4-2022-H5N1

A-common_teal-Korea-WA709-2022-H5N1

# F

Percent Identity

**——————————————————————————————————————————————————————————————————**

|  | **1** | **2** | **3** | **4** | **5** | **6** | **7** | **8** | **9** | **10** | **11** | **12** | **13** |  |
| --- | --- | --- | --- | --- | --- | --- | --- | --- | --- | --- | --- | --- | --- | --- |
| **1** |  | 98.0 | 97.7 | 97.8 | 98.9 | 98.7 | 98.7 | 98.7 | 98.5 | 98.6 | 98.4 | 98.4 | 98.4 | **1** |
| **2** | 2.0 |  | 98.7 | 98.8 | 99.0 | 98.9 | 98.9 | 99.4 | 99.5 | 99.4 | 99.4 | 99.4 | 99.4 | **2** |
| **3** | 2.4 | 1.4 |  | 99.4 | 98.7 | 98.5 | 98.4 | 99.0 | 99.1 | 99.1 | 99.1 | 99.2 | 99.1 | **3** |
| **4** | 2.2 | 1.2 | 0.6 |  | 98.8 | 98.7 | 98.6 | 99.1 | 99.3 | 99.2 | 99.3 | 99.4 | 99.3 | **4** |
| **5** | 1.1 | 1.0 | 1.4 | 1.2 |  | 99.7 | 99.6 | 99.6 | 99.5 | 99.6 | 99.4 | 99.4 | 99.4 | **5** |
| **6** | 1.3 | 1.1 | 1.5 | 1.4 | 0.3 |  | 99.5 | 99.5 | 99.4 | 99.4 | 99.2 | 99.3 | 99.2 | **6** |
| **7** | 1.4 | 1.1 | 1.6 | 1.4 | 0.4 | 0.5 |  | 99.4 | 99.3 | 99.4 | 99.1 | 99.2 | 99.1 | **7** |
| **8** | 1.4 | 0.6 | 1.0 | 0.9 | 0.4 | 0.5 | 0.6 |  | 99.9 | 99.9 | 99.7 | 99.8 | 99.7 | **8** |
| **9** | 1.5 | 0.5 | 0.9 | 0.7 | 0.5 | 0.6 | 0.7 | 0.1 |  | 99.9 | 99.9 | 99.9 | 99.9 | **9** |
| **10** | 1.4 | 0.6 | 0.9 | 0.8 | 0.4 | 0.6 | 0.6 | 0.1 | 0.1 |  | 99.8 | 99.9 | 99.8 | **10** |
| **11** | 1.7 | 0.6 | 0.9 | 0.7 | 0.6 | 0.8 | 0.9 | 0.3 | 0.1 | 0.2 |  | 99.9 | 99.9 | **11** |
| **12** | 1.6 | 0.6 | 0.8 | 0.6 | 0.6 | 0.7 | 0.8 | 0.2 | 0.1 | 0.1 | 0.1 |  | 99.9 | **12** |
| **13** | 1.7 | 0.6 | 0.9 | 0.7 | 0.6 | 0.8 | 0.9 | 0.3 | 0.1 | 0.2 | 0.1 | 0.1 |  | **13** |
|  | **1** | **2** | **3** | **4** | **5** | **6** | **7** | **8** | **9** | **10** | **11** | **12** | **13** |  |

A-duck-Jiangsu-565-2024-H5N1 A-duck-Henan-567-2024-H5N1


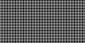

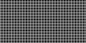

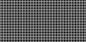

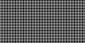

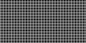

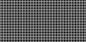

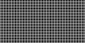

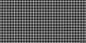

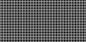

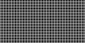

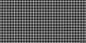

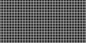

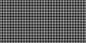


A-duck-Shandong-571-2024-H5N1 A-goose-Hebei-584-2024-H5N1


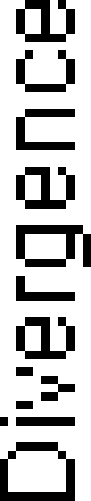
A-Em-Korea-22WF118-15P-2022-H5N1 A-Mallard-Korea-21WS41-5-2022-H5N1

A-wild_duck-Korea-H503-2022-H5N1

A-Hooded_crane-Korea-22WC064-2022-H5 A-environment-Kagoshima-KU-B3-2022-H5 A-duck-Korea-H515-2022-H5N1

A-duck-Korea-D195-2023-H5N1

A-chicken-Niigata-22C3T-2023-H5N1

A-common_teal-Korea-WA709-2022-H5N1

NA

# G

Percent Identity

**—————————————————————————————————————————————————————**

|  | **1** | **2** | **3** | **4** | **5** | **6** | **7** | **8** | **9** | **10** |  |
| --- | --- | --- | --- | --- | --- | --- | --- | --- | --- | --- | --- |
| **1** |  | 98.5 | 98.7 | 98.7 | 99.2 | 99.1 | 99.0 | 99.0 | 98.9 | 98.9 | **1** |
| **2** | 1.5 |  | 99.0 | 99.0 | 99.3 | 99.4 | 99.5 | 99.3 | 99.2 | 99.2 | **2** |
| **3** | 1.3 | 1.0 |  | 99.8 | 99.5 | 99.6 | 99.5 | 99.7 | 99.4 | 99.4 | **3** |
| **4** | 1.3 | 1.0 | 0.2 |  | 99.5 | 99.6 | 99.5 | 99.5 | 99.6 | 99.6 | **4** |
| **5** | 0.8 | 0.7 | 0.5 | 0.5 |  | 99.9 | 99.8 | 99.8 | 99.7 | 99.7 | **5** |
| **6** | 0.9 | 0.6 | 0.4 | 0.4 | 0.1 |  | 99.9 | 99.9 | 99.8 | 99.8 | **6** |
| **7** | 1.0 | 0.5 | 0.5 | 0.5 | 0.2 | 0.1 |  | 99.8 | 99.7 | 99.7 | **7** |
| **8** | 1.0 | 0.7 | 0.3 | 0.5 | 0.2 | 0.1 | 0.2 |  | 99.7 | 99.7 | **8** |
| **9** | 1.0 | 0.7 | 0.5 | 0.3 | 0.2 | 0.1 | 0.2 | 0.2 |  | 99.9 | **9** |
| **10** | 1.1 | 0.8 | 0.6 | 0.4 | 0.3 | 0.2 | 0.3 | 0.3 | 0.0 |  | **10** |
|  | **1** | **2** | **3** | **4** | **5** | **6** | **7** | **8** | **9** | **10** |  |


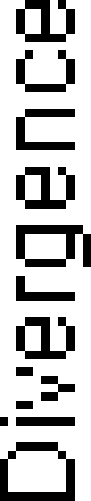


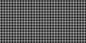

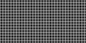

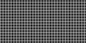

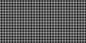

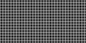

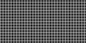

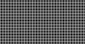

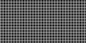

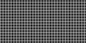

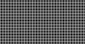


## M

A-duck-Jiangsu-565-2024-H5N1 A-duck-Henan-567-2024-H5N1

A-duck-Shandong-571-2024-H5N1 A-goose-Hebei-584-2024-H5N1

A-white-fronted_goose-Korea-22WC116-2022-H5N1 A-duck-Korea-D195-2023-H5N1

A-white-fronted_goose-Korea-22WC401-3P-2023-H5N1 A-egret-Korea-22WC281-2022-H5N1

A-environment-Kagoshima-KU-G3-2022-H5N1 A-chicken-Korea-H751-2022-H5N1

# H

Percent Identity

**———————————————————————————————————**

|  | **1** | **2** | **3** | **4** | **5** | **6** |  |
| --- | --- | --- | --- | --- | --- | --- | --- |
| **1** |  | 89.9 | 89.7 | 89.6 | 98.8 | 90.1 | **1** |
| **2** | 11.1 |  | 98.9 | 98.8 | 90.8 | 99.3 | **2** |
| **3** | 11.2 | 1.1 |  | 99.6 | 90.7 | 99.6 | **3** |
| **4** | 11.4 | 1.2 | 0.4 |  | 90.6 | 99.5 | **4** |
| **5** | 1.2 | 10.0 | 10.1 | 10.2 |  | 91.1 | **5** |
| **6** | 10.8 | 0.7 | 0.4 | 0.5 | 9.7 |  | **6** |
|  | **1** | **2** | **3** | **4** | **5** | **6** |  |


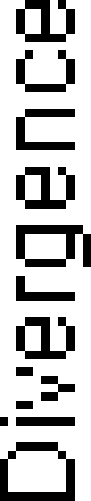


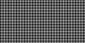

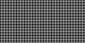

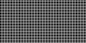

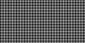

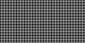

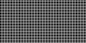


## NS

A-duck-Jiangsu-565-2024-H5N1 A-duck-Henan-567-2024-H5N1

A-duck-Shandong-571-2024-H5N1 A-goose-Hebei-584-2024-H5N1

A-Em-Korea-22WF118-15P-2022-H5N1 A-duck-Korea-D114-2023-H5N1

# I

Percent Identity

**———————————————————————————————————————**

|  | **1** | **2** | **3** | **4** | **5** | **6** | **7** |  |
| --- | --- | --- | --- | --- | --- | --- | --- | --- |
| **1** |  | 97.6 | 97.7 | 97.5 | 96.0 | 96.0 | 96.0 | **1** |
| **2** | 2.5 |  | 98.7 | 98.5 | 96.7 | 96.7 | 96.6 | **2** |
| **3** | 2.4 | 1.3 |  | 99.5 | 96.6 | 96.6 | 96.5 | **3** |
| **4** | 2.6 | 1.5 | 0.5 |  | 96.4 | 96.4 | 96.4 | **4** |
| **5** | 4.1 | 3.4 | 3.5 | 3.7 |  | 100.0 | 99.9 | **5** |
| **6** | 4.1 | 3.4 | 3.5 | 3.7 | 0.0 |  | 99.9 | **6** |
| **7** | 4.2 | 3.5 | 3.6 | 3.8 | 0.1 | 0.1 |  | **7** |
|  | **1** | **2** | **3** | **4** | **5** | **6** | **7** |  |


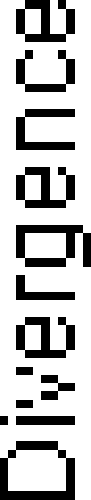


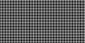

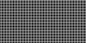

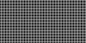

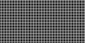

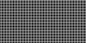

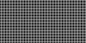

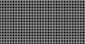


## HA

A-duck-Jiangsu-565-2024-H5N1 A-duck-Henan-567-2024-H5N1

A-duck-Shandong-571-2024-H5N1 A-goose-Hebei-584-2024-H5N1

A-dairy cow-New Mexico-24-009306-003-2024-H5N1 A-dairy cow-Texas-24_009367-002-2024-H5N1

A-dairy cow-Kansas-24_009497-004-2024-H5N1

**J** Percent Identity

**————————————————————————————————————————**

|  | **1** | **2** | **3** | **4** | **5** | **6** | **7** |  |
| --- | --- | --- | --- | --- | --- | --- | --- | --- |
| **1** |  | 98.0 | 97.7 | 97.8 | 96.6 | 96.6 | 96.5 | **1** |
| **2** | 2.0 |  | 98.7 | 98.8 | 96.7 | 96.6 | 96.6 | **2** |
| **3** | 2.4 | 1.4 |  | 99.4 | 96.5 | 96.4 | 96.4 | **3** |
| **4** | 2.2 | 1.2 | 0.6 |  | 96.7 | 96.5 | 96.5 | **4** |
| **5** | 3.5 | 3.4 | 3.6 | 3.4 |  | 99.7 | 99.9 | **5** |
| **6** | 3.5 | 3.5 | 3.7 | 3.6 | 0.3 |  | 99.7 | **6** |
| **7** | 3.7 | 3.5 | 3.7 | 3.6 | 0.1 | 0.3 |  | **7** |
|  | **1** | **2** | **3** | **4** | **5** | **6** | **7** |  |


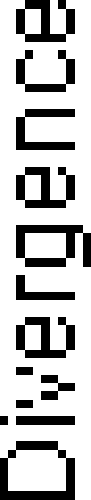


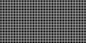

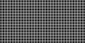

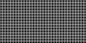

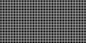


## NA

A-duck-Jiangsu-565-2024-H5N1 A-duck-Henan-567-2024-H5N1

A-duck-Shandong-571-2024-H5N1 A-goose-Hebei-584-2024-H5N1

A-dairy cow-New Mexico-24-009306-003-2024-H5N1

A-dairy cow-Texas-24_009367-002-2024-H5N1 A-dairy cow-Kansas-24_009497-004-2024-H5N1
