# Supplementary material for: Clade 2.3.4.4b highly pathogenic H5N1 influenza viruses from birds in China replicate effectively in bovine cells and pose potential public health risk
Source: Emerg Microbes Infect. 2025 May 12;14(1):2505649. doi: 10.1080/22221751.2025.2505649 (PMC12128135; doi:10.1080/22221751.2025.2505649)
Supplement: Appendix Table 2.docx [file TEMI_A_2505649_SM3522.docx]

**Appendix Table 2. Sample Sizes and Positivity Rates of Chickens, Ducks, and Geese in Live Poultry Markets Across Seven Provinces in China**

| Group  (Sampling event) | | Number of samples | | |
| --- | --- | --- | --- | --- |
|  |  | Chickens (a) | Ducks (a) | Goose (a) |
| Hebei (1) | | 20 (6,30%) | 20 (4,20%) | 11 (1,9.1%) |
| Shandong (1) | | 21 (3,14.3%) | 20 (3,15%) | 10 (0,0%) |
| Henan (2) | | 46 (8,17.4%) | 38 (5,13.2%) | 31 (3,9.7%) |
| Jiangsu (3) | | 63 (9,14.3%) | 36 (4,11.1%) | 36 (2,5.6%) |
| Hunan (3) | | 56 (8,14.3%) | 30 (3,10%) | 30 (4,13.3%) |
| Guangxi (3) | | 63 (12,19%) | 36 (4,11.1%) | 36 (3,8.3%) |
| Guangdong  (12) | LPM-1 (4) | 80 (23,28.9%) | 80 (28,35%) | 40 (12,30%) |
|  | LPM-2 (4) | 80 (33,41.3%) | 80 (21,26.3%) | 40 (16,40%) |
|  | LPM-3 (4) | 80 (29,36.25%) | 80 (19,23.75%) | 40 (8,20%) |

a:(Number of positive samples, Positive rate)
